# Diastereoselective Synthesis of the HIV Protease Inhibitor Darunavir and Related Derivatives via a Titanium Tetrachloride-Mediated Asymmetric Glycolate Aldol Addition Reaction

**DOI:** 10.1021/acs.joc.4c01057

**Published:** 2024-06-25

**Authors:** Jordan M. Witte, Emmanuel Ayim, Christopher J. Sams, Jasmine B. Service, Caitlyn C. Kant, Lillian Bambalas, Daniel Wright, Austin Carter, Kelly Moran, Isabella G. Rohrig, Gregory M. Ferrence, Shawn R. Hitchcock

**Affiliations:** Department of Chemistry, Illinois State University, Normal, Illinois 61790-4160, United States

## Abstract

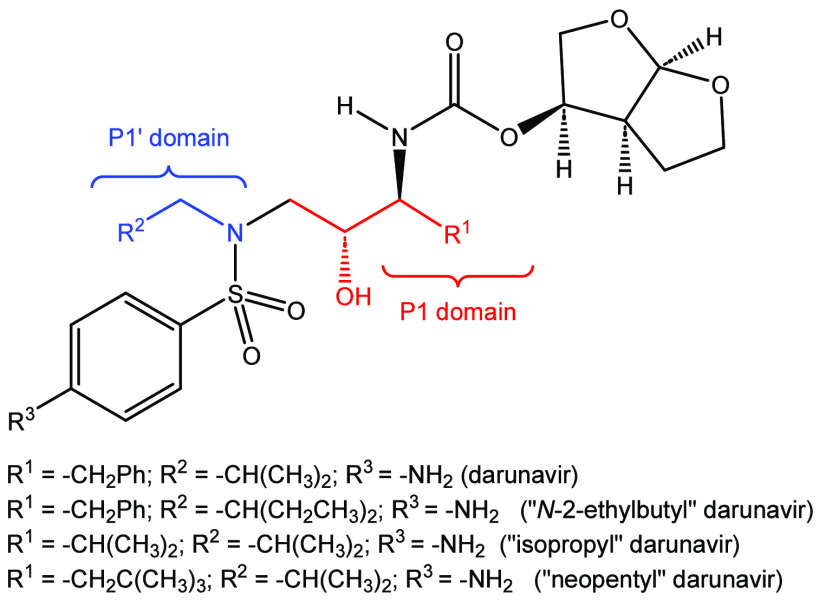

Darunavir is a potent HIV protease inhibitor that has been established as an effective tool in the fight against the progression of HIV/AIDS in the global community. The successful application of this drug has spurred the development of derivatives wherein strategic regions (e.g., P1, P1’, P2, and P2’) of the darunavir framework have been structurally modified. An alternate route for the synthesis of darunavir and three related P1 and P1’ derivatives has been developed. This synthetic pathway involves the use of a Crimmins titanium tetrachloride-mediated oxazolidine-2-thione-guided asymmetric glycolate aldol addition reaction. The resultant aldol adduct introduces the P1 fragment of darunavir via an aldehyde. Transamidation with a selected amine (isobutylamine or 2-ethyl-1-butylamine) to cleave the auxiliary yields an amide wherein the P1’ component is introduced. From this stage, the amide is reduced to the corresponding β-amino alcohol and the substrate is then bis-nosylated to introduce the requisite *p*-nitrobenzenesulfonamide component and activate the secondary alcohol for nucleophilic substitution. Treatment with sodium azide yielded the desired azides, and the deprotection of the *p*-methoxyphenoxy group is achieved with the use of ceric ammonium nitrate. Finally, hydrogenation to reduce both the aniline and azide functionalities with concurrent acylation yields darunavir and its derivatives.

## Introduction

**Figure 1:**
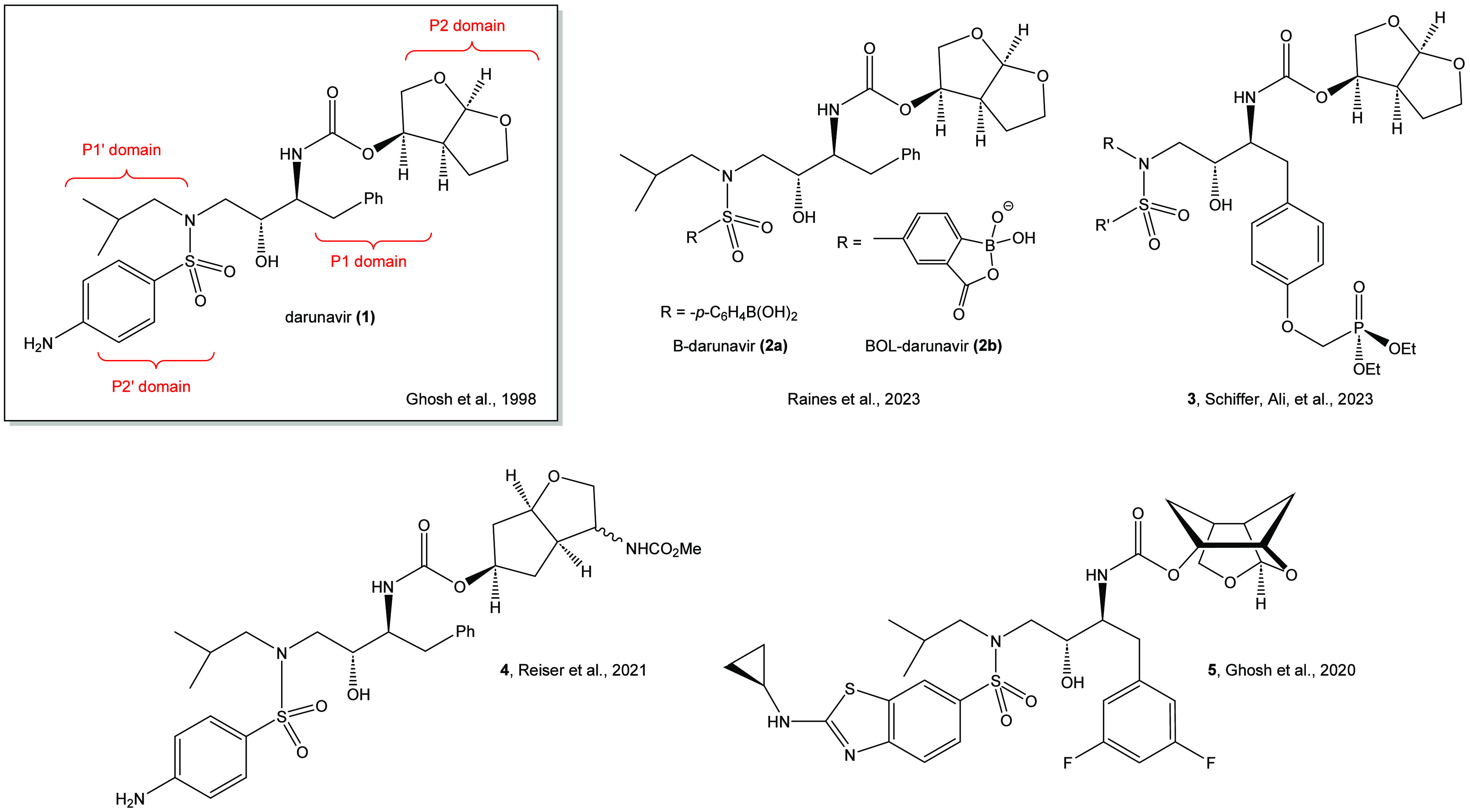
Darunavir (1) and related derivatives.

Darunavir **(1)** is a highly effective HIV protease inhibitor^1,2^ that is used in combination antiretroviral therapies (cART) along with other antiretroviral medicinal agents for suppressing viral replication and ultimately reducing the viral load in patients.^3^ This drug was initially approved by the FDA in 2006 and the European Directorate for the Quality of Medicines and Health Care (The European Pharmacopoeia) in 2007 and fully approved in 2008 due to its high efficacy in combating HIV infection.^4^ The successful application of darunavir in combination therapies has inspired the pursuit of improved syntheses of darunavir^5−9^ and syntheses of derivatives with the potential for even greater potency (Figure 1).^10−15^ In this context, the seminal efforts of Ghosh and coworkers laid the foundation for structural aspects of darunavir and the domains where the structure could be modified. In 2023, Raines and coworkers modified the structure of darunavir by modifying the P2’ region with the use of a benzoborolone group (see **2a** and **2b**) and demonstrated the utility of installing such groups as pharmacophores.^10^ Schiffer, Ali, and coworkers modified the P2 domain by introducing a phenolic methylene diethylphosphonate group in conjunction with the introduction of modifications of the P1’ and P2’ domains to afford darunavir derivative **3**.^11^ This modification resulted in sustained potency against highly resistant HIV-1 variants.^11^

Reiser and coworkers changed the P2 domain of darunavir by the use of an ethereal bicyclic cyclopentyl system resulting in low nanomolar IC_50_ values.^12^ Ghosh and coworkers have been leaders in this field with the development of a number of darunavir derivatives wherein the P2 domain has been changed to enhance drug efficacy.^13−15^ In this context, darunavir derivative **5** was designed with modifications to the P1, P2, and P2’domains to enhance interactions with the active site of the HIV-1 protease. The combined modifications led to enzyme inhibition that was markedly potent and yielded more insight into the nature of the binding properties of a series of related derivatives with the protease.

**Figure 2:**
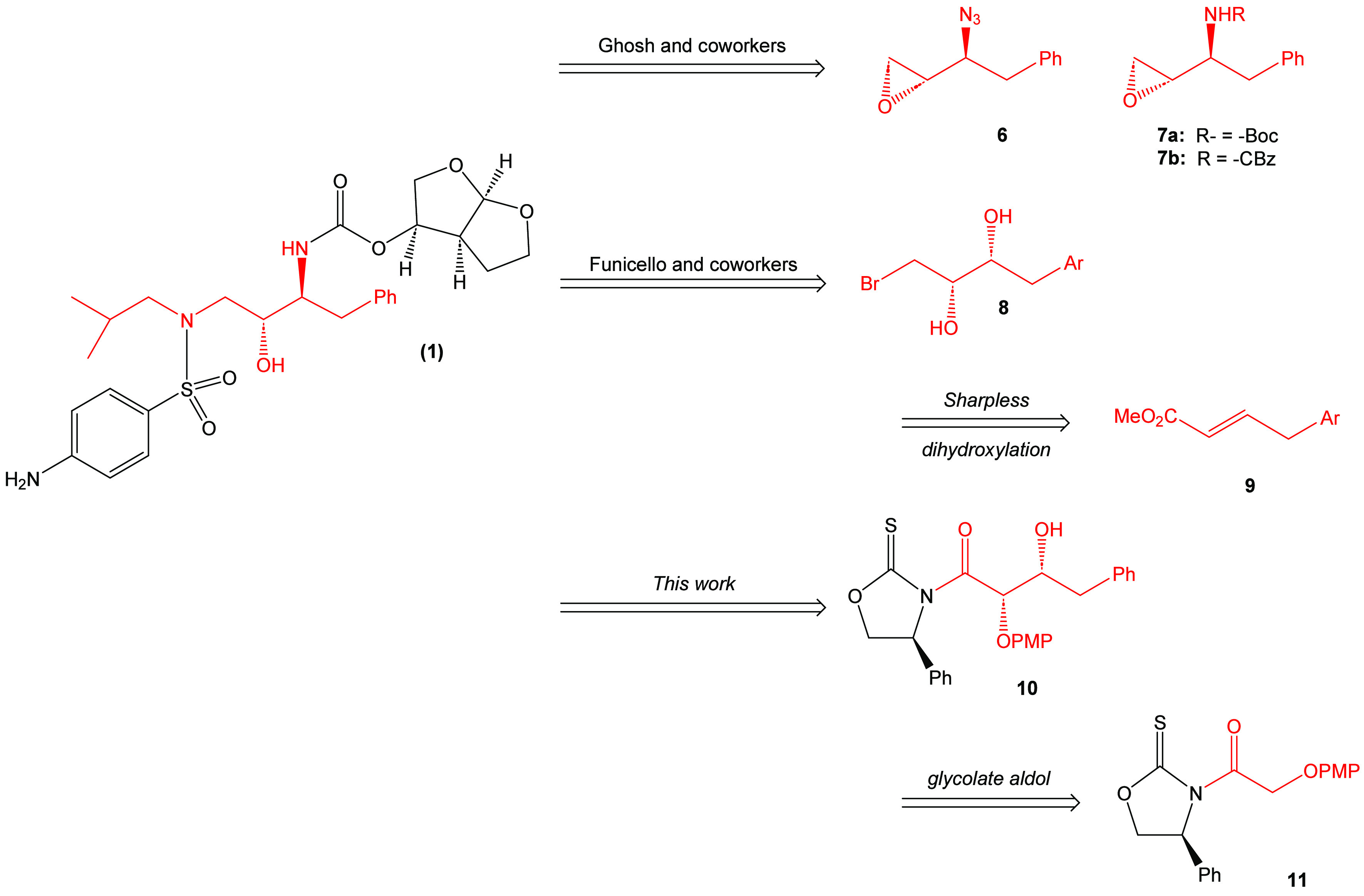
Synthetic pathways to darunavir (1).

The dominant synthetic pathway for the preparation of darunavir and many of its derivatives has involved the use of either β-epoxy azide **6**(15) or commercially available β-epoxy carbamates **7a,b** (a: *R* = -Boc;^7,11^ b: *R* = -CBz.^10^ An alternate pathway to the chiral structural framework of darunavir, which was developed by Funicello and coworkers,^9^ involves the preparation of a suitable vinyl ester **(9)** obtained from a ligand-free Suzuki-Miyaura coupling reaction (Figure 2). The Sharpless asymmetric dihydroxylation reaction is then used to create the requisite chiral centers necessary for the synthesis of darunavir.

**Scheme 1:**
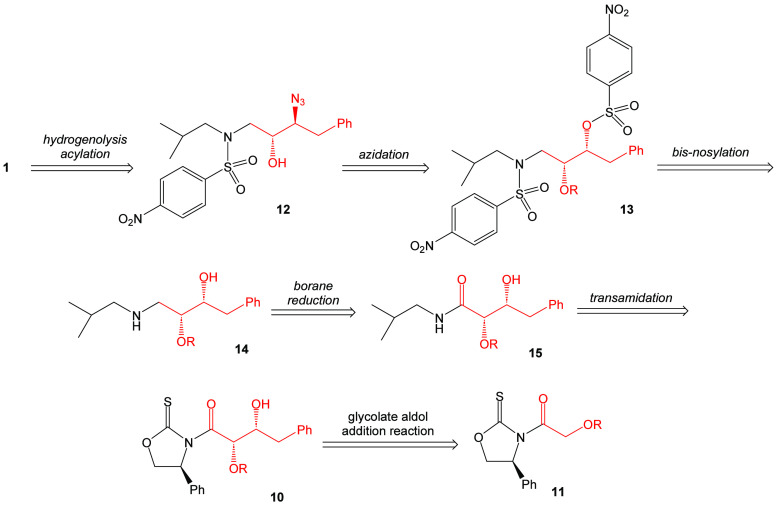
Retrosynthetic Analysis of Darunavir

There was an interest in determining the potential of using an asymmetric glycolate aldol addition pathway to achieve the synthesis of darunavir. Retrosynthetically, it is proposed that darunavir may be derived from the known intermediate β-azido alcohol **12** (Scheme 1). This material, in turn, may be derived from precursor **13** that possesses an alkoxy-protecting group. Intermediate **13** may be accessed by the bis-nosylation of intermediate **14**. It is proposed that the bis-nosylation process will introduce more convergence into the overall synthesis of the target molecule. Amino alcohol **14** can be envisioned to come from the reduction of amide **15**. This amide can be directly obtained by a process of transamidation with the aldol adduct **10** arising from the *syn*-stereoselective Crimmins glycolate aldol addition reaction with (*S*)-phenylglycinol-derived oxazolidine-2-thione **11**.

## Results and Discussion

**Scheme 2:**
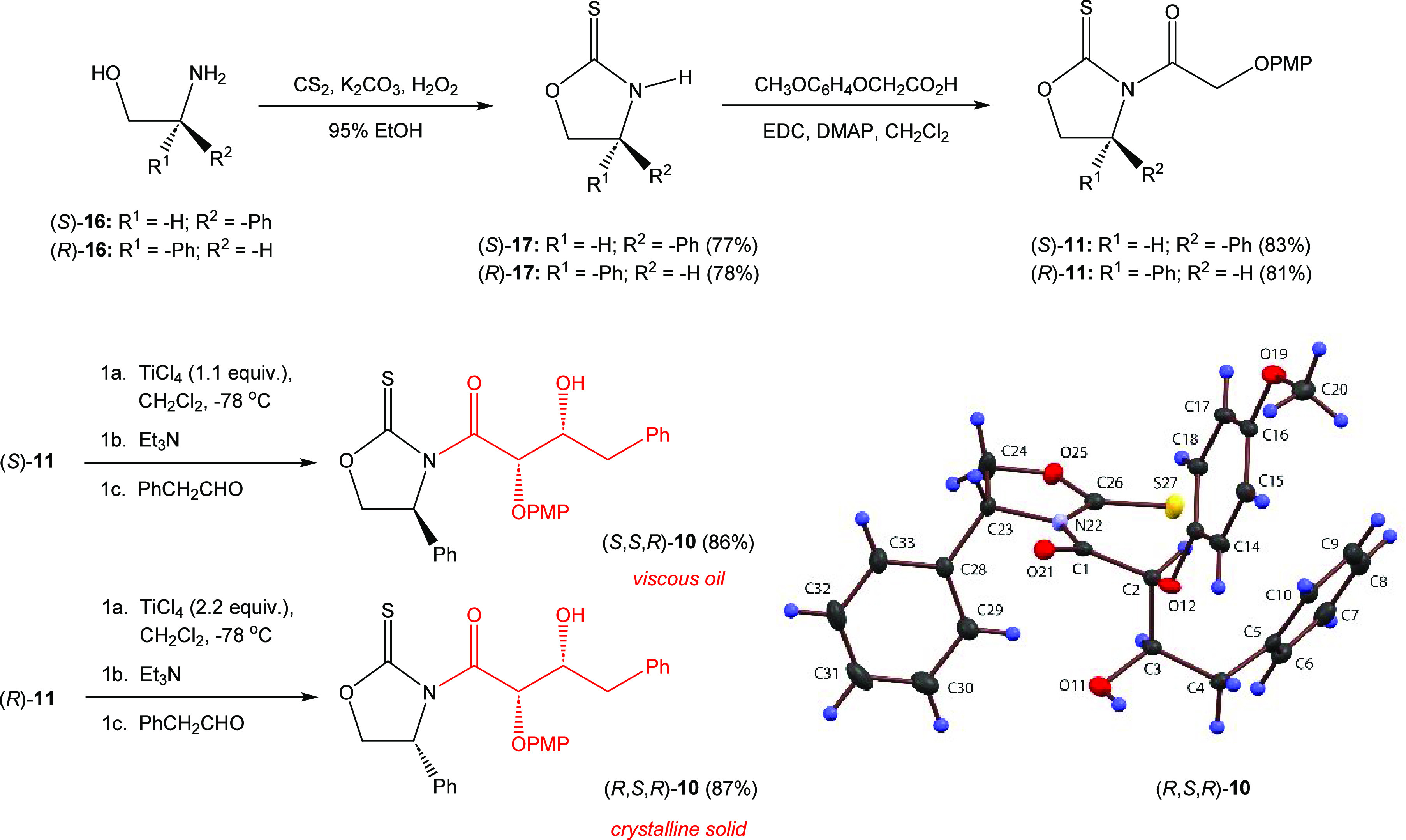
Synthesis of the Evans and Non-Evans *syn*-Glycolate Aldol Adducts **10**

Oxazolidine-2-thione [(*S*)-**16**] was prepared in 77% yield using the method of Wu and workers^16^ and subsequently acylated with *p*-methoxyphenoxyacetic acid in the presence of EDC and DMAP in dichloromethane to yield the *N*-(*p*-methoxyphenoxyacetyl)oxazolidine-2-thione (*S*)-**10** in 83% yield after recrystallization (Scheme 2). The reaction conditions for this process were optimized (slow addition of the carboxylic acid as the final reagent to the reaction mixture) to suppress the formation of the self-condensation byproduct that is proposed to arise from a putative Claisen condensation.^17^ The acylated thione (*S*)-**11** was then reacted with one equivalent of titanium tetrachloride and two equivalents of triethylamine in an asymmetric glycolate aldol addition reaction^18,19^ with phenylacetaldehyde to generate the Evan *syn*-adduct (*S*,*S*,*R*)-**10** in 86% yield and ≥95% diastereomeric purity after flash chromatography on silica gel.

The synthesis of the aldol adduct (*S*,*S*,*R*)-**10** was successful but required chromatographic purification. In order to optimize this process, an alternate route for obtaining the key aldol adduct side chain was considered.^20^ Thus, (*R*)-phenylglycinol [(*R*)-**16**] was cyclized using the Wu conditions to afford thione (*R*)-**17** in 78% yield after recrystallization. The thione was acylated as before with EDC and DMAP using the optimized conditions to yield (*R*)-**11** in 81% yield as a crystalline solid. This material was then reacted with two equivalents of titanium tetrachloride at −78 °C and enolized by the addition of two equivalents of triethylamine. Treatment of the titanium enolate with phenylacetaldehyde yielded the desired non-Evans *syn*-aldol adduct (*R*,*S*,*R*)-**10** in 87% yield (crude d.r. = 15:1) after recrystallization. The stereochemistry of the adduct was confirmed by single-crystal X-ray crystallography with the presence of the sulfur atom allowing clear assignment of absolute stereochemistry from the observed anomalous dispersion effects.^21^

**Scheme 3:**
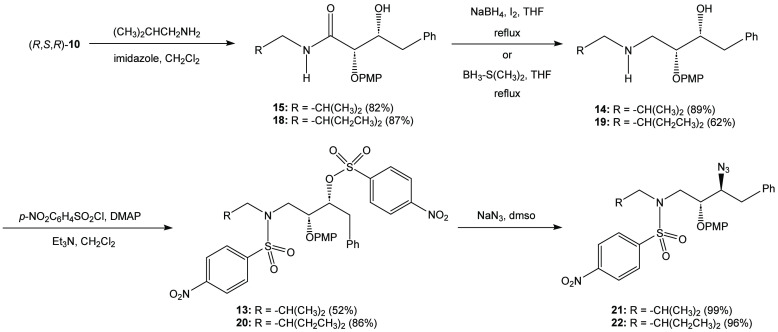
Synthesis of the γ-Azido-*N*-sulfonamide

With the synthesis of the key aldol reaction optimized, *syn*-aldol adduct (*R*,*S*,*R*)-**10** was reacted with isobutylamine and imidazole to afford the transamidation product, β-hydroxyamide **15**, in 82% after recrystallization (Scheme 3). In like fashion, 2-ethylbutylamine was employed in the transamidation process to yield amide **18** in 87% isolated yield. The introduction of this group introduces the 2-ethylbutylamine group as an eventual P1’ modification of darunavir. Subsequent reduction of amide **15** using the Meyers-Periasamy conditions (NaBH_4_/I_2_ in THF)^22,23^ yielded the β-aminoalcohol **14** in 89% yield after flash chromatography. Amide **18** was reduced using a commercially available borane-dimethylsulfide complex and yielded the β-amino alcohol **19** in 62% yield after chromatographic purification.

At this stage, β-amino alcohols **14** and **19** were treated with two equivalents of *p*-nitrobenzenesulfonyl chloride, triethylamine, and DMAP. It was anticipated that the bis-*p*-nosylation process would streamline the overall reaction. However, the reaction proved to have challenges, apparently due to the stereoelectronic factors governing the initial *N*-sulfonylation vs the *O*-sulfonylation. Ultimately, the reaction required 1.1 equivalents of catalytic DMAP to reach the complete formation of the bis-*p*-nosylation product. The target *O*-*p*-nitrobenzenesulfonyl-*N*-*p*-nitrobenzenesulfonamides **13** and **20** were obtained in 52% and 56% yield, respectively, as crystalline solids after recrystallization. Treatment of sulfonamides **13** and **20** with sodium azide in DMSO afforded a near quantitative conversion to the corresponding azides **21** and **22** in 99% and 96% recovered yield, respectively. The azides were not purified as the analysis of the NMR spectra suggested that the reactions were clean transformations.

**Scheme 4:**
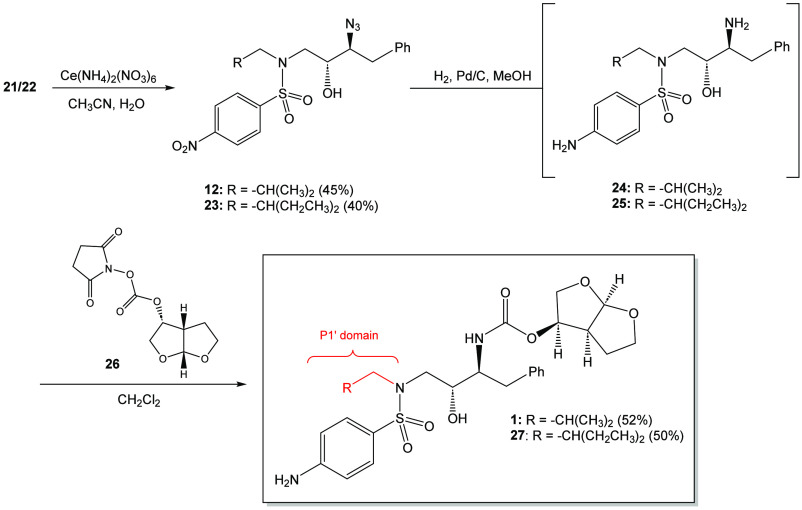
Synthesis of Darunavir **(1)** and *N*-2-Ethylbutyl Darunavir **(27)**

γ-Azidosulfonamides **21** and **22** were deprotected using ceric ammonium nitrate as a means of oxidatively removing the *p*-methoxyphenoxy group (Scheme 4).^24,25^ The isolated yield for the formation of β-azido alcohols **12** and **23** were 45% and 40%, respectively, after flash chromatography. Efforts to improve the reaction by adjusting the stoichiometry and changing the solvent and reaction temperature were not fruitful.^26^ The use of alternate deprotecting agent DDQ did not improve the yield of the reaction.

This process was followed by the hydrogenolysis of the β-azido alcohols **12** and **23** using hydrogen gas (balloon) to form β-amino alcohol **16***in situ*. The intermediates **24** and **25** were not isolated but were reacted with the bis(tetrahydrofuranyl) *N*-hydroxysuccinimidyl carbonate **(26)** to afford the target darunavir **(1)** and the related *N*-2-ethylbutyl darunavir derivative **(27)** in 52% and 50%, respectively, for the one-pot, two-reaction pathway.

**Scheme 5:**
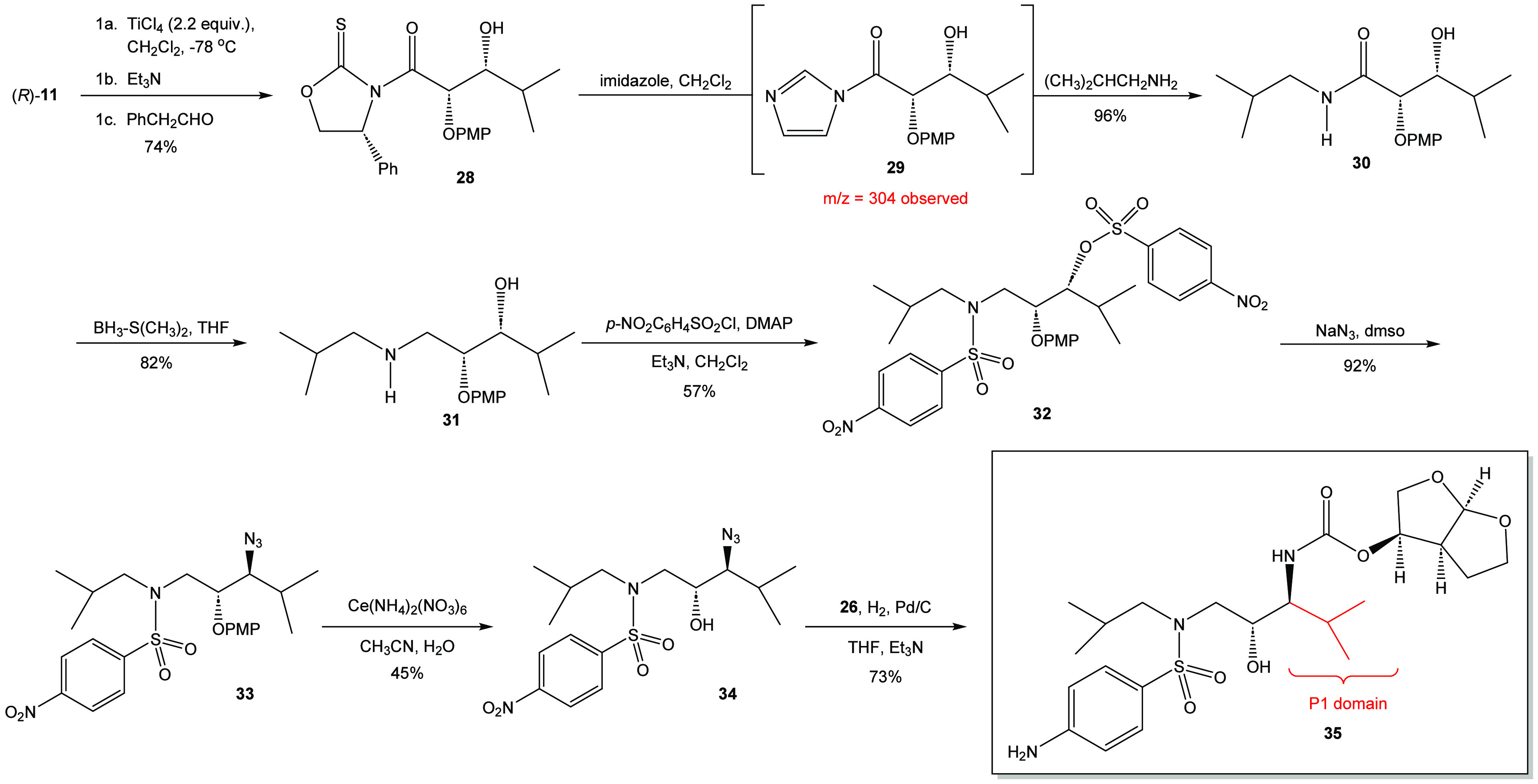
Synthesis of the P1-Isopropyl Derivative **35**

There was an interest in demonstrating that the overall synthetic pathway could be employed to create a P1 modification of the darunavir base structure (Scheme 5). In this regard, oxazolidine-2-thione (*R*)-**11** was reacted with titanium tetrachloride and triethylamine to form the titanium enolate that was subsequently alkylated with isobutyraldehyde to yield the aldol adduct **28** in 74% isolated yield in greater than 95% d.e. This material was reacted with isopropylamine and imidazole in dichloromethane. The crude reaction mixture yielded the desired product in combination with byproducts that appeared to be ring-opening products. To circumvent this issue, an alternate approach was taken in which the acyl imidazole was formed *in situ* and then the isopropylamine was added.^18b,27^ This process yielded the targeted *N*-isopropylamide **30** in 96% yield after recrystallization. The amide was reduced using borane-dimethylsulfide in THF to afford β-amino alcohol **31** in 82%. The β-amino alcohol was subsequently treated with an excess of *p*-nitrobenzenesulfonyl chloride and DMAP to afford the desired bis(*p*-nitrobenzenesulfonylated) adduct **32** in 57% yield after chromatographic purification. This material was then reacted with sodium azide to yield the corresponding azide **33** in high yield. Based on the quality of the crude ^1^H NMR spectrum of this material, it was moved forward to the process of deprotection via the use of ceric ammonium nitrate. As with the synthesis of darunavir and its *N*-2-ethylbutyl derivative, the ceric ammonium nitrate only proved to have limited efficiency in removing the *p*-methoxyphenoxy protecting group. The deprotection yielded β-azido alcohol **34** in 45% yield after purification by flash chromatography. Treatment of **34** with hydrogen gas in the presence of palladium on carbon and bis(tetrahydrofuranyl) *N*-hydroxysuccinimidyl carbonate **(26)** yielded the desired P1-isopropyl target **35** in 73% yield after chromatographic purification for the two-step process.

**Scheme 6:**
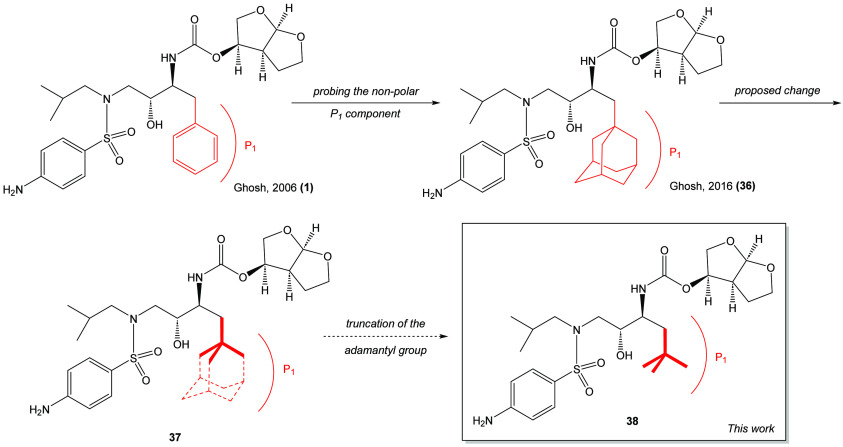
Origin of the P1-Neopentyl Darunavir Design

The synthesis of the P1-isopropyl derivative proved led to the pursuit of another P1-darunavir derivative based on the P1-adamantyl darunavir derivative prepared by Ghosh and coworkers (see Scheme 8 in ref (15)). It was originally proposed that the introduction of a hydrophobic and sterically demanding adamantyl group might provide further understanding of the protease inhibitor (PI)–protein interaction. It is proposed here that a contracted and truncated variant of the adamantyl system would also be a useful tool in probing the PI–protein interaction. To this end, the P1-adamantyl system was envisioned to be truncated down to the P1-neopentyl system illustrated in Scheme 6.

**Scheme 7:**
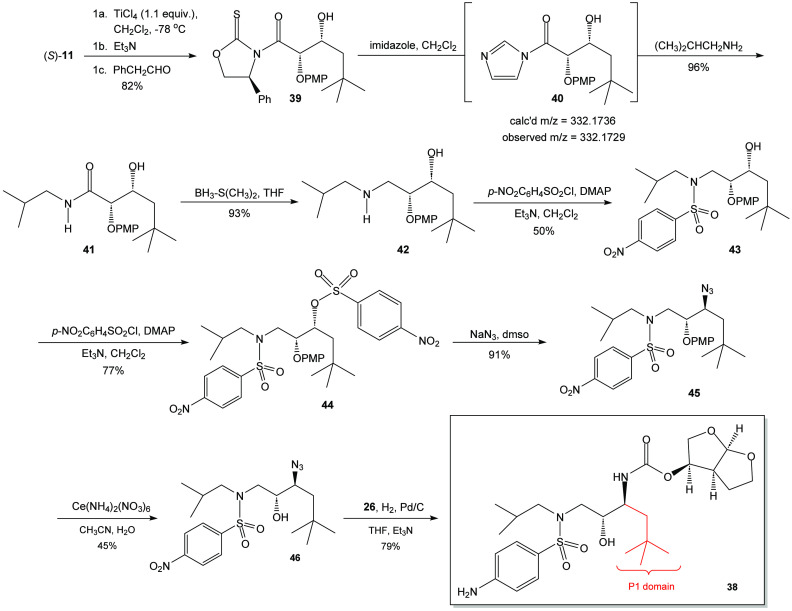
Synthesis of the Azido Neopentyl Darunavir Derivative

The pursuit of **38** was initiated with the titanium-mediated asymmetric aldol reaction of (*R*)-**11** with 3,3-dimethylbutyraldehyde (Scheme 7). The reaction proved to be incomplete, perhaps due to the steric demand of the neopentyl-type structure of the aldehyde. As a recourse, oxazolidine-2-thione (*S*)-**11** was employed in the asymmetric aldol addition reaction to afford aldol adduct **39** in 82% yield and greater than 95:5 d.r. This product was reacted with imidazole in the presence of dichloromethane, leading to the *in situ* formation of the acyl imidazole **40** that was tentatively identified by ESI-HRMS. Treatment of **40** with isobutylamine yielded amide **41** in 96% yield. As before, the amide was reduced to the corresponding β-amino alcohol **42** using a borane-dimethylsulfide complex. This process afforded a 93% yield after chromatographic purification. An attempt was made at the bis(sulfonylation) of β-amino alcohol **42** using two equivalents of the *p*-nitrobenzenesulfonyl chloride. Unfortunately, the product that was obtained in 50% yield was the monsulfonylated product **43**. The failure of the product to undergo bis(sulfonylation) was attributed to the steric hindrance imparted by the proximal neopentyl fragment. Thus, sulfonamide **43** was treated with an excess of *p*-nitrobenzenesulfonyl chloride to afford the desired bis(sulfonylated) product **44** in 77% yield. The reaction of **44** with sodium azide in DMSO led to the isolation of the azido neopentyl darunavir intermediate **45**. Deprotection of the *p*-methoxyphenoxy protecting group was accomplished by the use of a 4:1 acetonitrile/water solution of ceric ammonium nitrate. The product β-azido alcohol **46** was obtained in a 45% yield after chromatographic purification. Hydrogenation of β-azido alcohol **46** in the presence of Pd/C and carbonate **26** yielded neopentyl darunavir derivative **38** in 73% isolated yield after chromatography.

In conclusion, we have described an alternate preparative route to the HIV protease inhibitor darunavir using an asymmetric glycolate aldol addition reaction to form the key stereocenters. A strategy that involved the using enantiomeric oxazolidine-2-thione auxiliaries with opposing stereochemical outcomes in the asymmetric aldol addition reaction was successfully employed. A convergent strategy of introducing the key *p*-nitrobenzene sulfonamide and activating the chiral alcohol in the form of *p*-nitrobenzene sulfonate in the same chemical step was also employed. The overall synthesis of darunavir proved to be successful via the aldol addition pathway, but the potential for improving the current route remains open.

## Experimental Section

### Safety Considerations

It is noted that the formation of oxazolidine-2-thiones (*R*)-**17** and (*S*)-**17** involves an exothermic reduction/oxidation transformation that employs 30% hydrogen peroxide. It is recommended that a highly efficient water condenser is used for this reaction. The use of reagents such as titanium tetrachloride (1 M in CH_2_Cl_2_) and borane-dimethylsulfide complex should be handled with care using air-free techniques.

### General Methods^28,29^

Unless otherwise noted, all chemical reagents and solvents were purchased and used without further purification. All reactions were conducted under a nitrogen atmosphere in glassware that was flame-dried. Unless otherwise indicated, chemical reagents that served as starting materials, chemical reactants, and solvents were used as received from commercial vendors (e.g., MilliporeSigma, ThermoFisher Scientific, and Combi-Blocks, Inc.) without further purification. Melting points were recorded on a Mel-Temp apparatus and were uncorrected. Unless otherwise noted, all ^1^H and proton-decoupled ^13^C NMR spectra were collected in deuterated chloroform (CDCl_3_) using a Bruker Ultrashield Avance III NMR spectrometer operating at either 500 or 400 MHz (^1^H NMR) and 125 or 100 MHz (^13^C{^1^H} NMR), respectively. Chemical shifts were reported in parts per million (δ scale), and coupling constants (*J* values) were reported in Hertz (Hz). Tetramethylsilane (TMS) was used as an internal standard (δ = 0 ppm). Signals appearing in the ^1^H NMR spectra were recorded in abbreviated form: s = singlet; d = doublet; t = triplet; q = quartet; p = pentet; m = multiplet, dd = doublet of doublets. ^1^H-decoupled ^13^C NMR [^13^C{^1^H}] spectral signals were referenced and reported relative to the central peak of deuterated chloroform (CDCl_3_) taken as 77.0 ppm. Optical rotation data were collected at ambient laboratory temperatures (22–25 °C) on a JASCO *p*-1010 digital polarimeter operating at 589 nm and using a cylindrical 10 × 100 mm quartz cell. Infrared spectra were recorded using NaCl plates. IR values were reported in reciprocal centimeters (cm^–1^) and were measured either as a nujol mull or as a neat liquid film from an evaporated chloroform solution. Mass spectral data were collected on a ThermoScientific *Q*-Exactive electrospray ionization high-resolution mass spectrometer (ESI HRMS) equipped with an Orbitrap mass analyzer. Samples were prepared in concentrations of 5–25 ppm in high-performance liquid chromatographygrade methanol/water/formic acid (1:1:0.01).

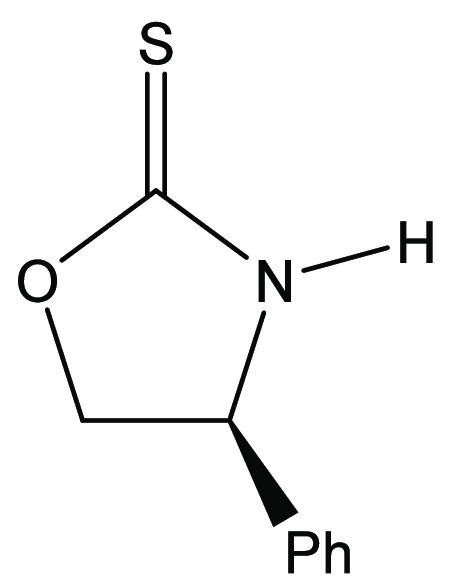



#### (4*S*)-4-Phenyl-1,3-oxazolidine-2-thione [(*S*)-**17**]^28^

To a flame-dried, nitrogen-purged 5 L round-bottom flask fitted with a Claisen adapter with a pressure-equalizing addition funnel and a tall condenser equipped with a stir bar were added l-phenylglycinol (13.7 g, 100 mmol, purchased from Combi-Blocks, Inc.), ethanol (100 mL), potassium carbonate (6.95 g, 50.0 mmol), and carbon disulfide (12 mL). The reaction was heated to 50 °C using a heating mantle controlled by a variable transformer, and a 30% (w/v) aqueous solution of hydrogen peroxide (17.0 mL, 167 mmol) was added dropwise. Upon complete addition of the hydrogen peroxide, the reaction was stirred for 15 min. The reaction is highly exothermic and must be carefully monitored during this time. It is important that the condenser is efficient and the system is open to the air. The reaction was cooled to ambient temperature and gravity-filtered into a 1 L flask to remove any solid material. The reaction flask was washed with ethyl acetate (3 × 30 mL), and the rinses were combined with the filtrate. The reaction solvent was then removed by rotary evaporation. The reaction was then reconstituted in ethyl acetate (200 mL). The organic layer was then washed with an aqueous solution of 1 M HCl (2 × 80 mL) and brine (80 mL). The organic layer was then dried (MgSO_4_) and filtered, and the solvents were removed by rotary evaporation. The crude solid product was then recrystallized with ethyl acetate and hexanes to afford the title compound as a light-yellow crystalline solid (13.7 g, 77 mmol, 77% yield). Melting point: 121–123 °C. [α]_D_ = +71.6 (*c* = 1.00, CHCl_3_). ^1^H NMR (500 MHz, CDCl_3_): δ 7.71 (broad singlet, 1H), 7.46–7.32 (m, 5H), 5.12 (dd, *J* = 8.9, 7.0 Hz, 1H), 5.00 (apparent triplet, *J* = 8.9 Hz, 1H), 4.49 (dd, *J* = 8.9, 7.0 Hz, 1H) ppm. ^13^C{^1^H} NMR (125 MHz, CDCl_3_): δ 189.7, 138.0, 129.2, 129.2, 126.3, 77.7, 60.2 ppm. This material was identical to that described by Wu and coworkers.^16^

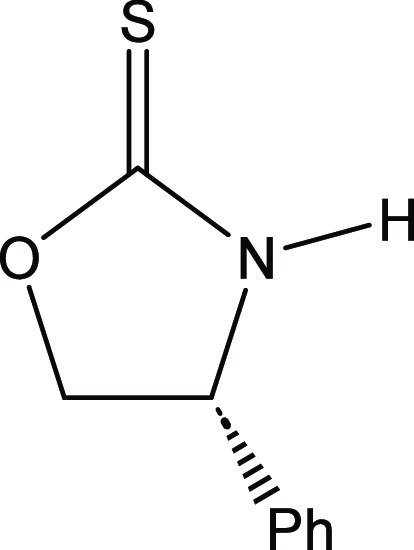



#### (4*R*)-4-Phenyl-1,3-oxazolidine-2-thione [(*R*)-**17**]^28^

d-Phenylglycinol (41.2 g, 300 mmol, purchased from Combi-Blocks, Inc.) was employed. The product was recrystallized and was recovered as a light-yellow crystalline solid (41.9 g, 234 mmol, 78% yield). Melting point: 121–122 °C. [α]_D_ = −70.4 (*c* = 0.27, CHCl_3_). ^1^H NMR (400 MHz, CDCl_3_): δ 7.51 (broad singlet, 1H), 7.45–7.30 (m, 5H), 5.11 (dd, *J* = 8.9, 6.9 Hz, 1H), 5.00 (apparent triplet, *J* = 9.2 Hz, 1H), 4.49 (dd, *J* = 8.9, 6.9 Hz, 1H) ppm. ^13^C{^1^H} NMR (100 MHz, CDCl_3_): δ 189.7, 138.0, 129.3, 129.1, 126.3, 77.7, 60.2 ppm. This material was identical to that described by Wu and coworkers.^16^

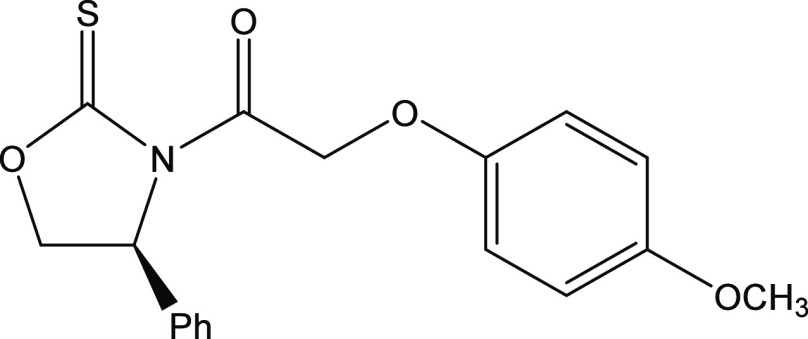



#### (4*S*)-3-[(*p*-Methoxyphenoxy)acetyl]-4-phenyl-1,3-oxazolidine-2-thione [(*S*)-**11**]^28^

To a flame-dried, nitrogen-purged 1000 mL round-bottom flask equipped with a stir bar were added oxazolidine-2-thione [(*S*)-**17**] (11.1 g, 61.4 mmol), dichloromethane (200 mL), EDC (12.9 g, 67.5 mmol), and DMAP (1.90 g, 15.4 mmol). Once these reagents had been added, *p*-methoxyphenoxy acetic acid (11.2 g, 61.4 mmol) was added portion-wise. The reaction mixture was then stirred overnight, and then, the contents of the round-bottom flask were transferred into a separatory funnel. The reaction mixture was then treated with an aqueous solution of 1 M HCl (60 mL). The organic layer was separated from the aqueous layer and then treated with an aqueous solution of 1 M NaOH (2 × 60 mL). The organic layer was separated and finally washed with brine (60 mL). The organic layer was collected, dried (MgSO_4_), and gravity-filtered. The solvent was removed by rotary evaporation, and the crude product was recrystallized from ethyl acetate and hexanes to afford compound (*S*)-**11** as a white crystalline solid (17.5 g, 50.9 mmol, 83%). Mp: 108–109 °C. [α]_D_ = +83.2 (*c* = 1.00, CHCl_3_,). ^1^H NMR (400 MHz, CDCl_3_): δ 7.40–7.30 (m, 5H), 6.82–6.76 (m, 4H), 5.73 (dd, *J* = 9.0, 3.3 Hz, 1H), 5.57 (d, *J* = 17.7 Hz, 1H), 5.45 (d, *J* = 17.7 Hz, 1H), 4.90 (apparent triplet, *J* = 9.0 Hz, 1H), 4.58 (dd, *J* = 9.0, 3.3 Hz, 1H), 3.74 (s, 3H) ppm. ^13^C{^1^H} NMR (100 MHz, CDCl_3_): δ 185.0, 168.9, 154.5, 151.8, 138.2, 129.3, 129.0, 126.3, 115.9, 114.7, 75.4, 70.2, 62.0, 55.7 ppm. IR (CHCl_3_): 1724, 1211, 821, 780 cm^–1^. HRMS (ESI-TOF) *m*/*z*: [M + Na]^+^ Calcd for C_18_H_17_NNaO_4_S366.0770; Found, 366.0777. The material has been previously prepared.^18a^

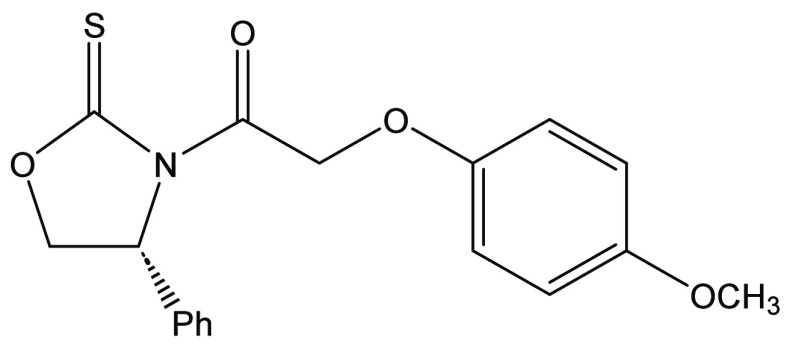



#### (4*R*)-3-[(*p*-Methoxyphenoxy)acetyl]-4-phenyl-1,3-oxazolidine-2-thione [(*R*)-**11**]^28^

Using the above procedure, oxazolidine-2-thione (*R*)**−17** (17.9 g, 100 mmol) was employed as the substrate. The crude solid product was recrystallized using ethyl acetate and hexanes to afford the product as a crystalline solid (27.8 g, 81.0 mmol, 81%). Mp: 109–110 °C. [α]_D_ = −73.9 (*c* = 1.04, CHCl_3_). ^1^H NMR (400 MHz, CDCl_3_): δ 7.41–7.30 (m, 5H), 6.82–6.76 (m, 4H), 5.73 (dd, *J* = 9.0, 3.2 Hz, 1H), 5.57 (d, *J* = 17.7 Hz, 1H), 5.45 (d, *J* = 17.7 Hz, 1H), 4.90 (apparent triplet, *J* = 9.0 Hz, 1H), 4.58 (dd, *J* = 9.0, 3.2 Hz, 1H), 3.74 (s, 3H) ppm. ^13^C{^1^H} NMR (100 MHz, CDCl_3_): δ 185.0, 169.0, 154.5, 151.8, 138.2, 129.3, 129.1, 126.3, 115.9, 114.7, 75.4, 70.2, 62.1, 55.7 ppm. IR (CHCl_3_): 1721, 1206, 826, 786 cm^–1^. HRMS (ESI-TOF) *m*/*z*: [M + Na]^+^ Calcd for C_18_H_17_NNaO_4_S 366.0770; Found, 366.0776.

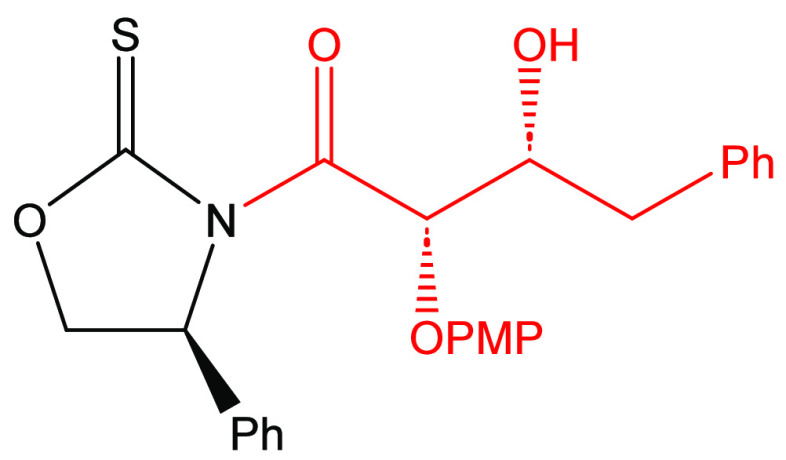



#### (4*S*)-3-[(2*S*’,3*R*’)-3-Hydroxy-2-(*p*-methoxyphenoxy)-4-phenylbutanoyl]-4-phenyl-1,3-oxazolidine-2-thione[(*S*,*S*,*R)*-**10**]^28^

To a flame-dried, nitrogen-purged 5 L round-bottom flask equipped with a large stir bar were added acylated thione [(*S*)-**11**)] (10.0 g, 29.1 mmol) and anhydrous dichloromethane (1 L). The reaction vessel was chilled to −78 °C with a dry ice/ethanol bath, and titanium tetrachloride (1 M in CH_2_Cl_2_, 32.0 mL, 32.0 mmol) was added dropwise by syringe. This solution was stirred for 30 min, at which point triethylamine (8.90 mL, 64 mmol) was added by syringe. The color of the solution transitioned from an amber color to that of deep purple upon the addition of the triethylamine. This solution was stirred for 60 min at −78 °C, and freshly distilled phenylacetaldehyde (7.2 mL, 64 mmol) was added to the reaction vessel by syringe. The reaction mixture was stirred for an additional 4 h, after which time the dry ice bath was removed, and brine (200 mL) was added dropwise to quench the reaction. The reaction mixture was allowed to warm up to room temperature with vigorous stirring, transferred to a separatory funnel, and extracted with an aqueous solution of 1 M HCl (2 × 100 mL). The organic layer was separated and subsequently washed with brine (100 mL), dried (MgSO_4_), and filtered. The solvent was then removed by rotary evaporation to yield the crude reaction product as a viscous oil. The product was purified by flash chromatography on silica gel using a solvent gradient [100% hexanes; 85:15, hexanes:ethyl acetate; 60:40, hexanes:ethyl acetate] to achieve the isolation of the pure product (11.54 g, 24.89 mmol, 86% yield). ^1^H NMR (500 MHz, CDCl_3_): δ 7.30–7.14 (m, 10 H), 6.76 (d, *J* = 2.0 Hz, 1H), 6.69–6.63 (m, 4H), 5.58 (dd, *J* = 8.0, 2.3 Hz, 1H), 4.67 (dd, *J* = 9.0, 8.0 Hz), 4.55 (td, *J* = 7.5, 2.0 Hz, 1H), 4.36 (dd, *J* = 9.0, 2.3 Hz, 1H), 3.71 (s, 3H), 3.11–3.04 (m, 2H). ^13^C{^1^H} NMR (125 MHz, CDCl_3_): δ 185.2, 169.9, 154.6, 151.0, 138.3, 137.2, 129.6, 129.2, 128.8, 128.5, 126.8, 125.7, 116.1, 114.7, 77.3 (obscured by the CDCl_3_ signal), 74.7, 73.0, 62.7, 55.7, 40.6 ppm. The material has been previously prepared.^18a^

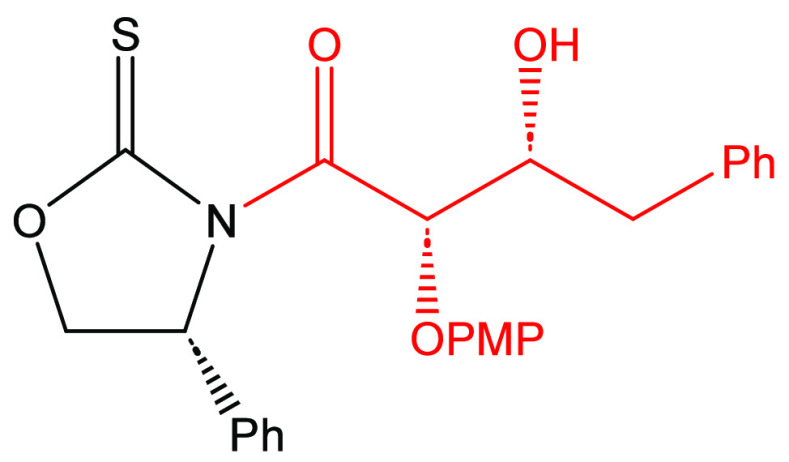



#### (4*R*)-3-[(2*S*’,3*R*’)-3-hydroxy-2-(*p*-methoxyphenoxy)-4-phenylbutanoyl]-4-phenyl-1,3-oxazolidine-2-thione[(*R*,*S*,*R*)-**10**]^28^

To a flame-dried, nitrogen-purged 5 L round-bottom flask equipped with a large stir bar were added acylated thione (*R*)-**11** (10.0 g, 29.1 mmol) and anhydrous dichloromethane (1 L). The reaction vessel was chilled to −78 °C with a dry ice/ethanol bath, and titanium tetrachloride (1 M in CH_2_Cl_2_, 64 mL, 64 mmol) was added dropwise by syringe. This solution was stirred for 30 min, at which point triethylamine (8.90 mL, 64 mmol) was added by syringe. The color of the solution transitioned from an amber color to that of deep purple upon the addition of the triethylamine. This solution was stirred for 60 min at −78 °C, and freshly distilled phenylacetaldehyde (7.2 mL, 64 mmol) was added to the reaction vessel by syringe. The reaction mixture was stirred for an additional 4 h, after which time the dry ice bath was removed, and brine (200 mL) was added dropwise to quench the reaction. The reaction contents were allowed to gradually warm up to room temperature with vigorous stirring, transferred to a separatory funnel, and then extracted twice with an aqueous solution of 1 M HCl (100 mL). The organic layer was separated from the aqueous layer and subsequently washed with brine (100 mL), dried (MgSO_4_), and filtered. The solvent was then removed by rotary evaporation to yield the crude reaction product as a tan solid, which was then recrystallized using hexanes and ethyl acetate to afford the pure product as a white, crystalline solid (11.73 g, 25.45 mmol, 87% yield). Mp: 184–185 °C. [α]_D_ = −105.8 (*c* = 1.10, CHCl_3_). ^1^H NMR (400 MHz, CDCl_3_): δ 7.38–7.17 (m, 11H), 6.86–6.80 (m, 4H), 5.70 (dd, *J* = 9.2, 5.9 Hz, 1H), 4.85 (apparent triplet, *J* = 9.2 Hz, 1H), 4.55–4.51 (m, 1H), 4.48 (dd, *J* = 9.2, 5.9 Hz, 1H), 3.76 (s, 3H), 3.06–2.94 (m, 2H) ppm. ^13^C{^1^H} NMR (100 MHz, CDCl_3_): δ 185.4, 170.4, 154.7, 151.1, 137.3, 137.0, 129.7, 129.2, 129.1, 128.5, 126.7, 126.5, 116.3, 114.9, 77.5, 74.7, 73.2, 62.6, 55.7, 40.4 ppm. IR (CHCl_3_)_:_ 3424, 1717, 1216, 757 cm^–1^. HRMS (ESI-TOF) *m*/*z*: [M + Na]^+^ Calcd for C_26_H_25_NNaO_5_S 486.1346; Found, 486.1349. Suitable crystals for single-crystal X-ray diffraction analyses were grown by vapor diffusion of pentane into a dichloromethane solution of (*R*,*S*,*R*)-**10**.

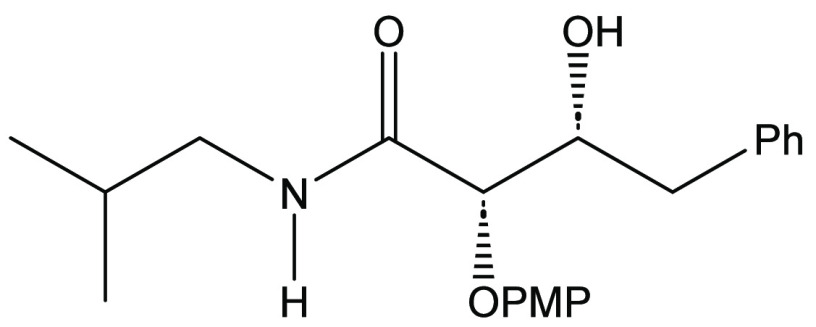



#### (2*S*,3*R*)-3-Hydroxy-*N*-isobutyl-2-(*p*-methoxyphenoxy)-4-phenylbutanamide (**15**)^28^

To a flame-dried, nitrogen-purged 500 mL round-bottom flask equipped with a stir bar were added the aldol adduct (*R*,*S*,*R*-**10**) (10.0 g, 21.6 mmol), dichloromethane (70 mL), and imidazole (4.40 g, 64.7 mmol). Once the reaction had stirred for 60 min, isobutylamine (4.30 mL, 43.0 mmol) was added by syringe, and the reaction was stirred overnight. The reaction was diluted with dichloromethane (80 mL), transferred to a separatory funnel, and treated with an aqueous solution of 2 M NaOH (2 × 40 mL). The layers were separated, and the organic layer was treated with an aqueous solution of 1 M HCl (2 × 40 mL). The organic layer was then washed with brine (40 mL), dried (MgSO_4_), and gravity-filtered. The solvent was then removed by rotary evaporation to yield the crude reaction product, which was then recrystallized using hexanes and ethyl acetate to afford the pure product as a white solid (6.33 g, 17.7 mmol, 82% yield). Multiple reactions were conducted to build up material for the following reaction. Mp: 102–103 °C. [α]_D_ = −6.51 (*c* = 1.04, CHCl_3_). ^1^H NMR (500 MHz, CDCl_3_): δ 7.27–7.16 (m, 5H), 6.88 (d, *J* = 9.3 Hz, 2H), 6.84 (d, *J* = 9.3 Hz, 2H), 6.60 (broadened triplet, 1H), 4.49 (d, *J* = 3.0 Hz, 1H), 4.30 (broad singlet, 1H), 3.78 (s, 3H), 3.17–3.09 (m, 2H), 2.97 (dd, *J* = 13.8, 5.2 Hz, 1H), 2.90 (dd, *J* = 13.8, 8.4 Hz, 1H), 2.77 (d, *J* = 7.5 Hz, 1H), 1.80–1.70 (m, 1H), 0.86 (d, *J* = 6.7 Hz, 3H), 0.85 (d, *J* = 6.7 Hz, 3H) ppm. ^13^C{^1^H} NMR (125 MHz, CDCl_3_): δ 170.3, 155.1, 151.3, 137.7, 129.4, 128.5, 126.6, 116.6, 115.0, 80.7, 73.0, 55.7, 46.4, 39.6, 28.5, 20.0, 19.9 ppm. IR (nujol): 3310, 1653, 1215 cm^–1^. HRMS (ESI-TOF) *m*/*z*: [M + Na]^+^ Calcd for C_21_H_27_NO_4_Na 380.1832; Found, 380.1840.

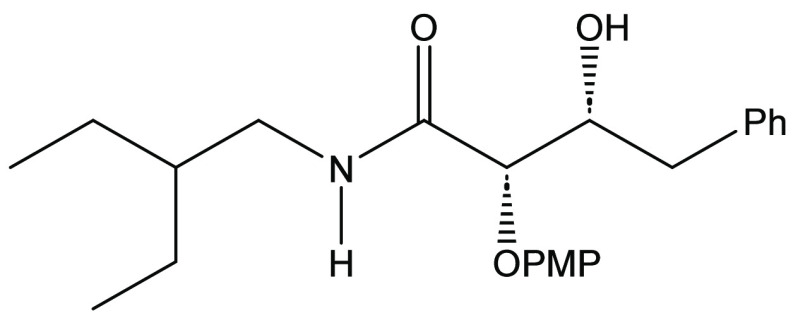



#### (2*S*,3*R*)-*N*-(2-Ethylbutyl)-3-hydroxy-2-(4-methoxyphenoxy)-4-phenylbutanamide (**18**)^29^

To a stirred solution of **10** (7.00 g, 15.1 mmol) in dichloromethane (151 mL) was added imidazole (3.08 g, 45.2 mmol), and after 1 h, 2-ethylbutylamine (2.20 mL, 16.1 mmol) was added to the mixture. The reaction mixture was stirred overnight, diluted with dichloromethane, washed with an aqueous solution of 2 M NaOH (30 mL) and brine (30 mL), and dried over MgSO_4_, and the solvent was removed by rotary evaporation. The crude residue was purified by flash column chromatography (3% methanol in dichloromethane) to afford **18** (5.07 g, 13.1 mmol, 87% yield) as a clear viscous oil that crystallizes with time. [α]_D_ = −9.3 (*c* = 0.562, CHCl_3_,). ^1^H NMR (500 MHz, CDCl_3_): δ 7.27–7.17 (m, 5H), 6.88 (d, *J* = 9.3 Hz, 2H), 6.83 (d, *J* = 9.3 Hz, 2H), 6.50 (m, 1H), 4.49 (d, *J* = 3.0 Hz, 1H), 4.31–4.27 (m, 1H), 3.77 (s, 3H), 3.25 (t, *J* = 6.3 Hz, 2H), 2.97 (dd, *J* = 13.8, 5.0 Hz, 1H), 2.90 (dd, *J* = 13.8, 8.7 Hz, 1H), 1.39–1.32 (m, 1H), 1.29–1.16 (m, 4H), 0.84 (t, *J* = 7.4 Hz, 6H), ppm. ^13^C{^1^H} NMR (100 MHz, CDCl_3_): δ 170.3, 155.1, 151.3, 137.7, 129.4, 128.5, 126.6, 116.6, 115.0, 80.6, 73.0, 55.7, 41.5, 40.9, 39.5, 23.6, 10.9, 10.8 ppm. IR (CDCl_3_): 3421, 3355, 1655, 1506, 1223, 1037, 826 cm^–1^. HRMS (ESI-TOF) *m*/*z*: [M + Na]^+^ Calcd for C_23_H_38_N_3_O_7_SNa 386.2326; Found, 386.2328.

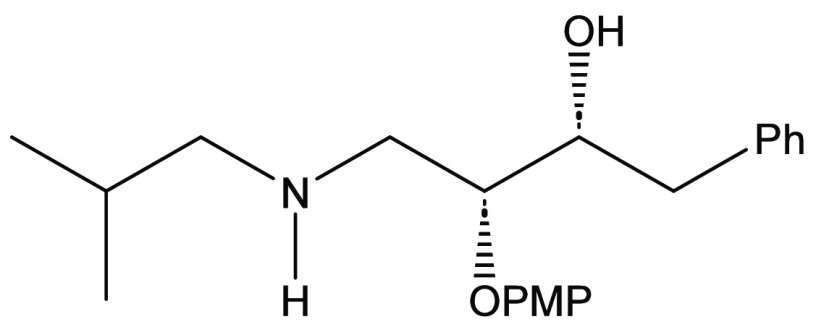



#### (2*R*,3*R*)-2-Hydroxy-4-(isobutylamino)-3-(*p*-methoxyphenoxy)-1-phenylbutane (**14**)^28^

To a flame-dried, nitrogen-purged, 1000 mL round-bottom flask fitted with a Claisen adapter equipped with a condenser and pressure-equalizing addition funnel were added β-hydroxyamide **(15)** (13.3 g, 37.1 mmol) and THF (180 mL). Sodium borohydride (3.37 g, 89.0 mmol) was added incrementally to the flask and stirring was initiated. A solution of iodine (10.4 g, 40.8 mmol) in THF (70 mL) was prepared and carefully added to the addition funnel, whereafter dropwise addition of this solution into the reaction flask was completed over the course of 15 min. The reaction was heated to reflux using a heating mantle controlled by a variable transformer and was subsequently stirred for 16 h. At the end of the reaction time, the reaction was cooled to 0 °C and quenched by the dropwise addition of methanol (50 mL) via the addition funnel. The mixture was concentrated under reduced pressure and then extracted with EtOAc (150 mL) and an aqueous solution of 1 M NaOH (3 × 50 mL). The organic layer was washed with brine (50 mL), dried over magnesium sulfate, and gravity-filtered. The solvent was then removed by rotary evaporation to afford the corresponding amino alcohol as a colorless oil that was used in the next step without further purification (11.4 g, 33.1 mmol, 89% yield). [α]_D_ = −52.5 (*c* = 1.17, CDCl_3_). ^1^H NMR (500 MHz, CDCl_3_): δ 7.26–7.14 (m, 5H), 6.88–6.82 (m, 4H), 4.19 (td, *J* = 7.0, 1.9 Hz, 1H), 4.08–4.06 (m, 1H), 3.78 (s, 3H), 3.27 (dd, *J* = 12.7, 4.3 Hz, 1H), 3.03–2.93 (m, 2H), 2.73 (dd, *J* = 12.7 Hz, 2.4 Hz, 1H), 2.42 (dd, *J* = 11.5, 6.5 Hz, 1H), 2.32 (dd, *J* = 11.5, 7.0 Hz, 1H), 1.73–1.65 (m, 1H), 0.89 (d, *J* = 6.7 Hz, 3H), 0.88 (d, *J* = 6.7 Hz, 3H) ppm. ^13^C{^1^H} NMR (100 MHz, CDCl_3_): δ 154.4, 151.7, 138.7, 129.5, 128.4, 126.2, 117.3, 114.9, 76.6, 75.9, 58.0, 55.7, 50.6, 39.8, 28.2, 20.6, 20.5 ppm. IR (CHCl_3_): 3168, 1218 cm^–1^. HRMS (ESI-TOF) *m*/*z*: [M + H]^+^ Calcd for C_21_H_30_NO_3_ 344.2220; Found, 344.2220.

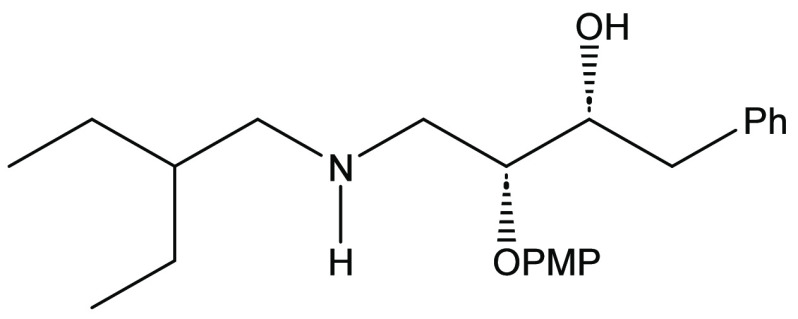



#### (2*R*,3*R*)-4-((2-Ethylbutyl)amino)-3-(*p*-methoxyphenoxy)-1-phenyl-2-butanol (**19**)^29^

To a flame-dried, nitrogen-purged 2 L round-bottom flask were added β-hydroxyamide **18** (4.23 g, 11.0 mmol) and THF (275 mL). To this mixture was added a borane-dimethylsulfide complex (3.10 mL). The reaction was heated to reflux 18 h using a heating mantle controlled by a variable transformer. The reaction mixture was cooled to ambient temperature and was then cooled with an ice bath. The reaction was quenched with 20 mL of methanol and allowed to stir for 30 min in an ice bath. The quenched reaction was diluted with diethyl ether (100 mL) and then washed with an aqueous solution of 1 M NaOH (2 × 30 mL). The organic layer was washed one more time with brine, dried over MgSO_4_, and concentrated under reduced pressure. The residue was purified by flash column chromatography on silica gel (7:3, diethyl ether: hexanes) to afford **19** (2.52 g, 6.82 mmol, 62% yield) as a colorless viscous oil. ^1^H NMR (500 MHz, CDCl_3_): δ 7.26–7.15 (m, 5H), 6.87 (d, *J* = 9.3 Hz, 2H), 6.83 (d, *J* = 9.3 Hz, 2H), 4.19 (td, *J* = 7.1, 2.0 Hz, 1H), 4.06 (p, 1H), 3.78 (s, 3H), 3.25 (dd, *J* = 12.7, 4.3 Hz, 1H), 2.97 (m, 2H), 2.71 (dd, *J* = 12.7, 2.3 Hz, 1H), 2.48 (dd, *J* = 11.6, 4.8 Hz, 1H), 2.42 (dd, *J* = 11.6, 5.4 Hz, 1H), 1.30 (m, 5H), 0.83 (m, 6H) ppm. ^13^C{^1^H} NMR (100 MHz, CDCl_3_): δ 154.4, 151.6, 138.6, 129.5, 128.4, 126.2, 117.3, 114.8, 76.9, 75.6, 55.7, 52.8, 51.0, 40.8, 39.7, 24.0, 23.9, 10.9, 10.8 ppm. IR CDCl_3_: 3321, 3036, 1226, 1107, 1040, 827 cm^–1^. HRMS (ESI-TOF) *m*/*z*: [M + Na]^+^ Calcd for C_23_H_34_NO_3_SNa 372.2533; Found, 372.2536.

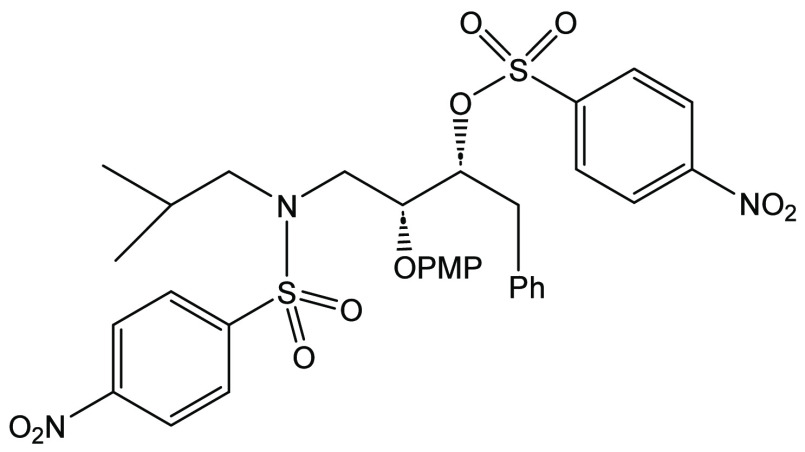



#### (2*R*,3*R*)-4-(*N*-(2-Isobutyl)-*p*-nitrophenylsulfonamido)-3-(*p*-methoxyphenoxy)-1-phenyl-2-*p*-nitrobenzenesulfonylbutane (**13**)^28^

To a flame-dried, nitrogen-purged 250 mL round-bottom flask equipped with a stir bar were added β-amino alcohol **14** (6.74 g, 19.6 mmol) and dichloromethane (64 mL). Once the substrate had fully dissolved, *p*-nitrobenzenesulfonyl chloride (10.9 g, 40.3 mmol), DMAP (2.64 g, 21.6 mmol), and triethylamine (11 mL, 79 mmol) were added. The reaction was vigorously stirred overnight, and the reaction contents were diluted with dichloromethane (80 mL), transferred to a separatory funnel, and washed with an aqueous solution of 1 M HCl (2 × 50 mL). The organic layer was then washed with brine (50 mL), dried (MgSO_4_), gravity-filtered, and concentrated under reduced pressure (7.30 g, 10.2 mmol, 52% yield). Mp: 161–162 °C. [α]_D_ = +113.1 (*c* = 1.02, CHCl_3_). ^1^H NMR (500 MHz, CDCl_3_): δ 8.19 (d, *J* = 8.9 Hz, 2H), 7.98 (d, *J* = 8.9 Hz, 2H), 7.96 (d, *J* = 8.9 Hz, 2H), 7.50 (d, *J* = 8.9 Hz, 2H), 7.06 (t, *J* = 7.4 Hz, 1H), 6.96 (t, *J* = 7.4 Hz, 2H), 6.77 (d, *J* = 9.2 Hz, 2H), 6.73 (d, *J* = 7.4 Hz, 2H), 6.69 (d, *J* = 9.2 Hz, 2H), 4.80–4.77 (m, 1H), 4.68–4.65 (m, 1H), 3.87 (d, *J* = 15.5 Hz, 1H), 3.78 (s, 3H), 3.62 (dd, *J* = 15.5, 9.4 Hz, 1H), 3.28 (dd, *J* = 13.6, 8.6 Hz, 1H), 3.11 (dd, *J* = 13.6, 6.6 Hz, 1H), 3.05 (dd, *J* = 14.6, 2.2 Hz, 1H), 2.64 (dd, *J* = 14.6, 10.8 Hz, 1H), 2.15–2.06 (m, 1H), 0.96 (d, *J* = 6.7 Hz, 3H), 0.95 (d, *J* = 6.7 Hz, 3H) ppm. ^13^C{^1^H} NMR (100 MHz, CDCl_3_): δ 155.0, 150.4, 149.8, 149.7, 146.0, 140.5, 135.6, 129.0, 128.7, 128.6, 128.3, 126.9, 124.3, 124.0, 116.3, 114.9, 82.5, 75.6, 56.2, 55.7, 46.7, 34.1, 26.3, 19.9, 19.8 ppm. IR (CHCl_3_): 1531, 1350 cm^–1^. HRMS (ESI-TOF) *m*/*z*: [M + Na]^+^ Calcd for C_33_H_35_N_3_NaO_11_S_2_ 736.1605; Found, 736.1606.

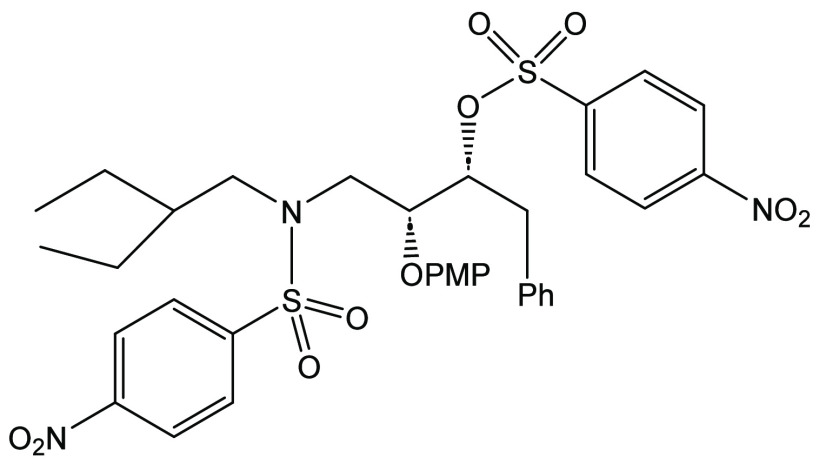



#### (2*R*,3*R*)-4-(*N*-(2-Ethylbutyl)-*p*-nitrophenylsulfonamido)-3-(*p*-methoxyphenoxy)-1-phenyl-2-*p*-nitrobenzenesulfonylbutane (**20**)^29^

To a stirred solution of β-amino alcohol **19** (2.16 g, 5.80 mmol) dissolved in dichloromethane (19 mL) were added *p*-nitrobenzenesulfonyl chloride (3.22 g, 14.5 mmol) and DMAP (0.782 g, 6.40 mmol). Lastly, triethylamine (3.20 mL, 23.1 mmol) was then added dropwise to the reaction mixture via an addition funnel. The reaction mixture was stirred overnight at room temperature. After completion, the reaction mixture was diluted with dichloromethane and washed with aqueous solution of 1 M HCl (2 × 30 mL). The organic layer was treated once with brine, dried over MgSO_4_, and concentrated under reduced pressure to afford a crude yellow viscous oil. The crude viscous oil was crystallized from diethyl ether and hexanes to yield **20** (3.72 g, 4.99 mmol, 86% yield) as yellow crystals. Mp: 152.3–153.6 °C. ^1^H NMR (500 MHz, CDCl_3_): δ 8.22 (d, *J* = 9.0 Hz, 2H), 7.99 (d, *J* = 9.0 Hz, 2H), 7.95 (d, *J* = 9.0 Hz, 2H), 7.50 (d, *J* = 9.0 Hz, 2H), 7.05 (t, *J* = 7.3 Hz, 1H), 6.96 (t, *J* = 7.8 Hz, 2H), 6.77 (d, *J* = 9.2 Hz, 2H), 6.72 (d, *J* = 8.5 Hz, 2H), 6.69 (d, *J* = 9.2 Hz, 2H), 4.82 (dq, *J* = 9.6, 4.1, 1.5 Hz, 1H),.82 (dq, *J* = 10.8, 4.1, 2.3 Hz, 1H), 3.83 (dd, *J* = 15.5, 1.2 Hz, 1H), 3.79 (s, 3H), 3.61 (dd, *J* = 15.4, 9.5 Hz, 1H), 3.40 (dd, *J* = 13.6, 8.5 Hz, 1H), 3.13 (dd, *J* = 13.6, 6.2 Hz, 1H), 3.05 (dd, *J* = 14.6, 2.2 Hz, 1H), 2.64 (dd, *J* = 14.6, 10.8 Hz, 1H), 1.77–1.70 (m, 2H), 1.48–1.27 (m, 4H), 0.91 (t, *J* = 7.4 Hz, 3H), 0.85 (t, *J* = 7.4 Hz, 3H) ppm. ^13^C{^1^H} NMR (100 MHz, CDCl_3_): δ 155.0, 150.3, 149.72, 149.70, 145.9, 140.5, 135.6, 129.0, 128.7, 128.6, 128.3, 126.9, 124.3, 124.0, 116.3, 114.9, 82.5, 75.4, 55.7, 52.4, 46.5, 37.9, 34.2, 22.9, 22.6, 10.6, 10.2 ppm. IR (CDCl_3_): 3108, 1531, 1506, 1350, 855 cm^–1^. HRMS (ESI-TOF) *m*/*z*: [M + Na]^+^ Calcd for C_35_H_40_N_3_NaO_11_S_2_ 742.2099; Found, 742.2082.

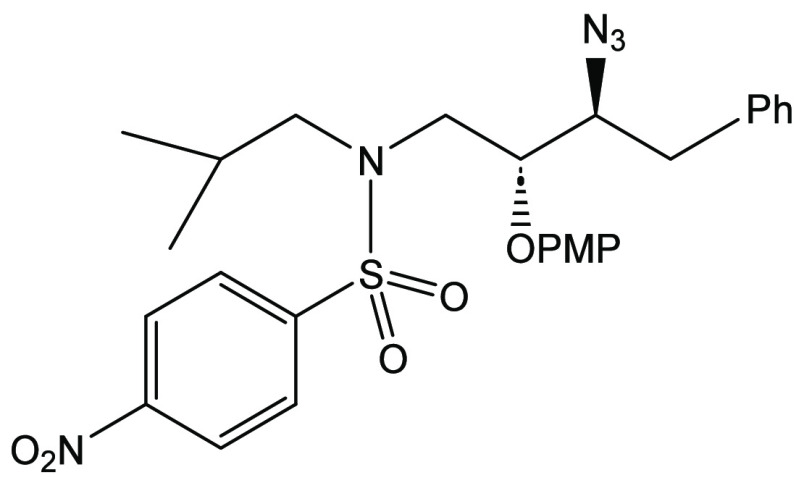



#### *N*-((2*R*,3*S*)-3-[Azido-2-(*p*-methoxyphenoxy)-4-phenylbutyl]-*N*-(2-isobutyl)-*p*-nitrobenzene sulfonamide (**21**)^28^

To a flame-dried, nitrogen-purged 100 mL round-bottom flask equipped with a stir bar were added bis-sulfonylated substrate **(13)** (2.00 g, 2.80 mmol) and DMSO (12 mL). Once the substrate had fully dissolved, sodium azide (0.551 g, 8.41 mmol) was added, and the system was allowed to stir overnight. The system was transferred to a separatory funnel, diethyl ether (150 mL) was added, and the material was extracted with 1 M HCl (2 × 40 mL). The organic layer was washed with brine (30 mL), dried over magnesium sulfate, and gravity-filtered. The solvent was then removed under reduced pressure to afford the target azide product as a yellow oil that was used in the next step without further purification (1.55 g, 2.79 mmol, near quantitative yield). [α]_D_ = +3.51 (*c* = 1.05, CHCl_3_). ^1^H NMR (500 MHz, CDCl_3_): δ 8.11 (d, *J* = 8.8 Hz, 2H), 7.84 (d, *J* = 8.8 Hz, 2H), 7.40–7.25 (m, 5H), 6.64 (d, *J* = 9.1 Hz, 2H), 6.37 (d, *J* = 9.1 Hz, 2H), 4.34 (dt, *J* = 9.3, 2.5 Hz, 1H), 3.94 (td, *J* = 7.6 Hz, 2.5 Hz, 1H), 3.75 (dd, *J* = 15.5, 2.3 Hz, 1H), 3.72 (s, 3H), 3.49 (dd, *J* = 15.5, 9.3 Hz, 1H), 3.23 (dd, *J* = 13.6, 8.1 Hz, 1H), 3.02 (dd, *J* = 13.6, 7.0 Hz, 1H), 2.86 (dd, *J* = 14.0, 7.9 Hz, 1H), 2.79 (dd, *J* = 14.0, 7.4 Hz, 1H), 2.08–2.00 (m, 1H), 0.91 (d, *J* = 6.6 Hz, 3H), 0.87 (d, *J* = 6.7 Hz, 3H) ppm. ^13^C{^1^H} NMR (100 MHz, CDCl_3_): δ 154.6, 149.8, 149.6, 146.0, 136.6, 129.3, 128.9, 128.1, 127.3, 124.2, 116.5, 114.7, 77.9, 63.7, 57.4, 55.6, 47.6, 37.2, 26.7, 20.0, 19.9 ppm. IR (CHCl_3_): 2119, 1532, 1350 cm^–1^. HRMS (ESI-TOF) *m*/*z*: [M + Na]^+^ Calcd for C_27_H_32_N_5_NaO_6_S 554.2068; Found, 554.2064.

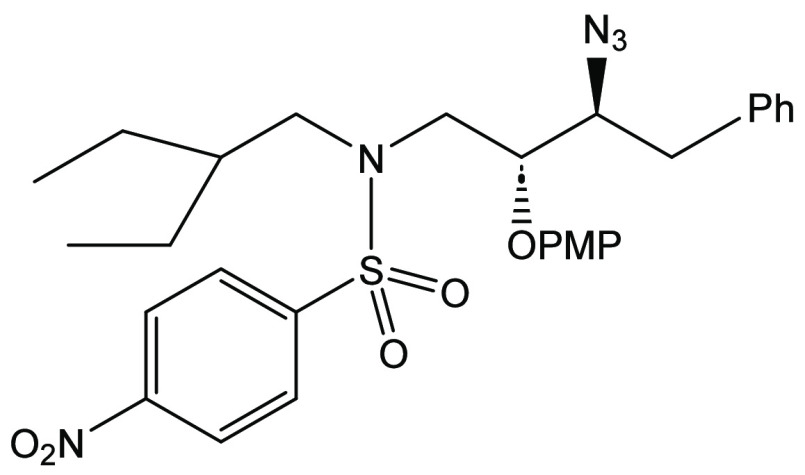



#### *N*-((2*R*,3*S*)-3-[Azido-2-(*p*-methoxyphenoxy)-4-phenylbutyl]-*N*-(2-ethylbutyl)-*p*-nitrobenzene sulfonamide (**22**)^29^

To a stirring solution of sulfonamide **20** (3.35 g, 4.52 mmol) in DMSO (30 mL) was added sodium azide (1.17 g, 18.0 mmol). The reaction mixture was stirred overnight at ambient temperature. After completion, the reaction mixture was diluted with diethyl ether (100 mL) and treated with an aqueous solution of 1 M HCl (2 × 30 mL). The organic layer was collected and treated with brine (30 mL), dried over MgSO_4_, and concentrated under reduced pressure. The residue was purified by flash column chromatography eluting with dichloromethane to afford **22** (2.52 g, 4.34 mmol, 96% yield) as a yellow viscous oil. ^1^H NMR (500 MHz, CDCl_3_): δ 8.13 (d, *J* = 8.9 Hz, 2H), 7.84 (d, *J* = 8.9 Hz, 2H), 7.40–7.24 (m, 5H), 6.64 (d, *J* = 9.1 Hz, 2H), 6.38 (d, *J* = 9.1 Hz, 2H), 4.36 (dt, *J* = 9.4, 2.6 Hz, 1H), 3.95 (td, *J* = 7.6, 2.6 Hz, 1H), 3.72 (s, 3H), 3.73 (dd, *J* = 15.6, 2.5 Hz, 1H), 3.49 (dd, *J* = 15.6, 9.4 Hz, 1H), 3.35 (dd, *J* = 13.6, 8.1 Hz, 1H), 3.05 (dd, *J* = 13.5, 6.7 Hz, 1H), 2.85 (dd, *J* = 14.1, 8.1 Hz, 1H), 2.78 (dd, *J* = 14.0, 7.3 Hz, 1H), 1.69–1.61 (m, 1H), 1.43–1.34 (m, 1H), 1.32–1.25 (m, 3H), 0.83 (q, *J* = 7.4 Hz, 6H) ppm. ^13^C{^1^H} NMR (100 MHz, CDCl_3_): δ 154.7, 149.8, 149.7, 145.8, 136.5, 129.2, 128.9, 128.1, 127.3, 124.2, 116.5, 114.7, 77.9, 63.6, 55.6, 53.5, 47.4, 38.5, 37.2, 23.0, 22.9, 10.6, 10.3 ppm. IR (CDCl_3_): 3105, 2119, 1607, 1530, 1506, 1350, 1224, 826 cm^–1^. HRMS (ESI-TOF) *m*/*z*: [M + Na]^+^ Calcd for C_29_H_35_N_5_NaO_6_S 604.2200; Found, 604.2205.

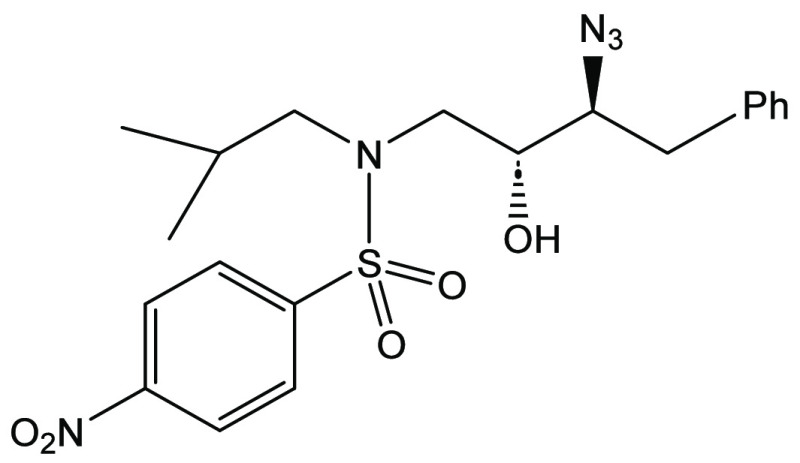



#### *N*-((2*R*,3*S*)-3-Azido-2-hydroxy-4-phenylbutyl)-*N*-(2-isobutyl)-4-nitrobenzenesulfonamide (**12**)^28^

To a flame-dried, nitrogen-purged 250 mL round-bottom flask equipped with a stir bar was added azide **21** (1.89 g, 3.41 mmol). Acetonitrile (56 mL) and deionized water (14 mL) were added in a 4:1 ratio to achieve an overall concentration of 0.049 M. Ceric ammonium nitrate (7.48 g, 13.7 mmol) was added, and the reaction was allowed to stir for 90 min. The reaction was then diluted with brine (20 mL), and the solvent was then removed by rotary evaporation. The concentrate was diluted with diethyl ether (150 mL), transferred to a separatory funnel, and extracted with brine (2 × 20 mL). The organic layer was dried over MgSO_4_, gravity-filtered, and concentrated under reduced pressure. The resulting crude residue was purified over silica using a mobile phase gradient (95:5, 80:20, hexanes:EtOAc) to afford the target azido alcohol as a dark, viscous oil (0.683 g, 1.53 mmol, 45% yield). [α]_D_ = −3.2 (*c* = 1.19, CHCl_3_). ^1^H NMR (500 MHz, CDCl_3_): δ 8.38 (d, *J* = 8.8 Hz, 2H), 7.99 (d, *J* = 8.8 Hz, 2H), 7.35–7.32 (m, 2H), 7.29–7.25 (m, 3H), 3.79–3.75 (m, 1H), 3.66–3.62 (m, 1H), 3.31 (dd, *J* = 15.2, 9.3 Hz, 1H), 3.20 (dd, *J* = 15.2, 2.4 Hz, 1H), 3.09–3.04 (m, 3H), 2.93 (dd, *J* = 13.5, 6.9 Hz, 1H), 2.83 (dd, *J* = 14.1, 8.9 Hz, 1H), 1.89–1.80 (m, 1H), 0.92 (d, *J* = 6.6 Hz, 3H), 0.87 (d, *J* = 6.6 Hz, 3H) ppm. ^13^C{^1^H} NMR (100 MHz, CDCl_3_): δ 150.1, 144.7, 137.1, 129.3, 128.8, 128.6, 127.0, 124.5, 71.6, 66.6, 58.0, 52.0, 36.8, 26.9, 20.0, 19.8 ppm. IR (CHCl_3_): 3515, 2111, 1531, 1350 cm^–1^. HRMS (ESI-TOF) *m*/*z*: [M + Na]^+^ Calcd for C_20_H_25_N_5_NaS 470.1469; Found, 470.1480.

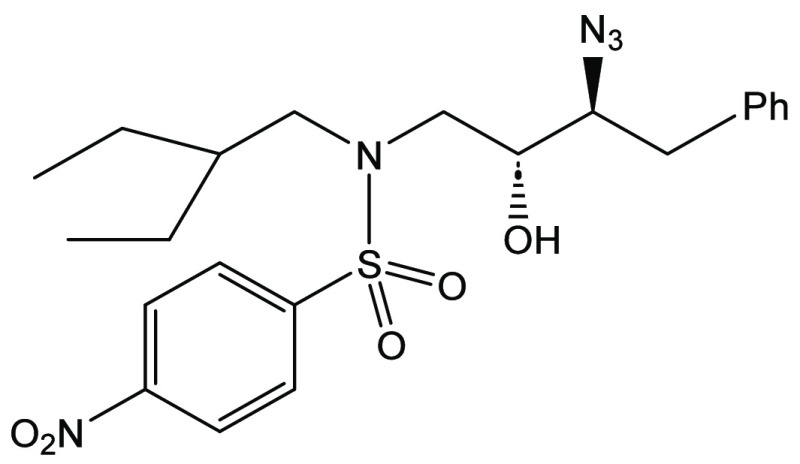



#### *N*-((2*R*,3*S*)-3-Azido-2-hydroxy-4-phenylbutyl)-*N*-(2-ethylbutyl)-4-nitrobenzenesulfonamide (**23**)^29^

To a stirred solution of **22** (2.01 g, 3.46 mmol) in acetonitrile (52.5 mL) was added ceric ammonium nitrate (7.58 g, 13.8 mmol), followed by deionized water (17.5 mL). The reaction mixture was stirred at room temperature for 1 h. The reaction mixture was then diluted with diethyl ether (100 mL), washed with brine (2 × 30 mL), dried over MgSO_4_, and concentrated under reduced pressure to afford a dark-red viscous oil. The residue was purified by flash column chromatography (2:8 diethyl ether: hexane) to afford **23** (0.661 g, 5.52 mmol, 40% yield) as a dark-red viscous oil. ^1^H NMR (500 MHz, CDCl_3_): δ 8.36 (d, *J* = 8.9 Hz, 2H), 7.99 (d, *J* = 8.9 Hz, 2H), 7.34–7.24 (m, 5H), 3.78–3.73 (broad multiplet, 1H), 3.66–3.32 (m, 1H), 3.30 (dd, *J* = 15.2, 9.2 Hz, 1H), 3.19 (dd, *J* = 15.3, 2.4 Hz, 1H), 3.16–3.11 (m, 2H), 3.05 (dd, *J* = 14.2, 4.7 Hz, 1H), 2.99 (dd, *J* = 13.4, 6.7 Hz, 1H), 2.83 (dd, *J* = 14.2, 8.8 Hz, 1H), 1.51–1.43 (m, 1H), 1.43–1.34 (m, 1H), 1.32–1.24 (m, 3H), 0.82 (q, *J* = 7.5 Hz, 6H) ppm. ^13^C{^1^H} NMR (100 MHz, CDCl_3_): δ 150.2, 144.3, 136.9, 129.3, 128.7, 128.6, 127.1, 124.5, 71.5, 66.5, 54.3, 52.2, 38.7, 36.8, 23.0, 22.7, 10.5, 10.3 ppm. IR (CHCl_3_): 3516, 3106, 2110, 1606, 1531, 1456, 1350, 1159, 1089, 856 cm^–1^. HRMS (ESI-TOF) *m*/*z*: [M + Na]^+^ Calcd for C_22_H_29_N_5_NaO_5_S 498.1782; Found, 498.1782.

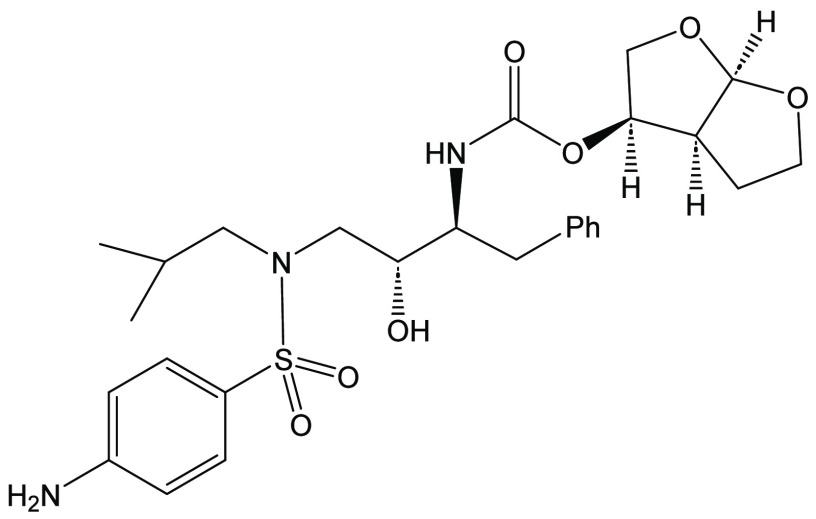



#### Darunavir (**1**)^28^

To a nitrogen-purged 250 mL round-bottom flask equipped with a stir bar were added azido alcohol (**12**) (0.680 g, 1.52 mmol), methanol (15 mL), and activated palladium on carbon (70 mg, 10% w/w). The flask was equipped with a hydrogen balloon, and the system was stirred for 17 h. The mixture was then filtered through Celite with ethyl acetate, concentrated under reduced pressure, and reconstituted in THF (15 mL). Commercially available carbonate **19** (0.412 g, 1.53 mmol) and triethylamine (0.32 mL, 2.3 mmol) were added, and the system was stirred overnight. The material was concentrated under reduced pressure and subsequently purified over silica using 50% EtOAc in dichloromethane as the eluent to yield darunavir **(1)** as an amorphous solid (0.43 g, 0.78 mmol, 52% yield). Mp: 74–75 °C. [α]_D_ = −2.40 (*c* = 1.04, CHCl_3_). ^1^H NMR (500 MHz, CDCl_3_): δ 7.55 (d, *J* = 8.0 Hz, 2H), 7.29–7.19 (m, 5H), 6.68 (d, *J* = 8.0 Hz, 2H), 5.64 (d, *J* = 5.1 Hz, 1H), 5.03–4.95 (m, 2H), 4.34–4.08 (broad singlet, 2H), 3.96–3.92 (m, 1H), 3.90–3.82 (m, 3H), 3.73–3.65 (m, 3H), 3.19–2.75 (m, 7H), 1.87–1.77 (m, 1H), 1.67–1.58 (m, 1H), 1.54–1.44 (m, 1H), 0.93 (d, *J* = 6.6 Hz, 3H), 0.88 (d, *J* = 6.6 Hz, 3H) ppm. ^13^C{^1^H} NMR (125 MHz, CDCl_3_): δ 155.5, 151.0, 137.8, 129.5, 129.4, 128.5, 126.5, 125.9, 114.1, 109.3, 73.4, 72.9, 70.9, 69.6, 58.8, 55.2, 53.7, 45.4, 35.7, 27.3, 25.8, 20.2, 20.0 ppm. IR (CHCl_3_): 3492, 3415, 1717 cm^–1^. HRMS (ESI-TOF) *m*/*z*: [M + Na]^+^ Calcd for C_27_H_38_N_3_NaO_7_S, 548.2425; Found, 548.2418.

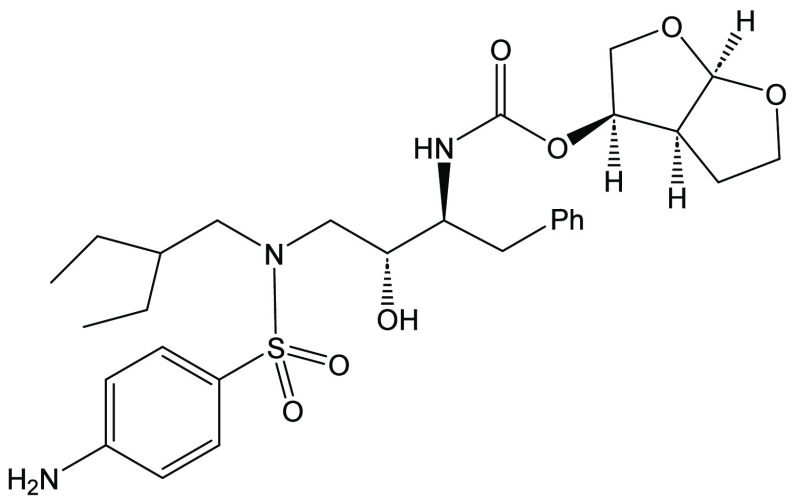



#### (3*R*,3*aS*,6*aR*)-Hexahydrofuro[2,3-*b*]furan-3-yl((2*S*,3*R*)-4-(4-amino-*N*-(2-ethylbutyl)phenyl sulfonamido)-3-hydroxy-1-phenyl-2-butyl)carbamate (**27**)^29^

A round-bottom flask containing **23** (0.356 g, 0.749 mmol), 5% palladium on carbon (0.004 g), 2,5-dioxopyrrolidin-1-yl((3*R*,3*aS*,6*aR*)-hexahydrofuro[2,3-*b*]furan-3-yl)carbonate (0.162 g, 0.598 mmol), and THF (25 mL) was flushed with N_2_ gas for 10 min. A balloon filled with hydrogen gas was then connected to the reaction flask, and the mixture was allowed to stir overnight at ambient temperature. After completion, the reaction mixture was diluted with chloroform, filtered through a 1:1 mixture of Celite and MgSO_4_, and concentrated under reduced pressure. The crude residue was purified by flash column chromatography (diethyl ether) to afford the protease inhibitor **27** (0.217 g, 0.350 mmol, 50% yield) as a pale-yellow viscous oil. [α]_D_ = +2.6 (*c* = 1.00, CDCl_3_). ^1^H NMR (500 MHz, CDCl_3_): δ 7.54 (d, *J* = 8.7 Hz, 2H), 7.29–7.18 (m, 5H) 6.68 (d, *J* = 8.7 Hz, 2H), 5.63 (d, *J* = 5.1 Hz, 1H), 5.00 (dd, *J* = 13.8, 6.5 Hz, 1H), 4.91 (d, *J* = 8.8 Hz, 2H), 4.17 (broad singlet, 2H), 3.94 (dd, *J* = 9.4, 6.4 Hz, 1H), 3.90–3.79 (m, 3H), 3.76 (broad singlet, 1H), 3.71–3.66 (m, 2H), 3.15–3.06 (m, 2H), 3.02 (dd, *J* = 13.4, 8.0 Hz, 1H), 2.96 (dd, *J* = 15.3, 2.3 Hz, 1H), 2.92–2.87 (m, 1H), 2.84–2.77 (m, 2H), 2.67–2.58 (m, 2H), 1.51–1.39 (m, 3H), 0.82 (td, *J* = 7.4, 2.3 Hz, 6H) ppm.^13^C{^1^H} NMR (100 MHz, CDCl_3_): δ 155.5, 151.3, 129.5, 129.4, 128.4, 126.5, 125.2, 114.0, 109.3, 73.3, 73.1, 70.8, 69.6, 55.3, 54.7, 53.6, 45.5, 39.0, 35.8, 25.8, 23.0, 22.8, 10.5, 10.3 ppm. IR (CHCl_3_): 3469, 3368, 3251, 1706, 832 cm^–1^. HRMS (ESI-TOF) *m*/*z*: [M + Na]^+^ Calcd for C_29_H_42_N_3_NaO_7_S 576.2738; Found, 576.2739.

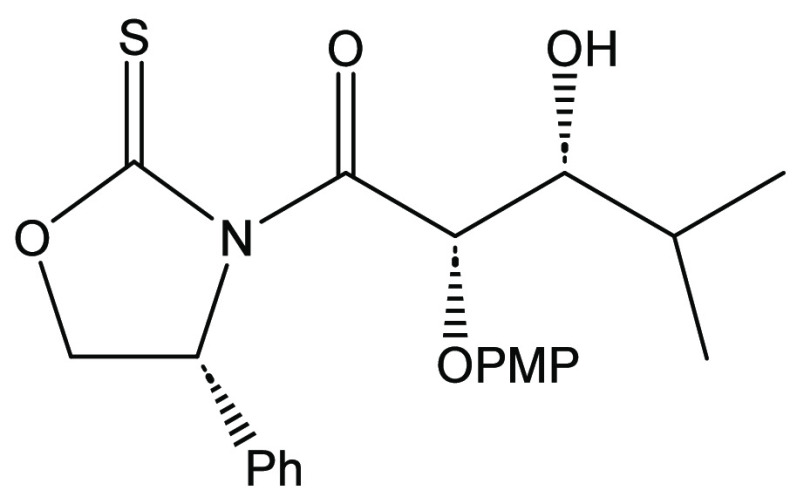



#### (4*R*)-3-[(2*S*’,3*R*’)-3-Hydroxy-2-(*p*-methoxyphenoxy)-4-methyl-pentanoyl]-1,3-oxazolidine-2-thione (**28**)^29^

A flame-dried 3 L round-bottom flask fitted with a Claisen adapter and an addition funnel was fitted onto the Claisen adapter was charged with (*R*)-**11** (10.0 g, 29.1 mmol) and dichloromethane (1 L). The reaction mixture was cooled to −78 °C and titanium tetrachloride (1 M in dichloromethane, 32.0 mL, 32.0 mmol) was added via the addition funnel. The reaction mixture was stirred for 30 min, and then, triethylamine (8.9 mL, 64 mmol) was added dropwise by syringe. After an additional 30 min of stirring, the second batch of titanium tetrachloride (32 mL) was added as before. After another 30 min of stirring, isobutyraldehyde (5.8 mL, 64 mmol) was added dropwise to the reaction mixture by syringe. Finally, the reaction mixture was warmed to −40 °C for 3 h and then quenched with brine (80 mL). The reaction mixture was diluted with dichloromethane and then washed with an aqueous solution of 1 M HCl (2 × 80 mL). The extracted organic layer was washed with brine (80 mL), dried over MgSO_4_, and concentrated under reduced pressure. The resulting crude viscous oil was crystallized from ethyl acetate and hexanes to afford compound **28** as a white crystalline solid (8.89 g, 21.5 mmol, 74% yield). Mp: 149.5–151.5 °C. [α]_D_ = −102.9 (*c* = 0.46, CDCl_3_). ^1^H NMR (500 MHz, CDCl_3_): δ 7.39–7.29 (m, 5H), 7.02 (d, *J* = 1.8 Hz, 1H), 6.85–6.79 (m, 4H), 5.73 (dd, *J* = 9.3, 6.5 Hz, 1H), 4.88 (t, *J* = 9.3 Hz, 1H), 4.50 (dd, *J* = 9.3, 6.5 Hz, 1H), 3.93 (ddd, *J* = 10.2, 8.4, 1.8 Hz, 1H), 3.74 (s, 3H), 2.05–1.95 (m, 1H), 1.60 (d, *J* = 10.2 Hz, 1H), 1.06 (d, *J* = 6.7 Hz, 3H), 1.01 (d, *J* = 6.7 Hz, 3H) ppm. ^13^C{^1^H} NMR (100 MHz, CDCl_3_): δ 185.5, 171.1, 154.6, 151.2, 136.7, 129.2, 129.1, 126.6, 116.0, 114.8, 77.5, 76.7, 74.7, 62.8, 55.7, 32.4, 19.2, 19.1 ppm. IR (CDCl_3_): 3454, 1727, 1228, 825 cm^–1^. HRMS (ESI-TOF) *m*/*z*: [M + Na]^+^ Calcd for C_22_H_26_NNaO_5_S 416.1526; Found, 416.1523.

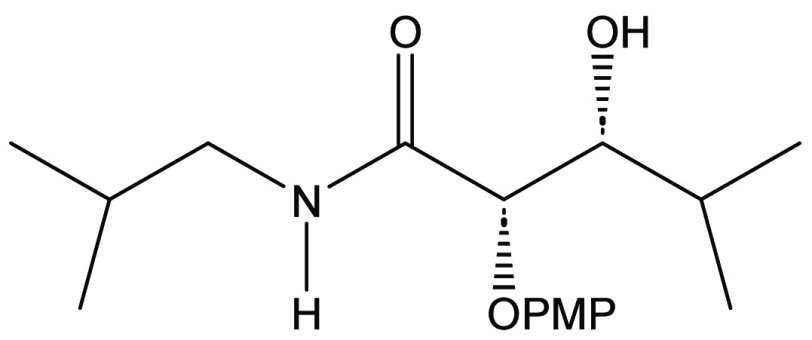



#### (2*S*,3*R*)-3-Hydroxy-*N*-isobutyl-2-(*p*-methoxyphenoxy)-4-methylpentanamide (**30**)^29^

To a stirring solution of aldol adduct **28** (5.36 g, 12.9 mmol) in dichloromethane (129 mL) was added imidazole (2.63 g, 38.6 mmol). After 1 h, isobutylamine (1.30 mL, 13.1 mmol) was added to the reaction mixture, and the reaction was stirred overnight. The reaction mixture was diluted with dichloromethane (30 mL) and washed twice with an aqueous solution of NaOH (2 × 40 mL). The organic layer was then washed with brine (40 mL), dried over MgSO_4_, and concentrated under reduced pressure to afford compound **33a** (3.80 g, 12.2 mmol, 95% yield) as pure white crystals. The crystals obtained were pure and did not require further purification. Mp: 84–86 °C. ^1^H NMR (500 MHz, CDCl_3_): δ 6.88 (d, *J* = 9.3 Hz, 2H), 6.83 (d, *J* = 9.3 Hz, 2H), 6.49 (broad triplet, 1H), 4.56 (d, *J* = 2.6 Hz, 1H), 4.30 (broad singlet, 1H), 3.77 (s, 3H), 3.73 (dd, *J* = 7.8, 2.6 Hz, 1H), 3.12 (t, *J* = 6.6 Hz, 2H), 2.01–1.92 (m, 1H), 1.78–1.70 (m, 1H), 1.06 (d, *J* = 6.7 Hz, 3H), 0.89 (d, *J* = 6.7 Hz, 3H), 0.84 (d, *J* = 3.7 Hz, 3H), 0.83 (d, *J* = 3.7 Hz, 6H) ppm. ^13^C{^1^H} NMR (100 MHz, CDCl_3_): δ 170.7, 154.9, 151.6, 116.2, 114.9, 80.6, 55.7, 46.4, 31.0, 28.5, 19.94, 19.90, 19.3, 18.7 ppm. IR (CDCl_3_): 3470, 3338, 1634, 1508, 1465, 1224, 816.5 cm^–1^. HRMS (ESI-TOF) *m*/*z*: [M + H]^+^ Calcd for C_17_H_28_NO_4_, 310.2013; Found, 310.2018.

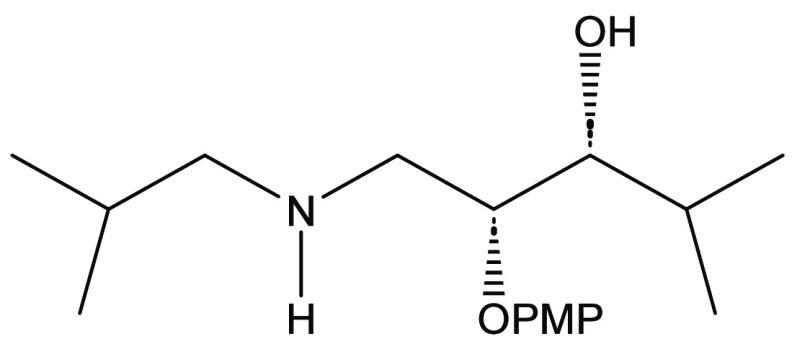



#### (2*R*,3*R*)-1-(Isobutylamino)-2-(*p*-methoxyphenoxy)-4-methyl-3-pentanol (**31**)^29^

To a round-bottom flask connected to nitrogen lines and mounted on a jacked stir plate were added compound **30** (3.27 g, 10.6 mmol) and THF (265 mL). A borane dimethylsulfide complex (3.00 mL) was then added to the reaction mixture while stirring at room temperature. The reaction was then heated to reflux overnight using a heating mantle controlled by a variable transformer. The reaction was then cooled with an ice bath and quenched by the addition of methanol (15 mL). After stirring for 30 min, the solvent was removed via rotary evaporation, and the reaction mixture was diluted with diethyl ether (120 mL) and then washed with an aqueous solution of 1 M NaOH (2 × 30 mL) and brine (30 mL). The organic layer was dried over MgSO_4_ and concentrated under reduced pressure. The residue was purified by flash column chromatography (7:3, diethyl ether:hexanes) to afford compound **31** (2.56 g, 8.6 mmol, 82% yield) as a colorless viscous oil. ^1^H NMR (500 MHz, CDCl_3_): δ 6.92 (d, *J* = 9.2 Hz, 2H), 6.83 (d, *J* = 9.2 Hz, 2H), 4.34–4.33 (m, 1H), 3.77 (s, 3H), 3.51 (dd, *J* = 8.4, 2.0 Hz, 1H), 3.33 (dd, *J* = 12.6, 4.2 Hz, 1H), 2.79 (dd, *J* = 12.6, 2.4 Hz, 1H), 2.44 (dd, *J* = 11.5, 6.4 Hz, 1H), 2.35 (dd, *J* = 11.5, 7.1 Hz, 1H), 2.03–1.96 (m, 1H), 1.75–1.67 (m, 1H), 1.05 (d, *J* = 6.7 Hz, 3H), 0.89 (d, *J* = 4.3 Hz, 3H), 0.88 (d, *J* = 4.3 Hz, 3H), 0.87 (d, *J* = 6.7 Hz, 3H) ppm. ^13^C{^1^H} NMR (100 MHz, CDCl_3_): δ 154.2, 151.8, 117.1, 114.8, 81.0, 75.7, 58.1, 55.7, 51.0, 30.7, 28.2, 20.5, 19.1, 19.0 ppm. IR (CDCl_3_): 3325, 3224, 1226, 1039, 827 cm^–1^. HRMS (ESI-TOF) *m*/*z*: [M + H]^+^ Calcd for C_17_H_30_NO_3_ 296.2220; Found, 296.2220.

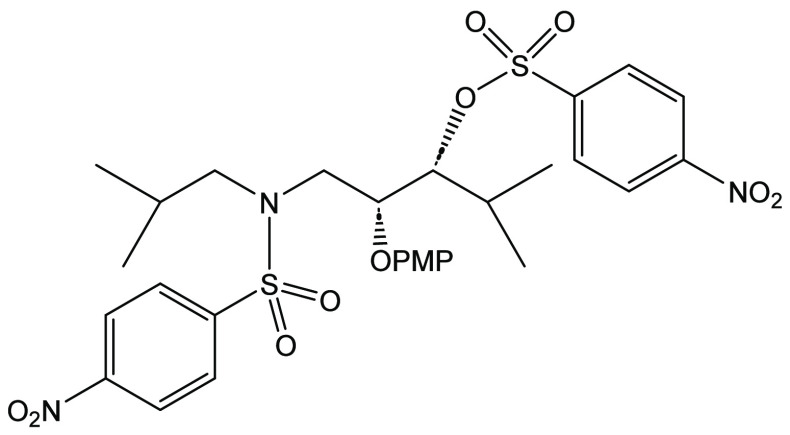



#### (2*R*,3*R*)-1-(*N*-Isobutyl-P-nitrophenylsulfonamido)-2-(*p*-methoxyphenoxy)-*p*-methyl-3-pentyl*-p*-nitrobenzenesulfonate (**32**)^29^

To a vigorously stirring solution of **31** (1.92 g, 6.50 mmol) in dichloromethane (23 mL) in a round-bottom flask was added *p*-nitrobenzenesulfonyl chloride (3.60 g, 16.2 mmol), followed by DMAP (0.874 g, 7.15 mmol). Triethylamine (3.60 mL, 25.8 mmol) was then added dropwise to the reaction mixture via an addition funnel. The reaction mixture was then stirred at ambient temperature overnight. The reaction mixture was diluted with dichloromethane (50 mL) and washed with an aqueous solution of 1 M HCl (25 mL) and brine (25 mL). The organic layer was then isolated and dried over MgSO_4_ and concentrated under reduced pressure to afford a crude yellow viscous oil that was crystallized from diethyl ether and hexanes to afford **32** (2.45 g, 57% yield) as yellow crystals. Mp: 132.6–133.9 °C. ^1^H NMR (500 MHz, CDCl_3_): δ 8.19 (dd, *J* = 14.1, 8.9 Hz, 4H), 7.98 (d, *J* = 8.9 Hz, 2H), 7.87 (d, *J* = 8.9 Hz, 2H), 6.62 (d, *J* = 9.1 Hz, 2H), 6.51 (d, *J* = 9.1 Hz, 2H), 4.66–4.63 (m, 1H), 4.55 (t, *J* = 5.9 Hz, 1H), 3.73 (s, 3H), 3.67 (dd, *J* = 15.3, 1.8 Hz, 1H), 3.4 (dd, *J* = 15.3, 9.3 Hz, 1H), 3.11 (dd, *J* = 13.8, 8.3 Hz, 1H), 2.99 (dd, *J* = 13.8, 6.9 Hz, 1H), 2.06–1.93 (m, 2H), 1.02 (d, *J* = 6.7 Hz, 3H), 0.89 (d, *J* = 6.7 Hz, 3H) 0.83 (d, *J* = 6.7 Hz, 6H) ppm. ^13^C{^1^H} NMR (100 MHz, CDCl_3_): δ 154.7, 150.6, 150.5, 149.8, 145.6, 142.0, 129.0, 128.1, 124.3, 124.1, 116.2, 114.5, 86.8, 57.0, 55.6, 48.5, 29.0, 26.5, 19.8, 19.7, 18.2 ppm. IR (CDCl_3_): 3108, 1607, 1350, 855 cm^–1^. HRMS (ESI-TOF) *m*/*z*: [M + Na]^+^ Calcd for C_29_H_35_N_3_NaO_11_S_2_ 688.1605; Found, 688.1605.

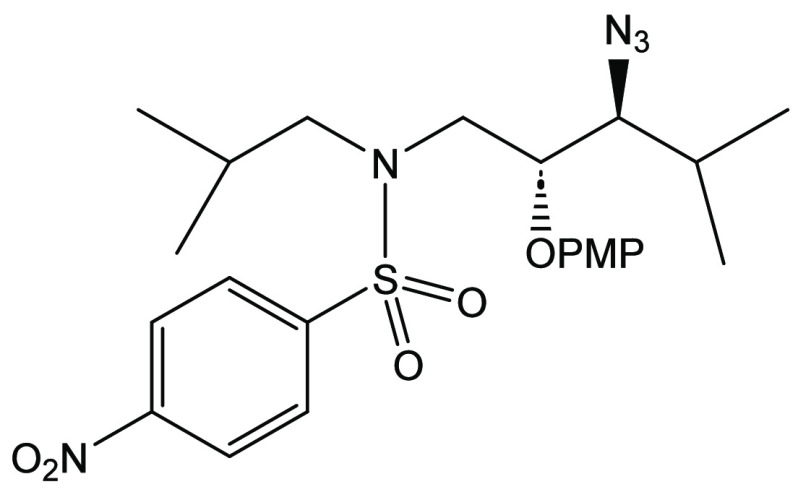



#### *N*-((2*R*,3*S*)-3-Azido-2-(*p*-methoxyphenoxy)-4-methylpentyl)-*N*-isobutyl-*p*-nitrobenzene sulfonamide (**33**)^29^

To a solution of **32** (2.00 g, 3.00 mmol) in DMSO (20 mL) was added sodium azide (0.781 g). The reaction mixture was stirred overnight at ambient temperature, diluted with diethyl ether (100 mL), and washed twice with an aqueous solution of 1 M HCl (2 × 30 mL). The organic layer was then washed with brine (30 mL), dried over MgSO_4_, and concentrated under reduced pressure. The residue was purified by flash column chromatography eluting using dichloromethane to afford azide **33** (1.40 g, 2.75 mmol, 92% yield) as a yellow viscous oil. ^1^H NMR (500 MHz, CDCl_3_): δ 8.23 (d, *J* = 8.9 Hz, 2H), 7.94 (d, *J* = 8.9 Hz, 2H), 6.77 (d, *J* = 9.1 Hz, 2H), 6.69 (d, *J* = 9.1 Hz, 2H), 4.68 (dt, *J* = 9.7, 2.4 Hz, 1H), 3.75 (s, 3H), 3.57 (dd, *J* = 15.6, 2.1 Hz, 1H), 3.42 (dd, *J* = 15.6, 9.6 Hz, 1H), 3.33 (dd, *J* = 9.6, 2.7 Hz, 1H), 3.27 (dd, *J* = 13.5, 8.7 Hz, 1H), 3.30 (dd, *J* = 13.5, 6.4 Hz, 1H), 2.11–2.03 (m, 1H), 1.67–1.60 (m, 1H), 1.05 (d, *J* = 6.7 Hz, 3H), 1.01 (d, *J* = 6.7 Hz, 3H), 0.89 (d, *J* = 6.7 Hz, 3H), 0.84 (d, *J* = 6.7 Hz, 3H) ppm. ^13^C{^1^H} NMR (100 MHz, CDCl_3_): δ 154.7, 150.0, 149.8, 145.8, 128.2, 124.2, 116.9, 114.9, 78.2, 69.5, 57.8, 55.6, 48.0, 30.2, 26.6, 20.6, 20.0, 19.9, 19.2, 18.6 ppm. IR (CDCl_3_): 3105, 1606, 1350, 856 cm^–1^. HRMS (ESI-TOF) *m*/*z*: [M + Na]^+^ Calcd for C_23_H_31_N_5_NaO_6_S 528.1887; Found, 528.1895.

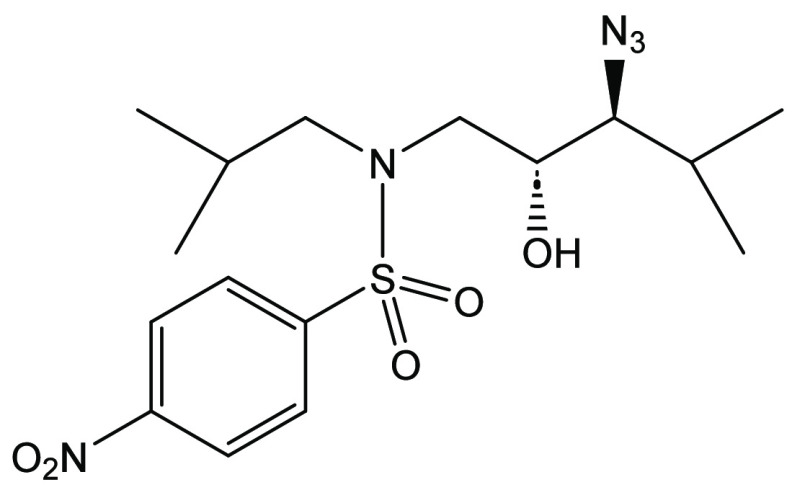



#### *N*-((2*R*,3*S*)-3-Azido-2-hydroxy-4-methylpentyl)-*N*-isobutyl-*p*-nitrobenzenesulfonamide (**34**)^29^

To a stirred solution of compound **33** (1.11 g, 2.20 mmol) in acetonitrile (33 mL) were added cerium (IV) ammonium nitrate (4.83 g, 8.81 mmol) and deionized water (11 mL). The reaction mixture was stirred at ambient temperature for 1 h. After completion, the reaction mixture was diluted with diethyl ether, washed twice with brine, dried over MgSO_4_, and concentrated under reduced pressure to afford a dark-red viscous oil. The residue was purified by flash column chromatography (2:8, diethyl ether:hexane) to afford β-azido alcohol **34** (0.399 g, 0.99 mmol, 45% yield) as a dark-red viscous oil. ^1^H NMR (500 MHz, CDCl_3_): δ 8.39 (d, *J* = 8.9 Hz, 2H), 8.02 (d, *J* = 8.9 Hz, 2H), 3.88 (broad triplet, *J* = 7.9 Hz, 1H), 3.31 (dd, *J* = 15.2, 9.6 Hz, 1H), 3.19 (t, *J* = 6.2, Hz, 1H), 3.15 (dd, *J* = 15.2, 2.2 Hz, 1H), 3.09 (dd, *J* = 13.6, 8.2 Hz, 1H), 2.98 (dd, *J* = 13.6, 7.1 Hz, 2H), 2.0–1.87 (m, 2H), 1.04 (d, *J* = 6.7 Hz, 3H), 1.00 (d, *J* = 6.7 Hz, 3H), 0.95 (d, *J* = 6.7 Hz, 3H), 0.90 (d, *J* = 6.7 Hz, 3H) ppm. ^13^C{^1^H} NMR (100 MHz, CDCl_3_): δ 150.2, 144.6, 128.6, 124.4, 72.2, 70.1, 58.3, 52.4, 29.9, 27.1, 20.1, 20.0, 19.8, 18.0 ppm. IR (CDCl_3_): 3519, 3107, 2104, 1607, 1531, 1350, 856 cm^–1^. HRMS (ESI-TOF) *m*/*z*: [M + Na]^+^ Calcd for C_16_H_25_N_5_NaO_5_S 422.1469; Found, 422.1469.

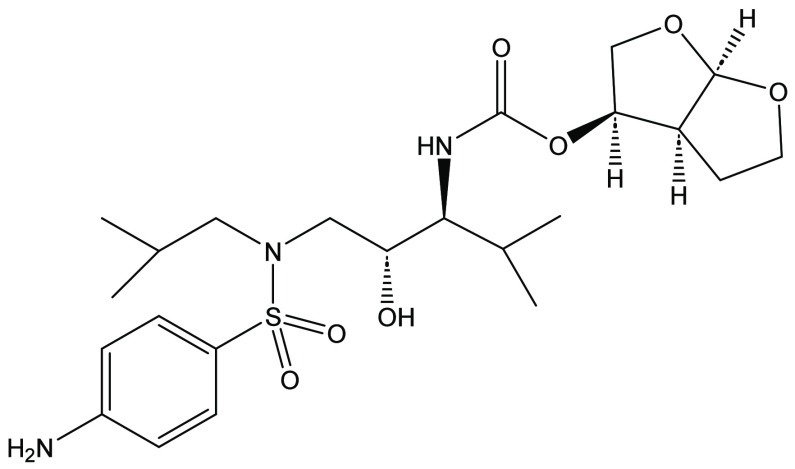



#### (3*R*,3*aS*,6*aR*)-Hexahydrofuro[2,3-*b*]furan-3-yl((2*R*,3*S*)-1-(4-amino-*N*-isobutylphenyl sulfonamido)-2-hydroxy-4-methylpentan-3-yl)carbamate (**35**)^29^

A round-bottom flask containing compound **34** (0.100 g, 0.250 mmol), 5% palladium on carbon (0.001 g), 2,5-dioxopyrrolidin-1-yl((3*R*,3*aS*,6*aR*)-hexahydrofuro[2,3-*b*]furan-3-yl)carbonate (0.0611 g), and THF (8.30 mL) was flushed with N_2_ gas for 10 min. A balloon filled with H_2_ gas was then connected to the reaction flask, and the mixture was allowed to stir overnight at room temperature. After completion, the reaction mixture was diluted with chloroform, filtered through a 1:1 mixture of Celite and MgSO_4_, and concentrated under reduced pressure. The crude residue was purified by flash column chromatography (diethyl ether) to afford the protease inhibitor **35** (0.0908 g, 73% yield) as a rusty viscous oil. [α]_D_ = +10.1 (*c* = 0.490, CHCl_3_). ^1^H NMR (500 MHz, CDCl_3_): δ 7.55 (d, *J* = 8.6 Hz, 2H), 6.68 (d, *J* = 8.6 Hz, 2H), 5.71 (d, *J* = 5.1 Hz, 1H), 5.12 (dd, *J* = 14.6, 6.7 Hz, 1H), 4.84 (d, *J* = 10.0 Hz, 2H), 4.20 (br.s, 2H), 4.02 (dd, *J* = 9.5, 6.5 Hz, 1H) 3.98 (td, *J* = 8.4, 2.6 Hz, 1H) 3.92–3.87 (m, 1H), 3.78–3.71 (m, 2H), 3.48–3.43 (m, 1H), 3.36 (d, *J* = 2.5 Hz, 1H), 3.07 (dd, *J* = 15.2, 8.5 Hz, 2H), 3.01 (dd, *J* = 15.4, 2.7 Hz, 1H), 2.91 (dd, *J* = 13.1, 7.6 Hz, 1H), 2.82 (dd, *J* = 13.3, 7.1 Hz, 1H), 2.29–2.22 (m, 1H), 2.07–2.01 (m, 1H), 1.95–1.78 (m, 2H), 0.93 (dd, *J* = 6.7, 4.3 Hz, 6H), 0.89 (d, *J* = 6.7 Hz, 6H) ppm. ^13^C{^1^H} NMR (100 MHz, CDCl_3_): δ 156.1, 151.1, 129.5, 125.5, 114.0, 109.3, 73.4, 71.6, 70.8, 69.5, 58.8, 58.5, 54.3, 53.9, 45.3, 27.3, 27.1, 25.8, 20.2, 20.1, 20.0, 15.9 ppm. IR (CDCl_3_): 3466, 3368, 3252, 1708, 1632,1149, 1091, 830 cm^–1^. HRMS (ESI-TOF) *m*/*z*: [M + H]^+^ Calcd for C_23_H_38_N_3_O_7_S 500.2425; Found, 500.2426.

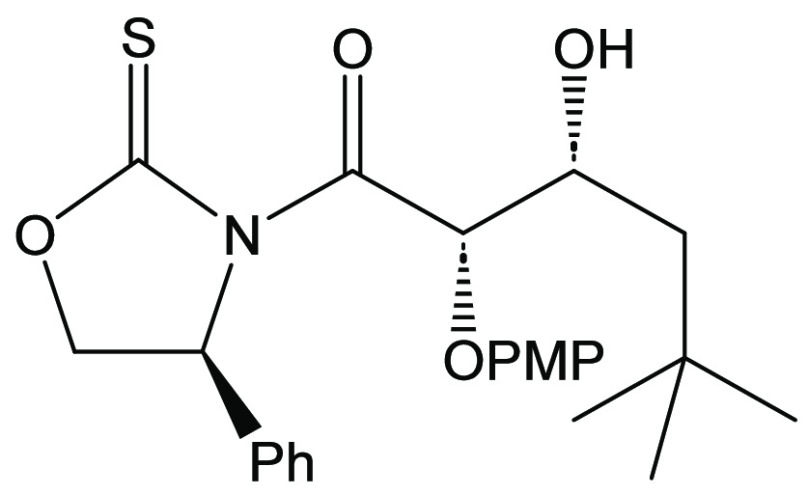



#### (4*S*)-[(2*S*’,3*R*’)-3-Hydroxy-2-(*p*-methoxyphenoxy)-5,5-dimethylhexanoyl]-4-phenyl-1,3-oxazolidine-2-thione (**39**)^29^

A flame-dried, nitrogen-purged 5 L round-bottom flask fitted with a Claisen adapter for an additional funnel was charged with acylated oxazolidine-2-thione (*S*)-**11** (10.0 g, 29.1 mmol) and dichloromethane (1 L). The reaction mixture was cooled to −78 °C, and titanium tetrachloride (1 M in dichloromethane, 32 mL, 32 mmol) was added via the addition funnel. The reaction mixture was stirred for 30 min, then treated with triethylamine (8.9 mL, 64 mmol), and added dropwise by syringe. After another 30 min of stirring, 3,3-dimethylbutyraldehyde (6.9 mL, 64 mmol) was added dropwise to the reaction mixture by syringe. Finally, the reaction mixture was warmed to −40 °C for 3 h and then quenched with brine (80 mL). The reaction mixture was diluted with dichloromethane and treated with an aqueous solution of 1 M HCl (2 × 100 mL). The recovered organic layer was treated with brine (100 mL), dried over MgSO_4_, and concentrated under reduced pressure. The crude residue was purified by flash column chromatography on silica gel (15:85, diethyl ether:hexanes) to afford aldol adduct **39** (10.62 g, 25.0 mmol, 82% yield) as a pale-yellow viscous oil. [α]_D_ = +70.4 (*c* = 1.00, CHCl_3_). ^1^H NMR (500 MHz, CDCl_3_): δ 7.34–7.29 (m, 3H), 7.23–7.21 (m, 2H), 6.67 (d, *J* = 2.0 Hz, 1H), 6.66–6.61 (m, 4H), 5.68 (dd, *J* = 8.3, 2.5 Hz, 1H), 4.84 (t, *J* = 8.7 Hz, 1H), 4.53–4.47 (m, 2H), 3.70 (s, 3H), 1.71 (dd, *J* = 14.7, 9.5 Hz, 1H), 1.61 (dd, *J* = 14.6, 2.0 Hz, 1H), 1.02 (s, 9H) ppm. ^13^C{^1^H} NMR (100 MHz, CDCl_3_): 185.4, 169.6, 154.5, 151.3, 138.3, 129.2, 128.9, 125.8, 116.2, 114.6, 80.2, 74.7, 70.2, 62.8, 55.7, 47.8, 30.5, 30.2 ppm. IR (CDCl_3_): 3422, 1716, 1507, 1228, 1196, 824 cm^–1^. HRMS (ESI-TOF) *m*/*z*: [M + H]^+^ Calcd for C_24_H_30_NO_5_S, 444.1839; Found, 444.1838.

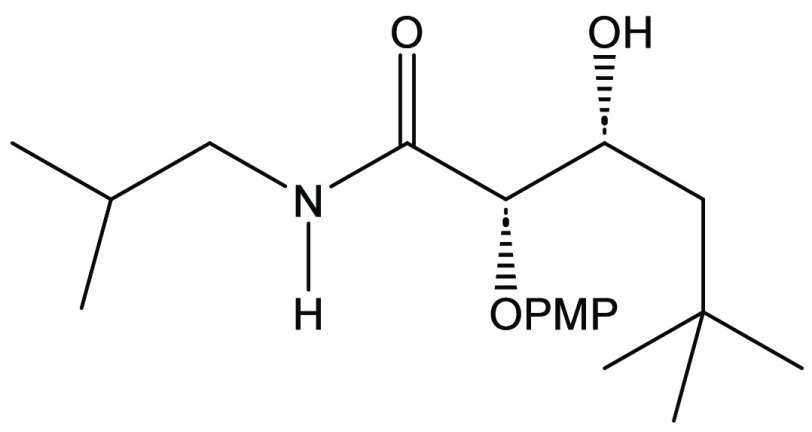



#### (2*S*,3*R*)-3-Hydroxy-*N*-isobutyl-2-(*p*-methoxyphenoxy)-5,5-dimethylhexanamide (**41**)^29^

To a stirred solution of oxazolidine-2-thione **39** (10.1 g, 21.8 mmol) in dichloromethane (218 mL) was added imidazole (4.45 g, 65.4 mmol). After 1 h, isobutylamine (2.40 mL, 24.0 mmol) was added, and the reaction mixture was allowed to stir overnight at room temperature. After completion, the reaction mixture was diluted with dichloromethane and then washed with an aqueous solution of 2 M NaOH (2 × 50 mL). The organic layer was treated with brine (50 mL), dried over MgSO_4_, and concentrated under reduced pressure. The crude residue was purified by flash column chromatography on silica gel (dichloromethane) to afford **41** (7.08 g, 20.9 mmol, 96% yield) as a colorless viscous oil. ^1^H NMR (500 MHz, CDCl_3_): δ 6.89 (d, *J* = 9.3 Hz, 2H), 6.83 (d, *J* = 9.3 Hz, 2H), 6.62 (broad triplet, 1H), 4.46 (d, *J* = 3.8 Hz, 1H), 4.11 (dq, *J* = 9.1, 2.2 1H), 3.77 (s, 3H), 3.19–3.08 (m, 1H), 1.81–1.73 (m, 1H), 1.51 (dd, *J* = 14.4, 2.1 Hz, 1H), 1.45 (dd, *J* = 14.4, 9.2 Hz, 1H), 0.95 (s, 9H), 0.89 (d, *J* = 2.5 Hz, 3H), 0.87 (d, *J* = 2.5 Hz, 3H) ppm. ^13^C{^1^H} NMR (125 MHz, CDCl_3_): δ 170.7, 155.0, 151.2, 116.8, 114.9, 82.2, 69.4, 55.7, 46.3, 46.0, 30.1, 30.0, 28.4, 20.0, 19.9 ppm. IR (CDCl_3_): 3430, 3354, 3072, 1224, 1058, 827 cm^–1^. HRMS (ESI-TOF) *m*/*z*: [M + H]^+^ Calcd for C_19_H_32_NO_4_ 338.2326; Found, 338.2317.

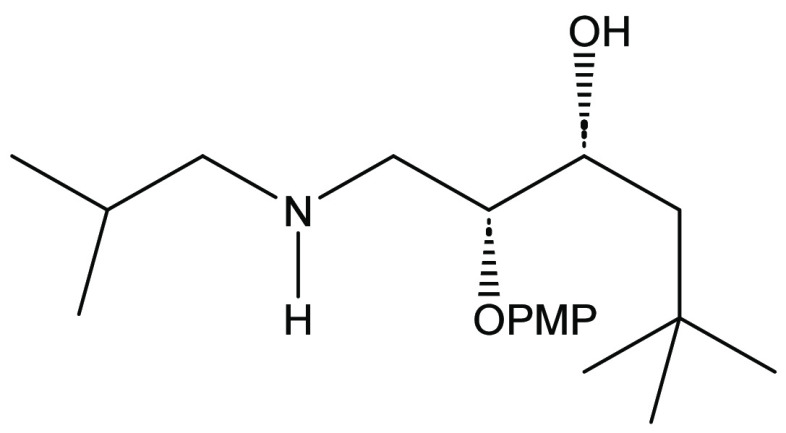



#### (2*R*,3*R*)-1-(Isobutylamino)-2-(*p*-methoxyphenoxy)-5,5-dimethyl-3-hexanol (**42**)^29^

To a flame-dried, nitrogen-purged 2 L round-bottom flask equipped with a magnetic stir bar were added amide **41** (6.64 g, 19.7 mmol) and THF (493 mL). A borane dimethylsulfide complex (5.60 mL) was added to the reaction mixture, and the reaction was then heated to reflux overnight using a heating mantle controlled by a variable transformer. The reaction was then cooled using an ice bath and quenched by the dropwise addition of methanol (30 mL). The reaction mixture was stirred for an additional 30 min, and the reaction solvent was removed via rotary evaporation. The concentrated reaction mixture was then diluted with diethyl ether (150 mL) and treated with an aqueous solution of 1 M NaOH (2 × 50 mL). The organic layer was washed one more time with brine (50 mL), dried over MgSO_4_, and concentrated under reduced pressure. The residue was purified by flash column chromatography on silica gel (7:3, diethyl ether:hexanes) to afford **42** (5.95 g, 18.3 mmol, 93% yield) as a colorless viscous oil. ^1^H NMR (500 MHz, CDCl_3_): δ 6.91 (d, *J* = 9.2 Hz, 2H), 6.82 (d, *J* = 9.2 Hz, 2H), 4.11–4.06 (m, 2H), 3.76 (s, 3H), 3.19 (dd, *J* = 12.5, 5.2 Hz, 1H), 2.85 (dd, *J* = 12.5, 3.2 Hz, 1H), 2.43–2.36 (m, 2H), 1.75–1.64 (m, 1H), 1.59 (dd, *J* = 14.4, 8.5 Hz, 1H), 1.45 (dd, *J* = 14.5, 2.2 Hz, 1H), 0.97 (s, 9H), 0.89 (d, *J* = 2.8 Hz, 1H), 0.88 (d, *J* = 2.8 Hz, 1H) ppm. ^13^C{^1^H} NMR (100 MHz, CDCl_3_): δ 154.3, 152.1, 117.4, 114.7, 79.9, 71.8, 58.2, 55.7, 50.7, 47.0, 30.2, 30.1, 28.2, 20.5, 20.5 ppm. IR (CDCl_3_): 3322, 1227, 1042, 827 cm^–1^. HRMS (ESI-TOF) *m*/*z*: [M + H]^+^ Calcd for C_19_H_34_NO_3_ 324.2533; Found, 324.2538.

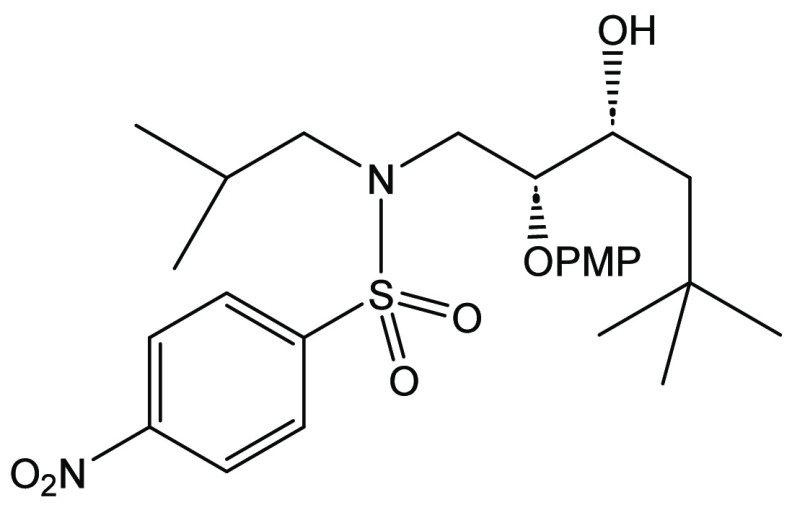



#### *N*-((2*R*,3*R*)-3-Hydroxy-2-(*p*-methoxyphenoxy)-5,5-dimethylhexyl)-*N*-isobutyl-4-nitrobenzene sulfonamide (**43**)^29^

To a stirred solution of compound **42** (5.20 g, 16.1 mmol) and dichloromethane (54 mL) in a round-bottom flask was added *p*-nitrobenzenesulfonyl chloride (8.91 g, 40.2 mmol), followed by DMAP (2.16 g, 17.7 mmol). Once all the reaction components had dissolved, triethylamine (8.80 mL, 63.1 mmol) was then added dropwise via an addition funnel. The reaction mixture was then allowed to stir overnight at room temperature. After completion, the reaction mixture was diluted with dichloromethane and treated with an aqueous solution of 1 M HCl (2 × 30 mL). The organic layer was treated with brine (30 mL), dried over MgSO_4_, and concentrated under reduced pressure to afford a crude yellow viscous oil. The residue was purified by flash column chromatography on silica gel (3:7, diethyl ether:hexanes) to afford **43** (4.132 g, 50% yield) as a yellow viscous oil. ^1^H NMR (500 MHz, CDCl_3_): δ 8.25 (d, *J* = 9.0 Hz, 2H), 7.93 (d, *J* = 9.0 Hz, 2H), 6.81–6.77 (m, 4H), 4.35 (td, *J* = 6.3, 2.7 Hz, 1H), 3.95–3.90 (m, 1H), 3.77 (s, 3H), 3.55 (dd, *J* = 15.1, 5.8 Hz, 1H), 3.25 (dd, *J* = 15.1, 6.8 Hz, 1H), 3.05 (dd, *J* = 13.6, 7.6 Hz, 1H), 2.96 (dd, *J* = 13.6, 7.5 Hz, 1H), 2.06–1.95 (m, 1H), 1.84 (d, *J* = 7.6 Hz, 1H), 1.47–1.45 (m, 2H), 0.95 (s, 9H), 0.89 (d, *J* = 6.7 Hz, 3H), 0.86 (d, *J* = 6.7 Hz, 3H)ppm. ^13^C{^1^H} NMR (100 MHz, CDCl_3_): δ 154.5, 151.6, 149.9, 144.9, 128.5, 124.2, 117.0, 114.7, 80.7, 67.9, 57.9, 55.7, 48.5, 46.7, 30.2, 30.0, 26.6, 20.0, 19.9 ppm. IR (CDCl_3_): 3542, 3106, 1606, 1531, 1350, 1226, 1160, 855 cm^–1^. HRMS (ESI-TOF) *m*/*z*: [M + Na]^+^ Calcd for C_25_H_37_N_2_O_7_S 509.2316; Found, 509.2309.

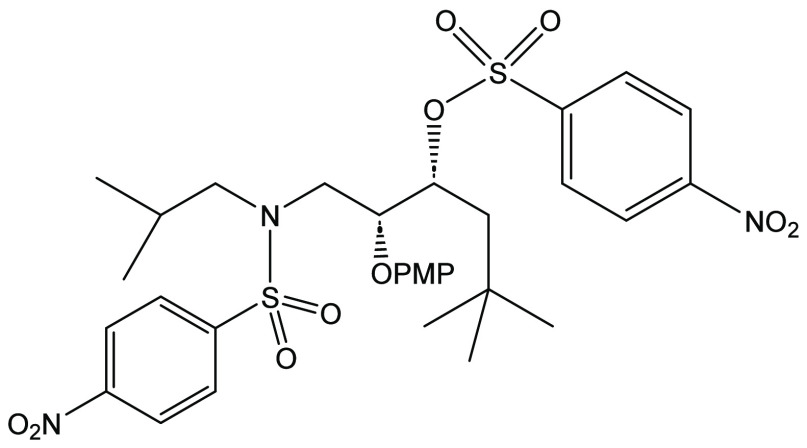



#### (2*R*,3*R*)-1-(*N*-Isobutyl-*N*-*p*-nitrophenylsulfonamido)-2-(*p*-methoxyphenoxy)-5,5-dimethyl-3-hexyl*-p*-nitrobenzenesulfonate (**44**)^29^

To a stirred solution of sulfonamide **43** (3.76 g, 7.40 mmol) and dichloromethane (25 mL) in a flame-dried, nitrogen-purged round-bottom flask was added 4-nitrobenzenesulfonyl chloride (1.97 g, 8.89 mmol), followed by DMAP (0.904 g, 7.40 mmol). Triethylamine (4.10 mL, 29.4 mmol) was then added dropwise via an addition funnel. The reaction mixture was then allowed to stir overnight at room temperature. After completion, the reaction mixture was diluted with dichloromethane and treated with an aqueous solution of 1 M HCl (2 × 30 mL). The organic layer was treated with brine (30 mL), dried over MgSO_4_, and concentrated under reduced pressure to afford a crude yellow viscous oil. The residue was purified by flash column chromatography on silica gel (7:3, diethyl ether: hexanes) to afford **44** (3.95 g, 5.70 mmol, 77% yield) as a yellow viscous oil. ^1^H NMR (500 MHz, CDCl_3_): δ 8.38 (d, *J* = 9.0 Hz, 2H), 8.12 (dd, *J* = 9.0, 5.0 Hz, 4H), 7.92 (d, *J* = 9.0 Hz, 2H), 7.50 (d, *J* = 9.0 Hz, 2H), 6.71 (d, *J* = 9.1 Hz, 2H), 6.59 (d, *J* = 9.1 Hz, 2H), 4.95 (ddd, *J* = 9.0, 4.1, 1.1 Hz, 1H), 4.77 (ddd, *J* = 9.7, 4.1, 1.5 Hz, 2H), 3.75 (s, 3H), 3.72 (d, *J* = 15.1, 1.4 Hz, 1H), 3.51 (dd, *J* = 15.5, 9.7 Hz, 1H), 3.28 (dd, *J* = 13.7, 8.3 Hz, 1H), 3.12 (dd, *J* = 13.7, 6.8 Hz, 1H), 2.10–2.02 (m, 1H), 1.71 (dd, *J* = 15.3, 1.1 Hz, 1H), 1.43 (dd, *J* = 15.3, 9.2 Hz, 1H), 0.93 (dd, *J* = 6.7, 1.8 Hz, 1H), 0.74 (s, 9H) ppm. ^13^C{^1^H} NMR (100 MHz, CDCl_3_): δ 154.8, 150.9, 149.6, 149.5, 146.3, 142.0, 129.3, 128.0, 124.5, 124.1, 116.2, 114.7, 77.7, 75.4, 56.2, 55.6, 46.2, 40.6, 29.9, 29.3, 26.4, 19.8, 19.7 ppm. IR (CDCl_3_): 3108, 1607, 1532, 1350, 829 cm^–1^. HRMS (ESI-TOF) *m*/*z*: [M + H]^+^ Calcd for C_31_H_42_N_3_O_9_S_2_ 694.2099; Found, 694.2090.

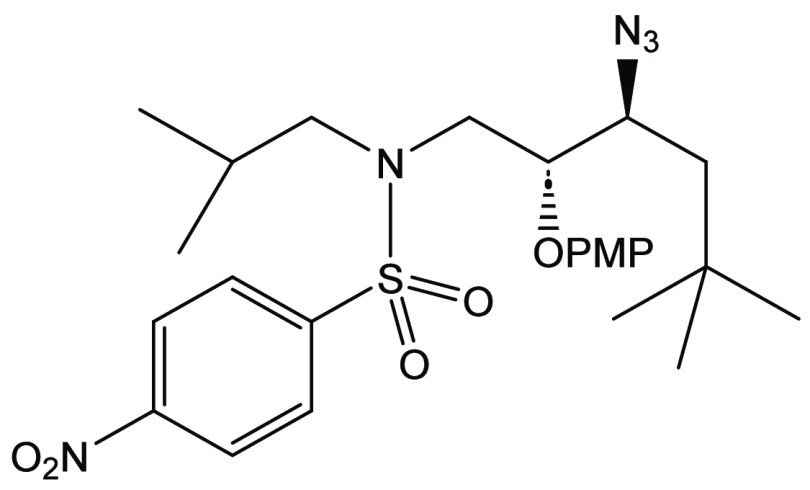



#### *N*-((2*R*,3*S*)-3-Azido-2-(*p*-methoxyphenoxy)-5,5-dimethylhexyl)-*N*-isobutyl-4-nitrobenzene sulfonamide (**45**)^29^

To a stirring solution of compound **44** (3.73 g, 5.37 mmol) in DMSO (36 mL) was added sodium azide (1.40 g). The reaction mixture was allowed to stir overnight at room temperature. The reaction mixture was diluted with diethyl ether (100 mL) and washed with an aqueous solution of 1 M HCl (2 × 30 mL). The organic layer was washed one more time with brine, dried over MgSO_4_, and concentrated under reduced pressure. The residue was purified by flash column chromatography eluting with dichloromethane to afford azide **45** (2.615 g, 91% yield) as a yellow viscous oil. ^1^H NMR (500 MHz, CDCl_3_): δ 8.24 (d, *J* = 8.9 Hz, 2H), 7.95 (d, *J* = 8.9 Hz, 6.77 (d, *J* = 9.2 Hz, 2H), 6.69 (d, *J* = 9.2 Hz, 2H), 4.41 (dt, *J* = 9.3, 2.4 Hz, 2H), 3.76 (s, 3H), 3.71 (dt, *J* = 8.1, 2.6 Hz, 1H), 3.56 (dd, *J* = 15.6, 2.0 Hz, 1H), 3.41 (dd, *J* = 15.6, 9.1 Hz, 1H), 3.25 (dd, *J* = 13.5, 8.6 Hz, 1H), 2.97 (dd, *J* = 13.5, 6.5 Hz, 1H), 2.09–2.01 (m, 1H), 1.33 (dd, *J* = 14.5, 2.7 Hz, 1H), 1.29 (dd, *J* = 14.5, 8.1 Hz, 1H), 0.93 (s, 9H), 0.89 (d, *J* = 6.6 Hz, 3H), 0.85 (d, *J* = 6.6 Hz, 3H) ppm. ^13^C{^1^H} NMR (100 MHz, CDCl_3_): δ 154.8, 149.9, 149.8, 145.7, 128.2, 124.2, 116.9, 114.9, 81.7, 59.4, 57.5, 55.6, 48.1, 43.8, 30.4, 29.4, 26.6, 20.0, 19.9 ppm. IR (CDCl_3_): 3106, 2115, 1606, 1531, 1350, 827 cm^–1^. HRMS (ESI-TOF) *m*/*z*: [M + Na]^+^ Calcd for C_25_H_35_N_5_NaO_6_S 556.2200; Found, 556.2205.

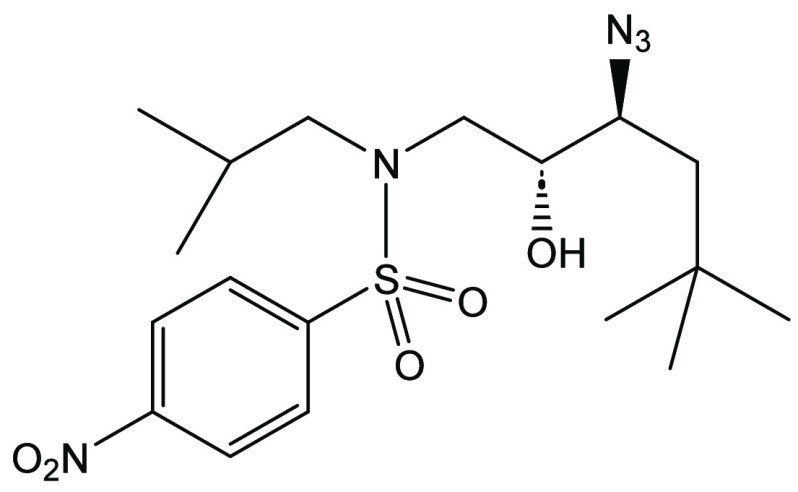



#### *N*-((2*R*,3*S*)-3-Azido-2-hydroxy-5,5-dimethylhexyl)-*N*-isobutyl-*p*-nitrobenzenesulfonamide (**46**)^29^

To a stirred solution of **45** (2.12 g, 3.96 mmol) in CH_3_CN (46.5 mL) was added cerium (IV) ammonium nitrate (4.35 g, 7.93 mmol), followed by deionized water (15.5 mL). The reaction mixture was allowed to stir at room temperature for 1 h. The reaction mixture was then diluted with diethyl ether (100 mL), washed with brine (2 × 30 mL), dried over MgSO_4_, and concentrated under reduced pressure to afford a dark-red viscous oil. The residue was purified by flash column chromatography on silica gel (2:8, diethyl ether: hexane) to afford azide **46** (0.6406 g, 38% yield) as a dark-red viscous oil. ^1^H NMR (500 MHz, CDCl_3_): δ 8.39 (d, *J* = 8.9 Hz, 2H), 8.02 (d, *J* = 8.9 Hz, 2H), 3.81 (broad singlet, 1H), 3.39 (ddd, *J* = 9.1, 4.8, 2.0 Hz, 1H), 3.33 (t, *J* = 15.3, 9.5 Hz, 1H), 3.13–3.04 (m, 3H), 2.97 (dd, *J* = 13.6, 7.0 Hz, 1H), 1.95–1.86 (m, 1H), 1.44 (dd, *J* = 14.6, 2.0 Hz, 1H), 1.31 (dd, *J* = 14.6, 9.2 Hz, 1H), 0.98 (s, 9H), 0.95 (d, *J* = 6.7 Hz, 3H), 0.90 (d, *J* = 6.7 Hz, 3H) ppm. ^13^C{^1^H} NMR (100 MHz, CDCl_3_): δ 150.2, 144.6, 128.6, 124.5, 73.7, 62.7, 58.2, 51.7, 43.3, 30.1, 29.6, 27.1, 20.1, 19.8 ppm. IR (CDCl_3_): 3524, 2115, 1531,1350, 1159, 1089, 856 cm^–1^. HRMS (ESI-TOF) *m*/*z*: [M + Na]^+^ Calcd for C_18_H_29_N_5_NaO_5_S 450.1782; Found, 450.1786.

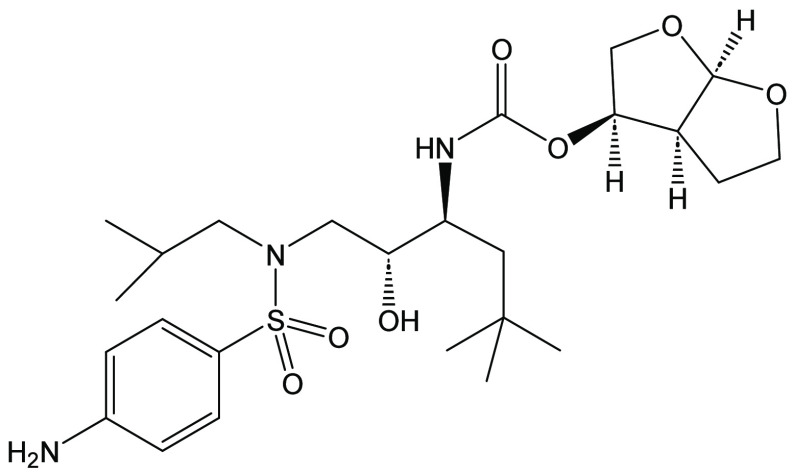



#### (3*R*,3*aS*,6*aR*)-Hexahydrofuro[2,3-*b*]furan-3-yl((2*R*,3*S*)-1-(-*N*-*p*-aminophenyl-*N*-isobutyl sulfonamido)-2-hydroxy-5,5-dimethyl-3-hexyl)carbamate (**38**)^29^

A flame-dried 250 mL round-bottom flask containing compound **46** (0.424 g, 0.992 mmol), 5% palladium on carbon (0.005 g), 2,5-dioxopyrrolidin-1-yl((3*R*,3*aS*,6*aR*)-hexahydrofuro[2,3-*b*]furan-3-yl)carbonate (0.242 g, 0.890 mmol), and THF (33 mL) was flushed with nitrogen gas for 10 min. A balloon filled with hydrogen gas was then connected to the reaction flask, and the mixture was allowed to stir overnight at room temperature. The reaction mixture was diluted with chloroform, filtered through a 1:1 mixture of Celite and MgSO_4_, and concentrated under reduced pressure. The crude residue was purified by flash column chromatography on silica gel with diethyl ether as the mobile phase to afford the desired **38** (0.4139 g, 0.784 mmol, 79% yield) as a pale-yellow viscous oil. [α]_D_ = −19.3 (*c* = 1.00, CDCl_3_). ^1^H NMR (500 MHz, CDCl_3_): δ 7.56 (d, *J* = 8.8 Hz, 2H), 6.69 (d, *J* = 8.8 Hz, 2H), 5.71 (d, *J* = 5.2 Hz, 1H), 5.12–5.16 (m, 1H), 5.01 (d, *J* = 9.3 Hz, 1H), 4.20 (br.s, 2H), 4.28 (dd, *J* = 9.5, 6.4 Hz, 1H), 3.94 (d, *J* = 8.3, 2.75 Hz, 1H), 3.85–3.90 (m, 1H), 3.74 (d, *J* = 9.5, 6.7 Hz, 2H), 3.59–3.65 (m, 1H), 3.37 (d, *J* = 3.0 Hz, 1H), 3.13–3.02 (m, 2H), 2.96–2.90 (m, 2H), 2.81 (d, *J* = 13.4, 6.9 Hz, 1H), 2.02–1.97 (m, IH), 1.89–1.80 (m, 2H), 1.57 (d, *J* = 14.6 Hz, 1H), 1.32–1.25 (m, 1H), 9.33 (s, 9H), 0.92 (d, *J* = 6.5 Hz, 3H), 0.89 (d, *J* = 6.5 Hz, 3H) ppm. ^13^C{^1^H} NMR (125 MHz, CDCl_3_): δ 155.3, 151.5, 129.3, 125.3, 113.9, 109.2, 74.0, 73.2, 70.7, 69.4, 58.2, 52.9, 51.8, 45.2, 432.0, 30.1, 29.7, 27.0, 25.8, 20.1, 20.0 ppm. IR (CDCl_3_): 3459, 3364, 3254, 1706, 1147, 1090, 831 cm^–1^. HRMS (ESI-TOF) *m*/*z*: [M + H]^+^ Calcd for C_25_H_42_N_3_O_7_S 528.2738; Found, 528.2743.

## Data Availability

The data underlying this study are available in the published article and its Supporting Information.

## Supporting Information Available

^1^H and ^13^C NMR spectroscopic data for all reported compounds. X-ray crystallographic data for (*R*,*S*,*R*)-**10** are provided (PDF)
