# Supplementary material for: Diastereoselective Synthesis of the HIV Protease Inhibitor Darunavir and Related Derivatives via a Titanium Tetrachloride-Mediated Asymmetric Glycolate Aldol Addition Reaction
Source: J Org Chem. 2024 Jun 25;89(13):9569–85. doi: 10.1021/acs.joc.4c01057 (PMC11232028; doi:10.1021/acs.joc.4c01057)
Supplement: Supplementary file 1 — jo4c01057_si_001.pdf [file jo4c01057_si_001.pdf]

Supporting Information

Diastereoselective synthesis of the HIV protease inhibitor darunavir and related derivatives via a titanium tetrachloride mediated asymmetric glycolate aldol addition reaction

Jordan M. Witte, Emmanuel Ayim, Christopher J. Sams, Jasmine B. Service, Caitlyn C. Kant, Lillian Bambalas, Daniel Wright, Austin Carter, Kelly Moran, Isabella G. Rohrig, Gregory M. Ferrence, and Shawn R. Hitchcock*

Department of Chemistry, Illinois State University, Normal, IL 617090-4160

Table of Contents

400 MHz ^{1}H NMR spectrum of (*S*)-**17**	5
100 MHz ^{13}C{^{1}H} NMR spectrum of (*S*)-**17**	6
400 MHz ^{1}H NMR spectrum of (*R*)-**17**	7
100 MHz ^{13}C{^{1}H} NMR spectrum of (*R*)-**17**	8
400 MHz ^{1}H NMR spectrum of (*S*)-**11**	9
100 MHz ^{13}C{^{1}H} NMR spectrum of (*S*)-**11**	10
400 MHz ^{1}H NMR spectrum of (*R*)-**11**	11
100 MHz ^{13}C{^{1}H} NMR spectrum of (*R*)-**11**	12
500 MHz ^{1}H NMR spectrum of (*S*,*S*,*R*)-**10**	13
125 MHz ^{13}C{^{1}H} NMR spectrum of (*S*,*S*,*R*)-**10**	14
400 MHz ^{1}H NMR spectrum of (*R*,*S*,*R*)-**10**	15
100 MHz ^{13}C{^{1}H} NMR spectrum of (*R*,*S*,*R*)-**10**	16
500 MHz ^{1}H NMR spectrum of **15**	17
125 MHz ^{13}C{^{1}H} NMR spectrum of **15**	18
400 MHz ^{1}H NMR spectrum of **18**	19
100 MHz ^{13}C{^{1}H} NMR spectrum of **18**	20
400 MHz ^{1}H NMR spectrum of **14**	21
100 MHz ^{13}C{^{1}H} NMR spectrum of **14**	22
400 MHz ^{1}H NMR spectrum of **19**	23
100 MHz ^{13}C{^{1}H} NMR spectrum of **19**	24
500 MHz ^{1}H NMR spectrum of **13**	25

100 MHz $^{13}C\{^{1}H\}$ NMR spectrum of **13**	26
400 MHz ^{1}H NMR spectrum of **20**	27
100 MHz $^{13}C\{^{1}H\}$ NMR spectrum of **20**	28
500 MHz ^{1}H NMR spectrum of **21**	29
100 MHz $^{13}C\{^{1}H\}$ NMR spectrum of **21**	30
500 MHz ^{1}H NMR spectrum of **22**	31
100 MHz $^{13}C\{^{1}H\}$ NMR spectrum of **22**	32
500 MHz ^{1}H NMR spectrum of **12**	33
100 MHz $^{13}C\{^{1}H\}$ NMR spectrum of **12**	34
400 MHz ^{1}H NMR spectrum of **23**	35
100 MHz $^{13}C\{^{1}H\}$ NMR spectrum of **23**	36
500 MHz ^{1}H NMR spectrum of **1**	37
125 MHz $^{13}C\{^{1}H\}$ NMR spectrum of **1**	38
500 MHz ^{1}H NMR spectrum of **27**	39
100 MHz $^{13}C\{^{1}H\}$ NMR spectrum of **27**	40
500 MHz ^{1}H NMR spectrum of **28**	41
100 MHz $^{13}C\{^{1}H\}$ NMR spectrum of **28**	42
500 MHz ^{1}H NMR spectrum of **30**	43
100 MHz $^{13}C\{^{1}H\}$ NMR spectrum of **30**	44
500 MHz ^{1}H NMR spectrum of **31**	45
100 MHz $^{13}C\{^{1}H\}$ NMR spectrum of **31**	46
500 MHz ^{1}H NMR spectrum of **32**	47
100 MHz $^{13}C\{^{1}H\}$ NMR spectrum of **32**	48

500 MHz ^{1}H NMR spectrum of **33**	49
100 MHz ^{13}C{^{1}H} NMR spectrum of **33**	50
500 MHz ^{1}H NMR spectrum of **34**	51
100 MHz ^{13}C{^{1}H} NMR spectrum of **34**	52
500 MHz ^{1}H NMR spectrum of **35**	53
100 MHz ^{13}C{^{1}H} NMR spectrum of **35**	54
500 MHz ^{1}H NMR spectrum of **36**	55
100 MHz ^{13}C{^{1}H} NMR spectrum of **36**	56
500 MHz ^{1}H NMR spectrum of **41**	57
125 MHz ^{13}C{^{1}H} NMR spectrum of **41**	58
500 MHz ^{1}H NMR spectrum of **42**	59
100 MHz ^{13}C{^{1}H} NMR spectrum of **42**	60
500 MHz ^{1}H NMR spectrum of **43**	61
100 MHz ^{13}C{^{1}H} NMR spectrum of **43**	62
500 MHz ^{1}H NMR spectrum of **44**	63
100 MHz ^{13}C{^{1}H} NMR spectrum of **44**	64
500 MHz ^{1}H NMR spectrum of **45**	65
100 MHz ^{13}C{^{1}H} NMR spectrum of **45**	66
500 MHz ^{1}H NMR spectrum of **46**	67
100 MHz ^{13}C{^{1}H} NMR spectrum of **46**	68
500 MHz ^{1}H NMR spectrum of **38**	69
125 MHz ^{13}C{^{1}H} NMR spectrum of **38**	70
X-ray Crystallographic Data and ORTEP for **10**	71

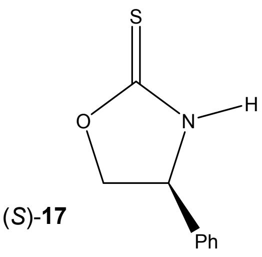

(*S*)-**17**

^{1}H NMR (500 MHz, $CDCl_3$)

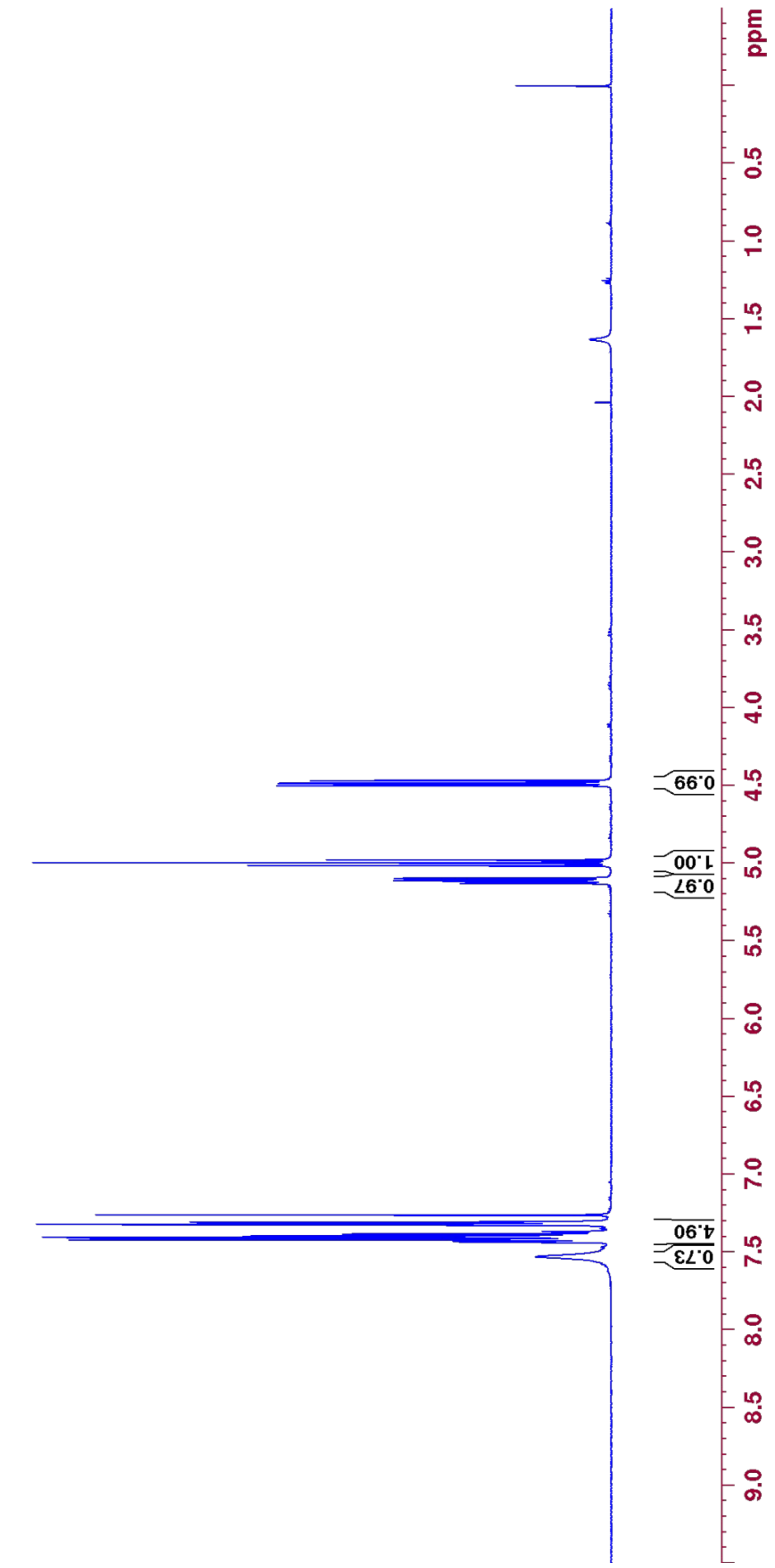

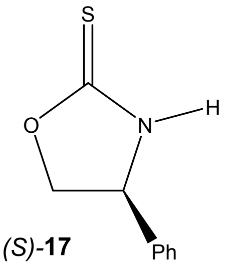

(S)-**17**

$^{13}C\{^{1}H\}$ NMR (125 MHz, $CDCl_3$)

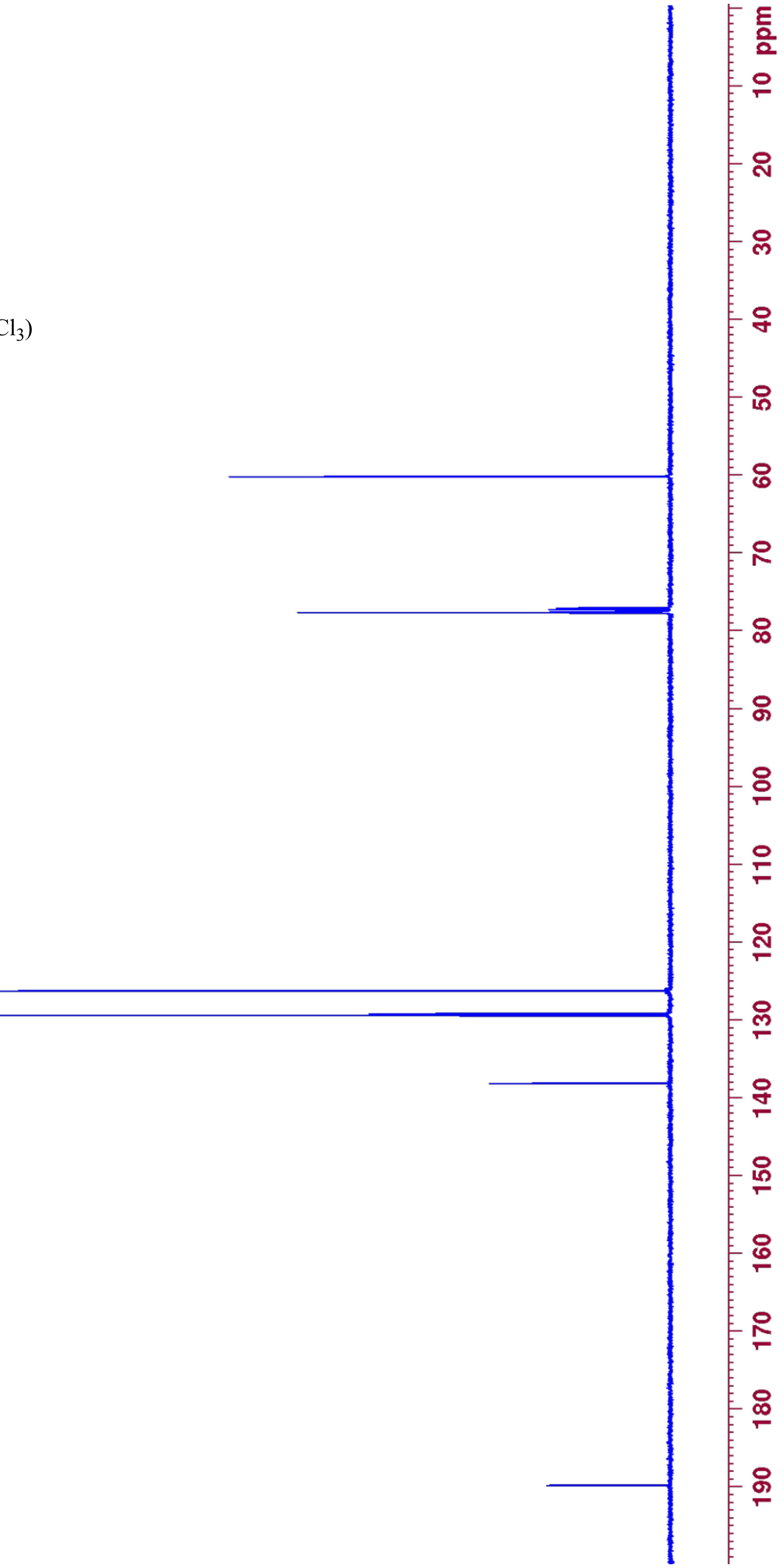

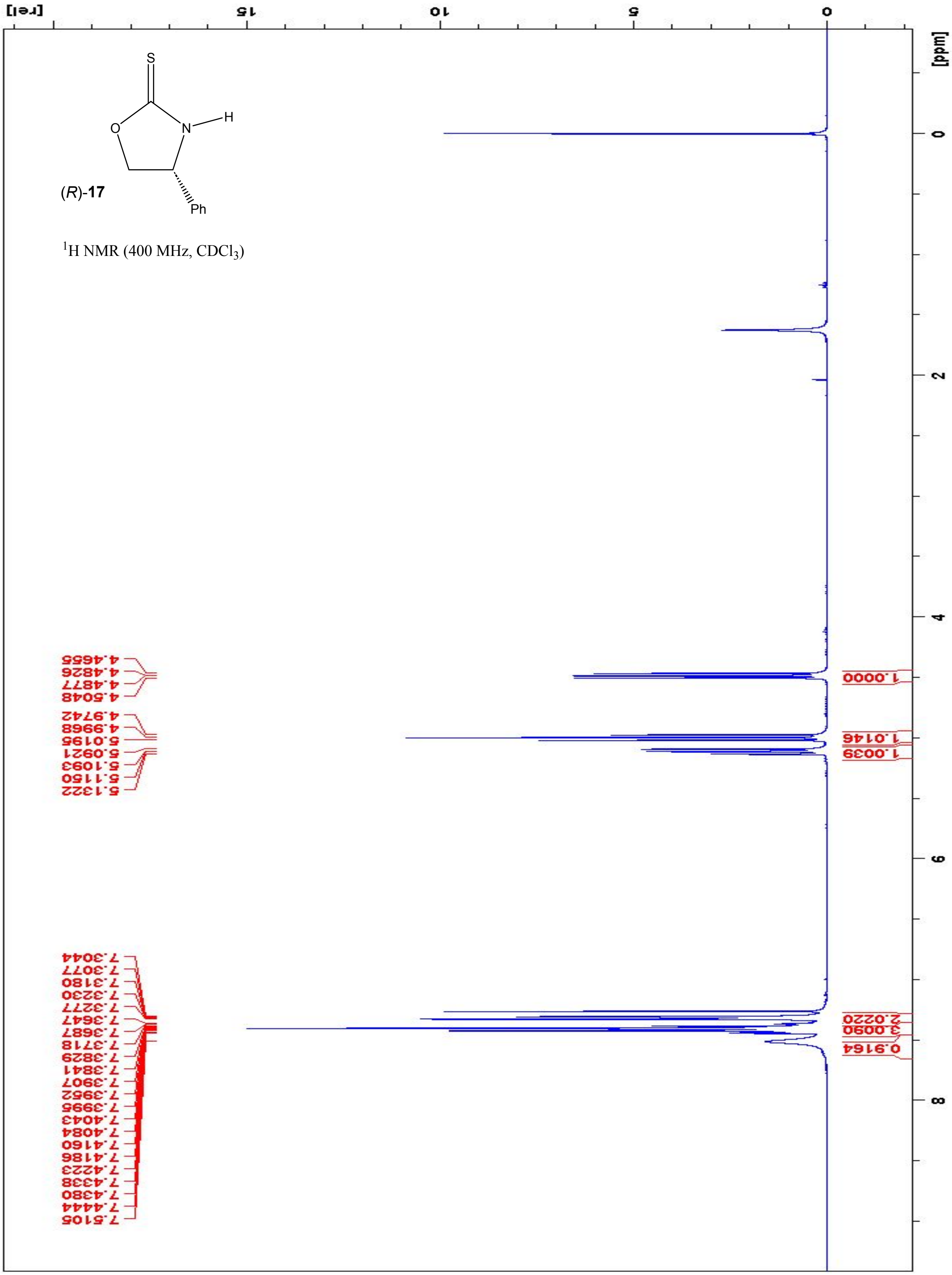

(*R*)-**17**

^1H NMR (400 MHz, $CDCl_3$)

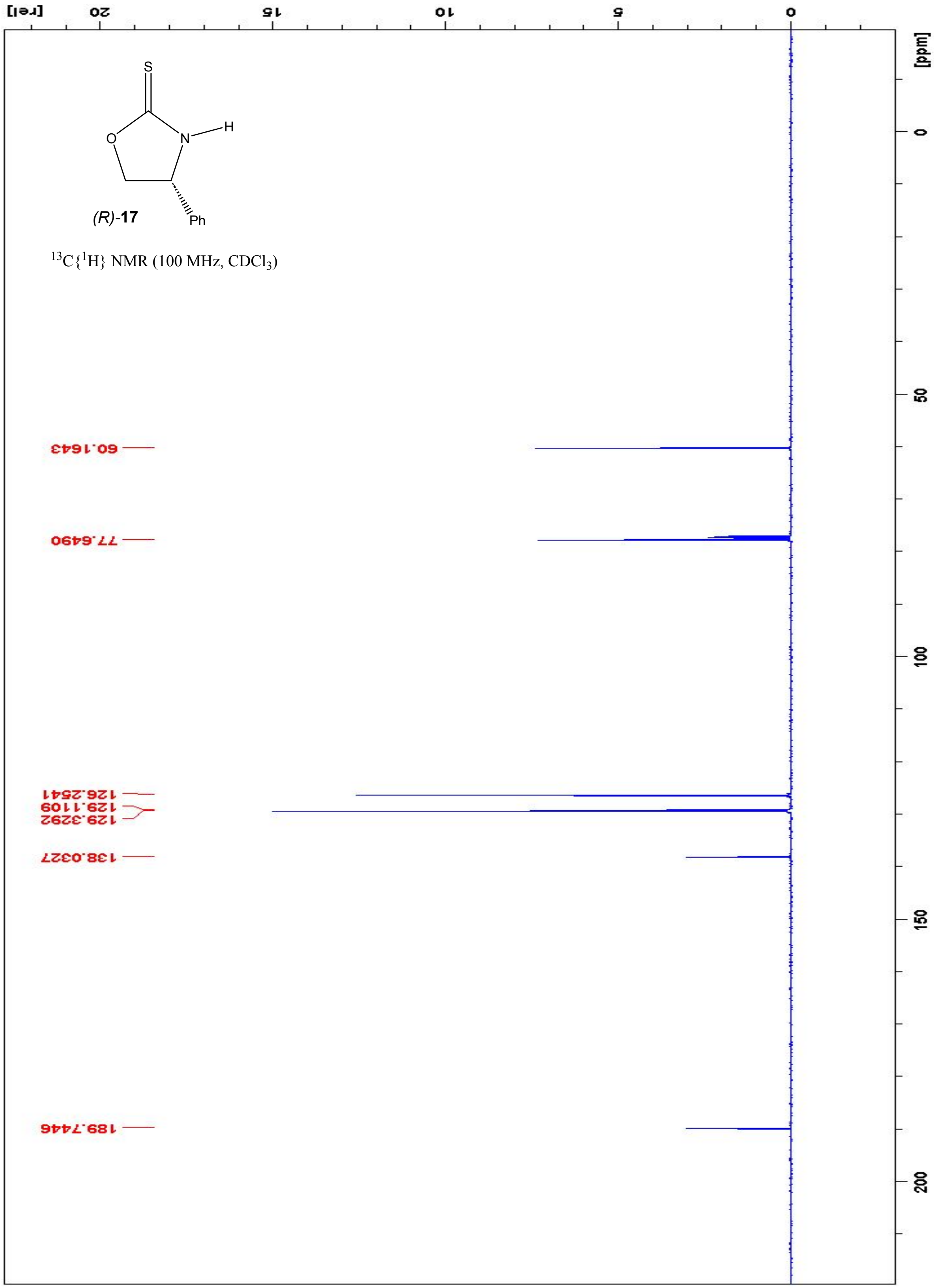
(R)-17
$^{13}C\{^{1}H\}$ NMR (100 MHz, $CDCl_3$)
60.1643
77.6490
126.2541
129.1109
129.3292
138.0327
189.7446
[ppm]
[rel]

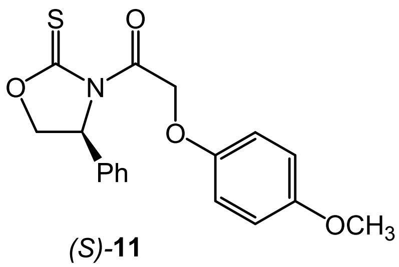

(S)-**11**

^{1}H NMR (400 MHz, $CDCl_3$)

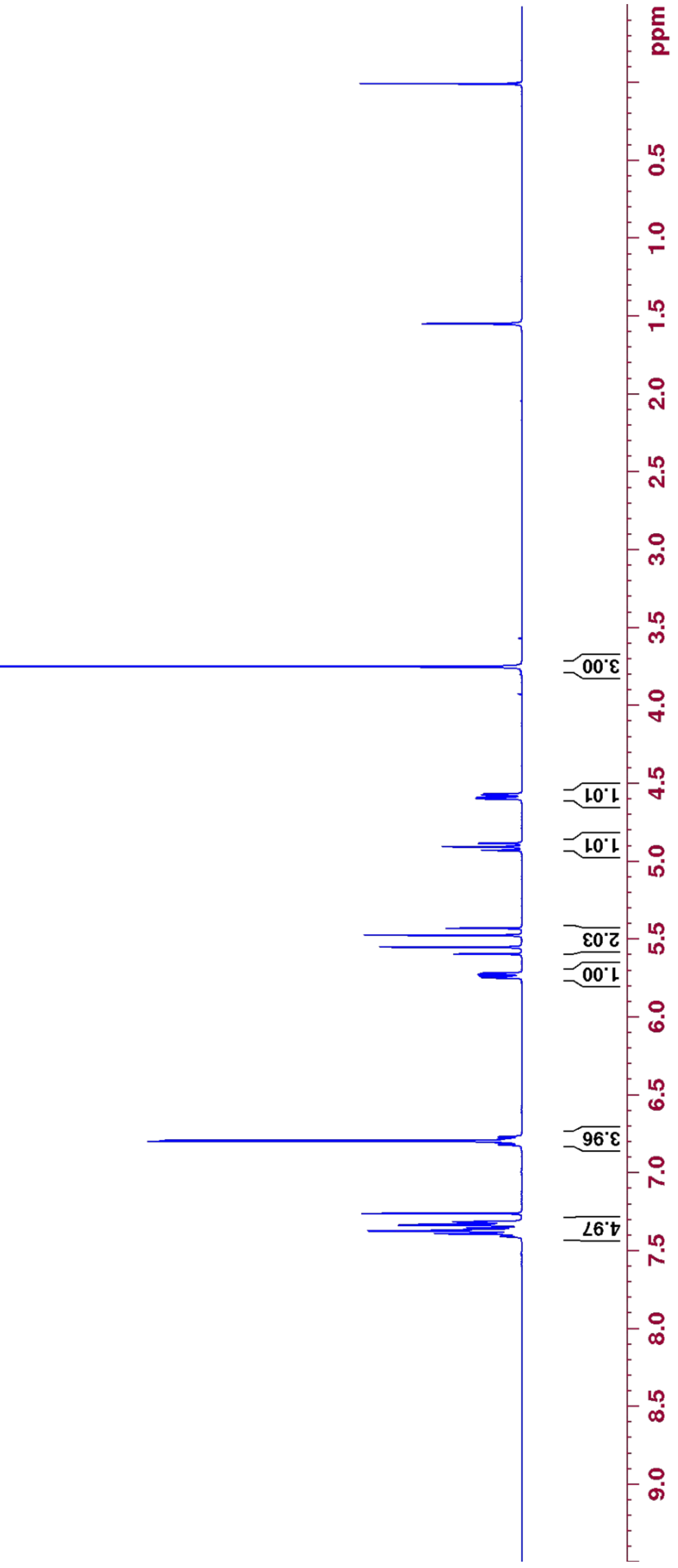

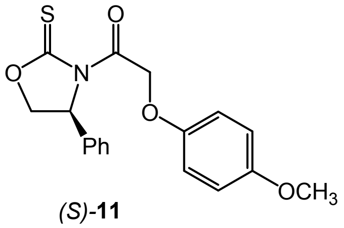

(S)-**11**

$^{13}C\{^{1}H\}$ NMR (100 MHz, $CDCl_3$)

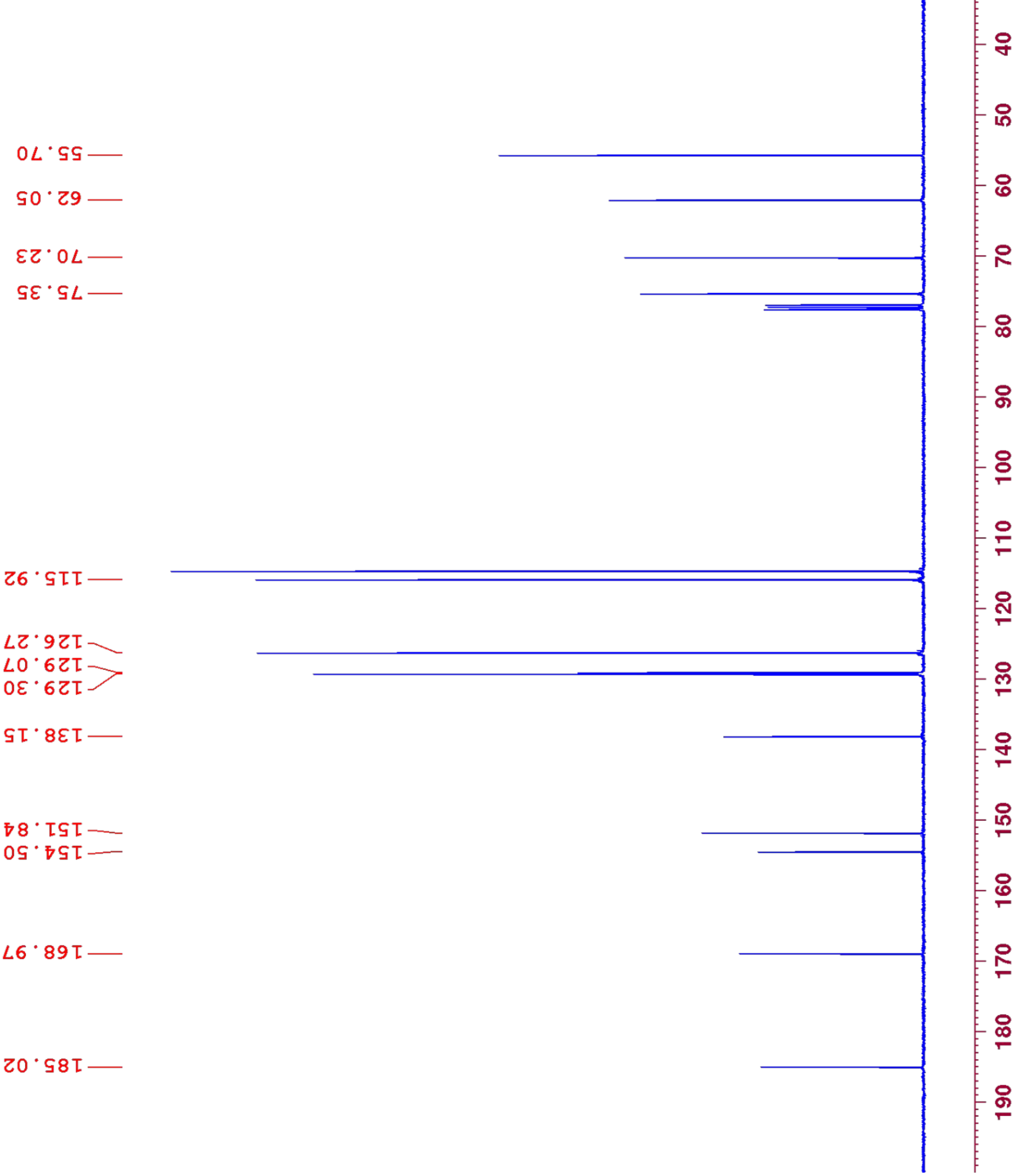

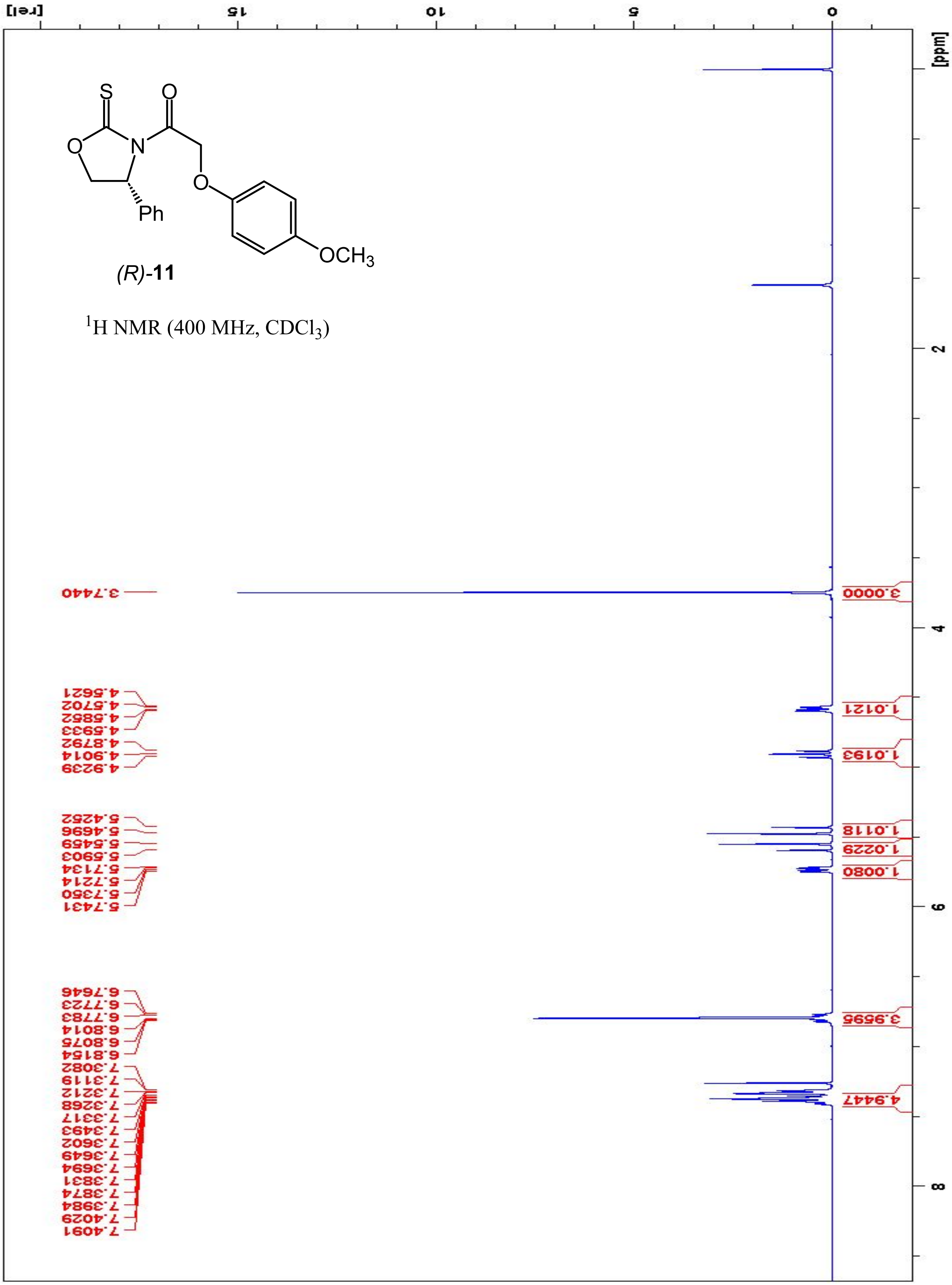
S
O
O
N
O
Ph
OCH3
(R)-11
1H NMR (400 MHz, CDCl3)
[rel]
[ppm]
3.7440
4.5621
4.5702
4.5852
4.5933
4.8792
4.9014
4.9239
5.4252
5.4696
5.5459
5.5903
5.7134
5.7214
5.7350
5.7431
6.7646
6.7723
6.7783
6.8014
6.8075
6.8154
7.3082
7.3119
7.3212
7.3268
7.3317
7.3493
7.3602
7.3649
7.3694
7.3831
7.3874
7.3984
7.4029
7.4091
3.0000
1.0121
1.0193
1.0118
1.0229
1.0080
3.9595
4.9447

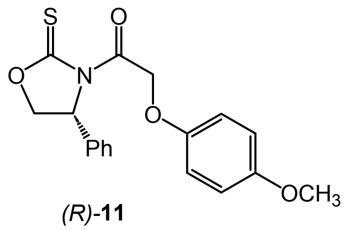

(*R*)-**11**

$^{13}C\{^1H\}$ NMR (100 MHz, $CDCl_3$)

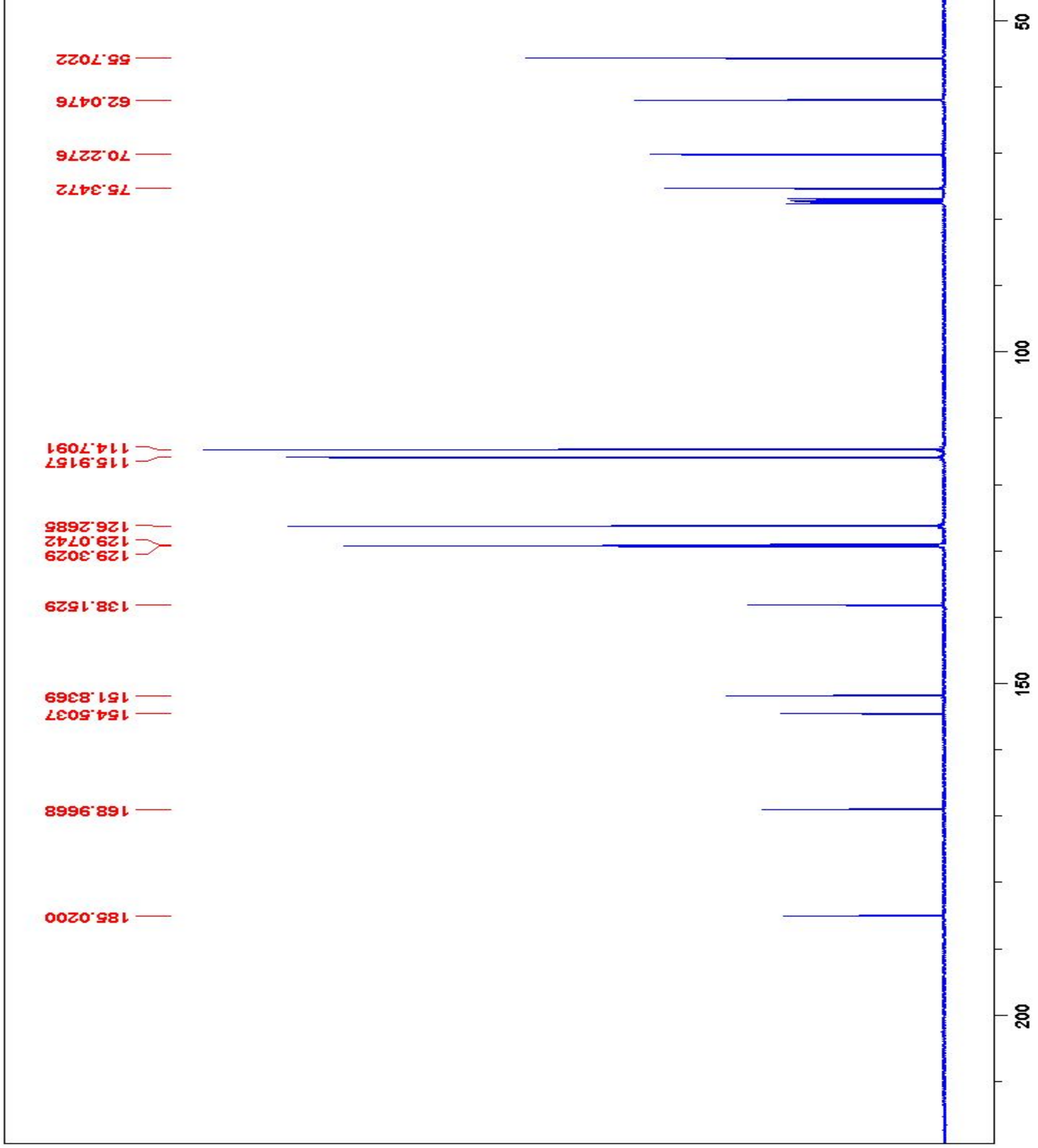

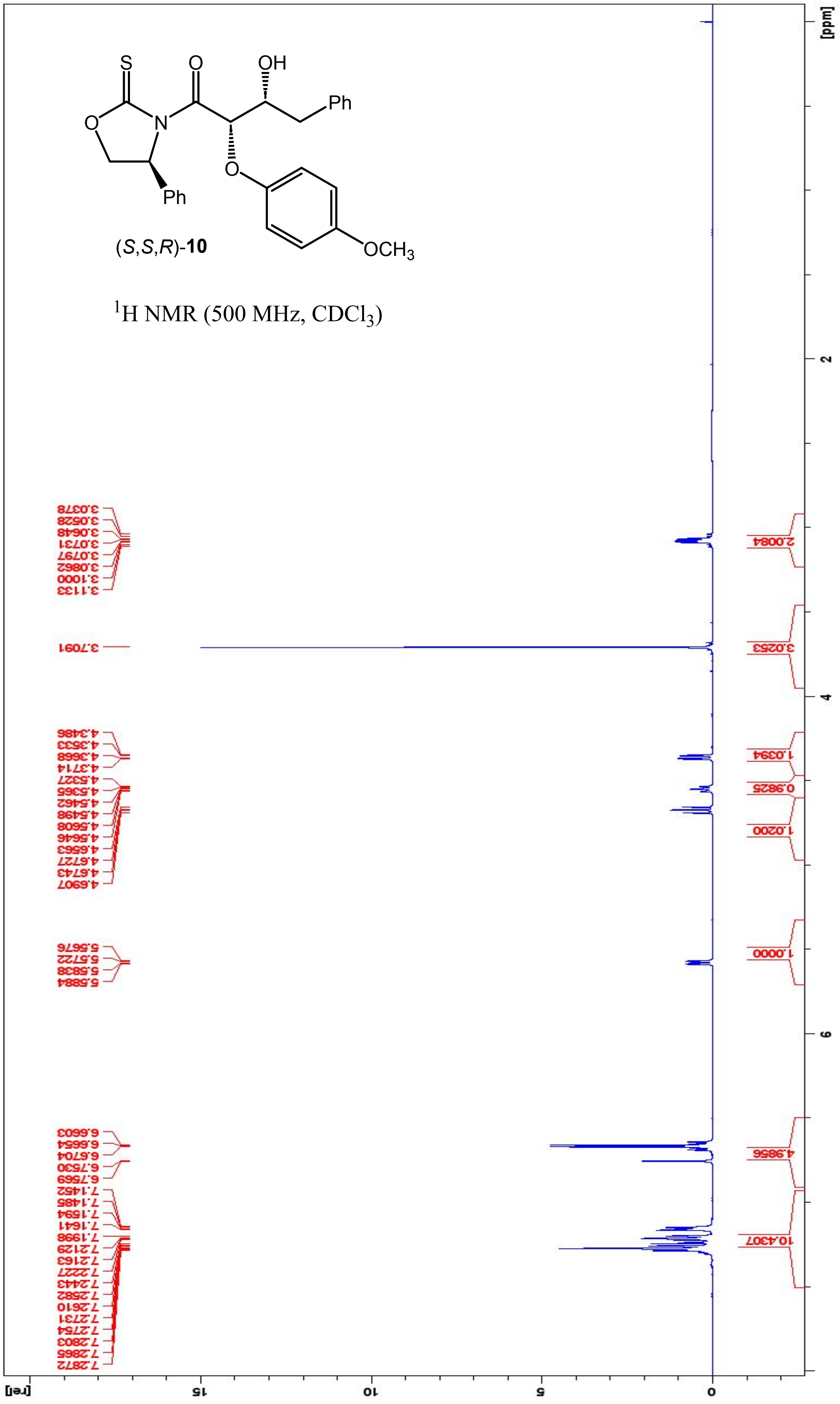
S
O
OH
Ph
O
N
O
Ph
OCH3
(S,S,R)-10
1H NMR (500 MHz, CDCl3)

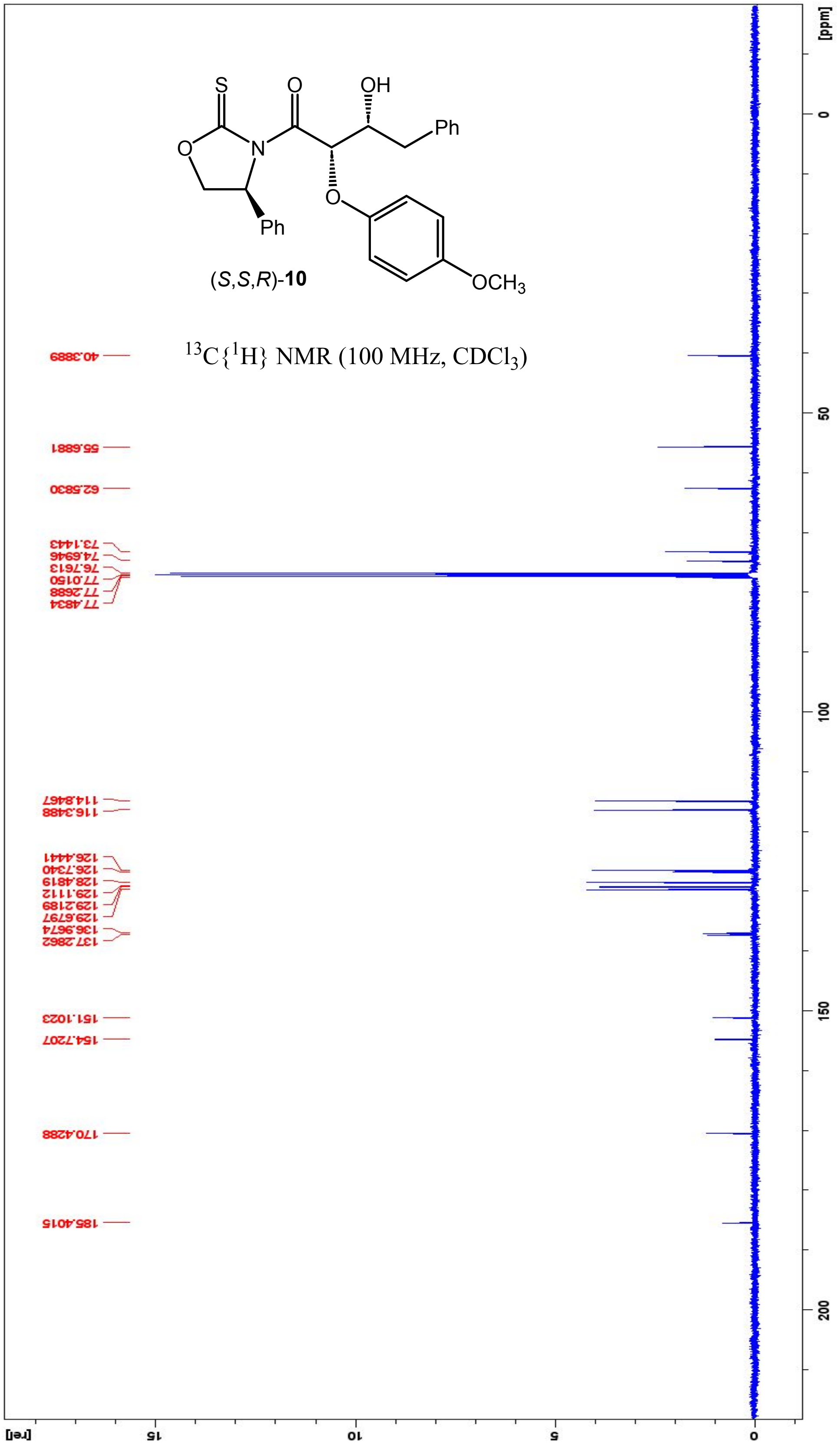

(S,S,R)-10
$^{13}C\{^{1}H\}$ NMR (100 MHz, $CDCl_3$)
40.3889
55.6881
62.5830
73.1443
74.6946
76.7613
77.0150
77.2688
77.4834
114.8467
116.3488
126.4441
126.7340
128.4819
129.1112
129.2189
129.6797
136.9674
137.2862
151.1023
154.7207
170.4288
185.4015
[ppm]
0
50
100
150
200
[rel]
0
5
10
15

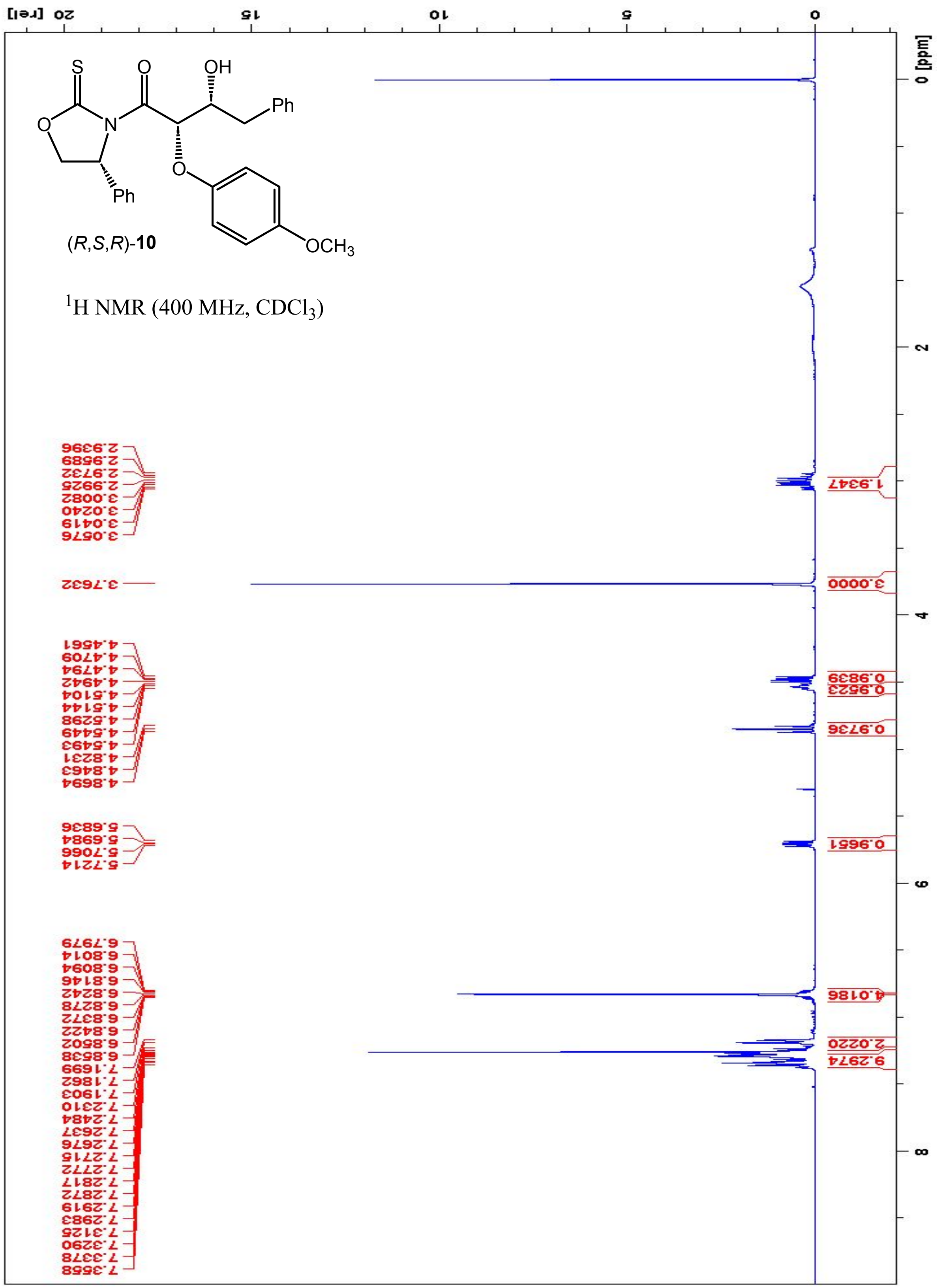
(R,S,R)-10
^{1}H NMR (400 MHz, $CDCl_3$)

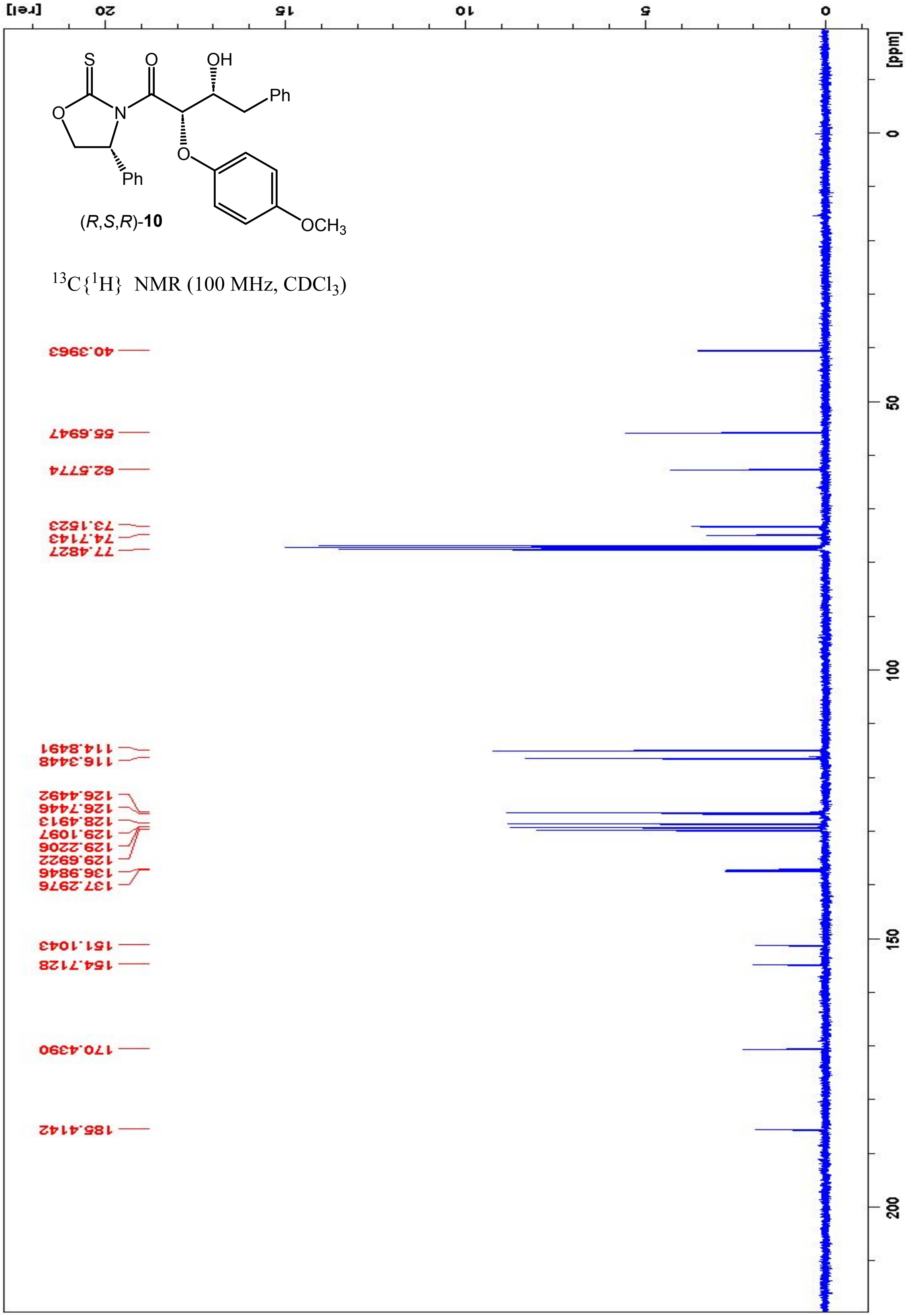
(R,S,R)-10
OCH3
13C{1H} NMR (100 MHz, CDCl3)
40.3963
55.6947
62.5774
73.1523
74.7143
77.4827
114.8491
116.3448
126.4492
126.7446
128.4913
129.1097
129.2206
129.6922
136.9846
137.2976
151.1043
154.7128
170.4390
185.4142
[ppm]
[rel]

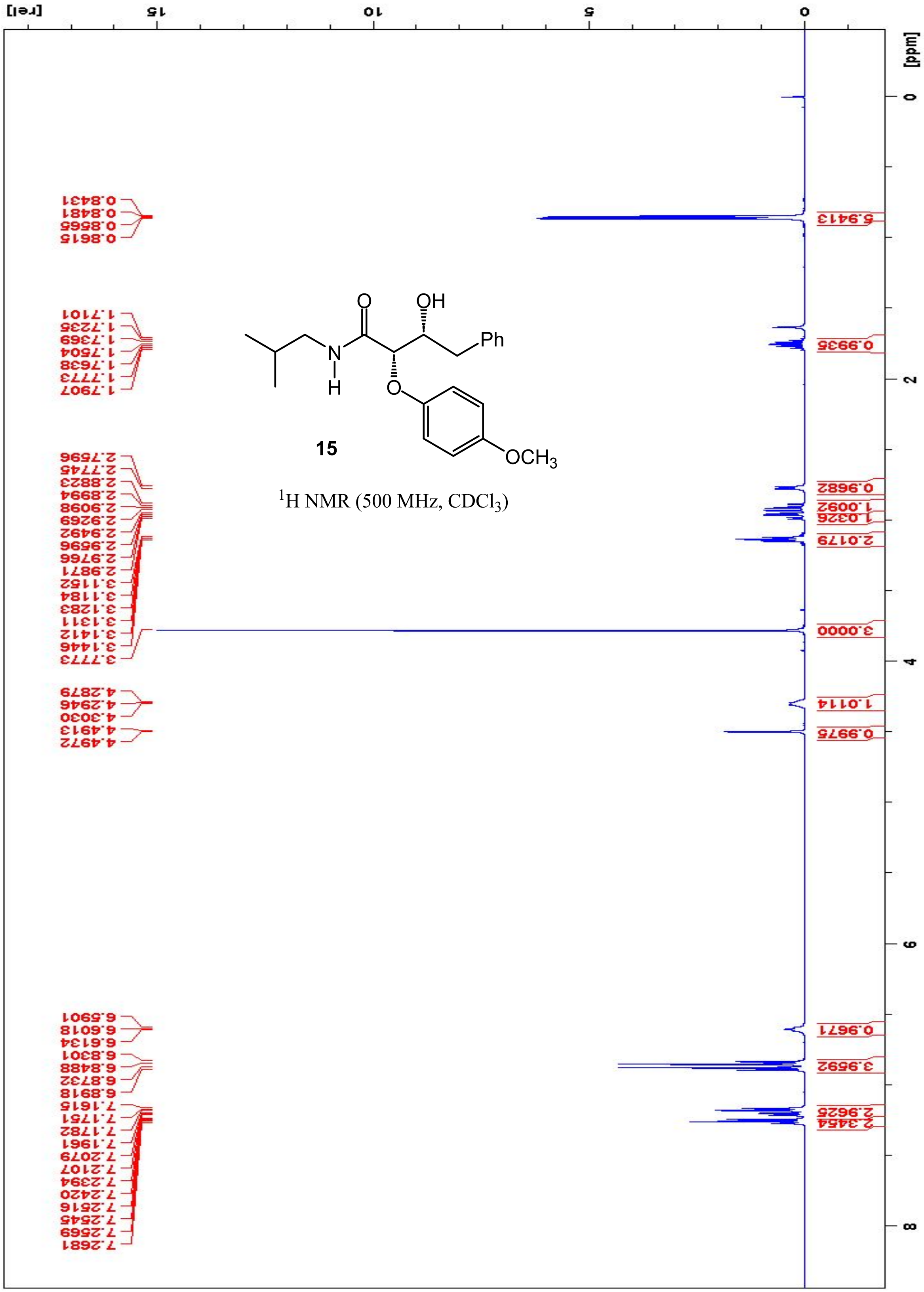

15

^{1}H NMR (500 MHz, $CDCl_3$)

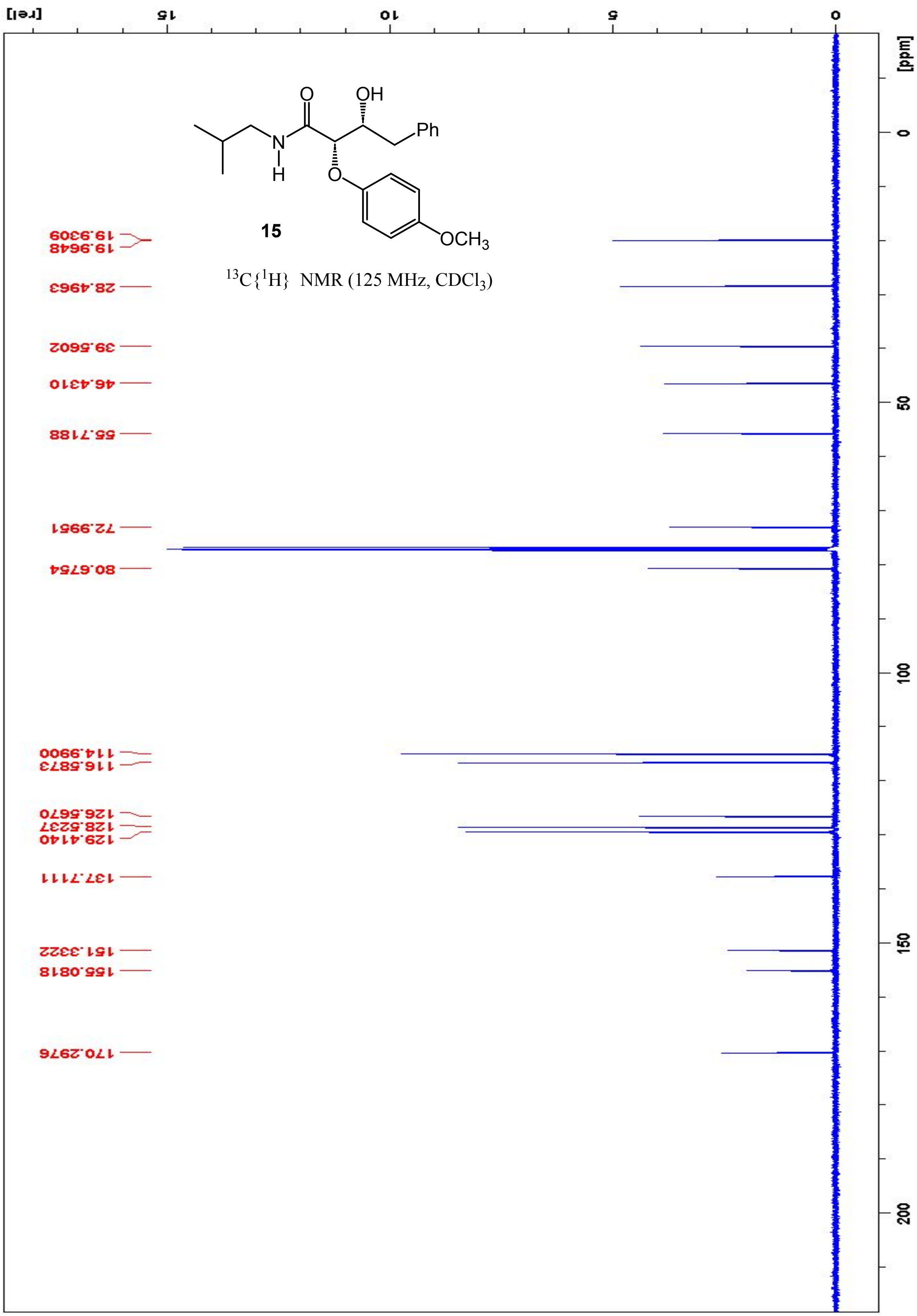

15

$^{13}C\{^{1}H\}$ NMR (125 MHz, $CDCl_3$)

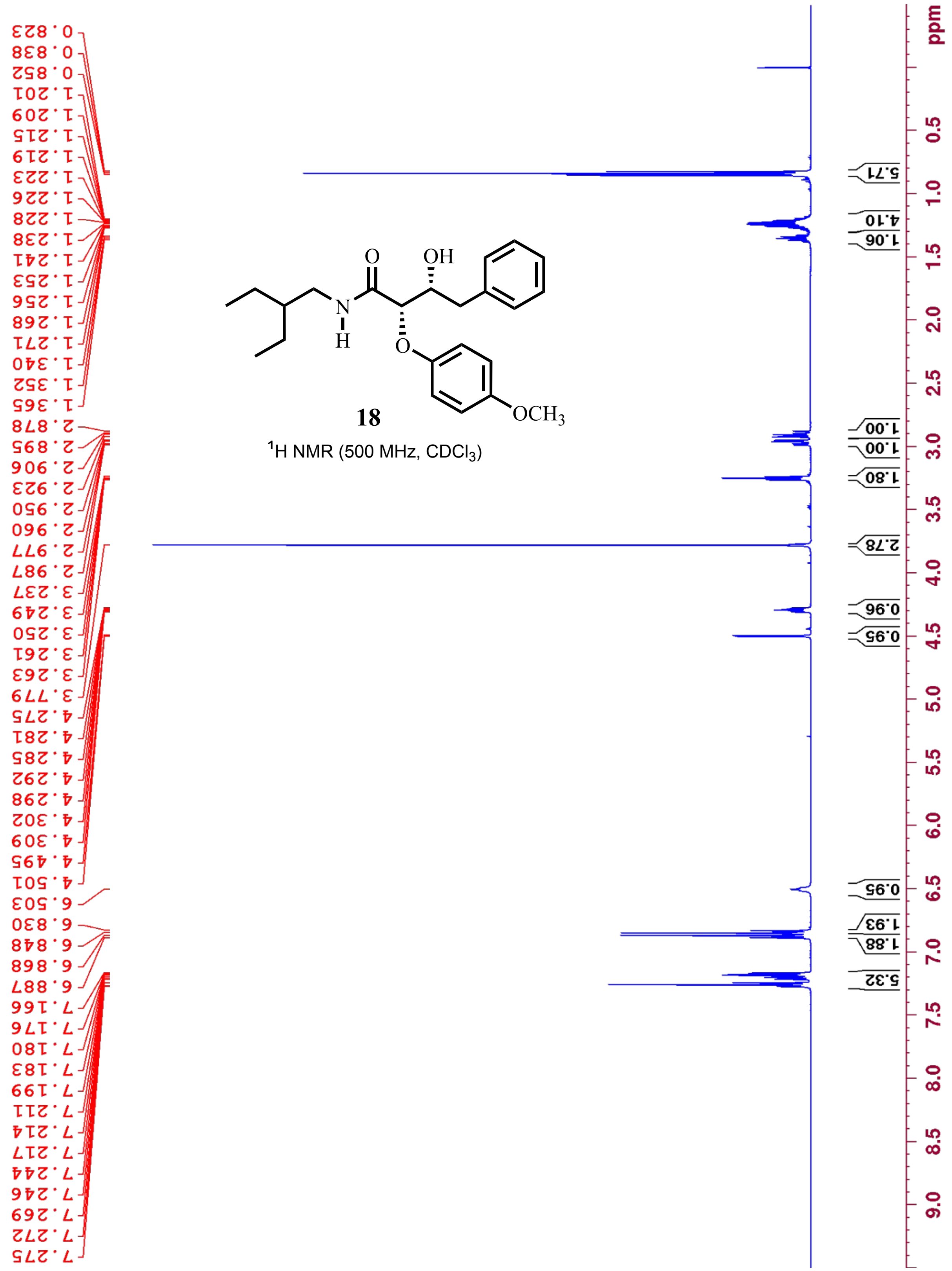

18

^{1}H NMR (500 MHz, $CDCl_3$)

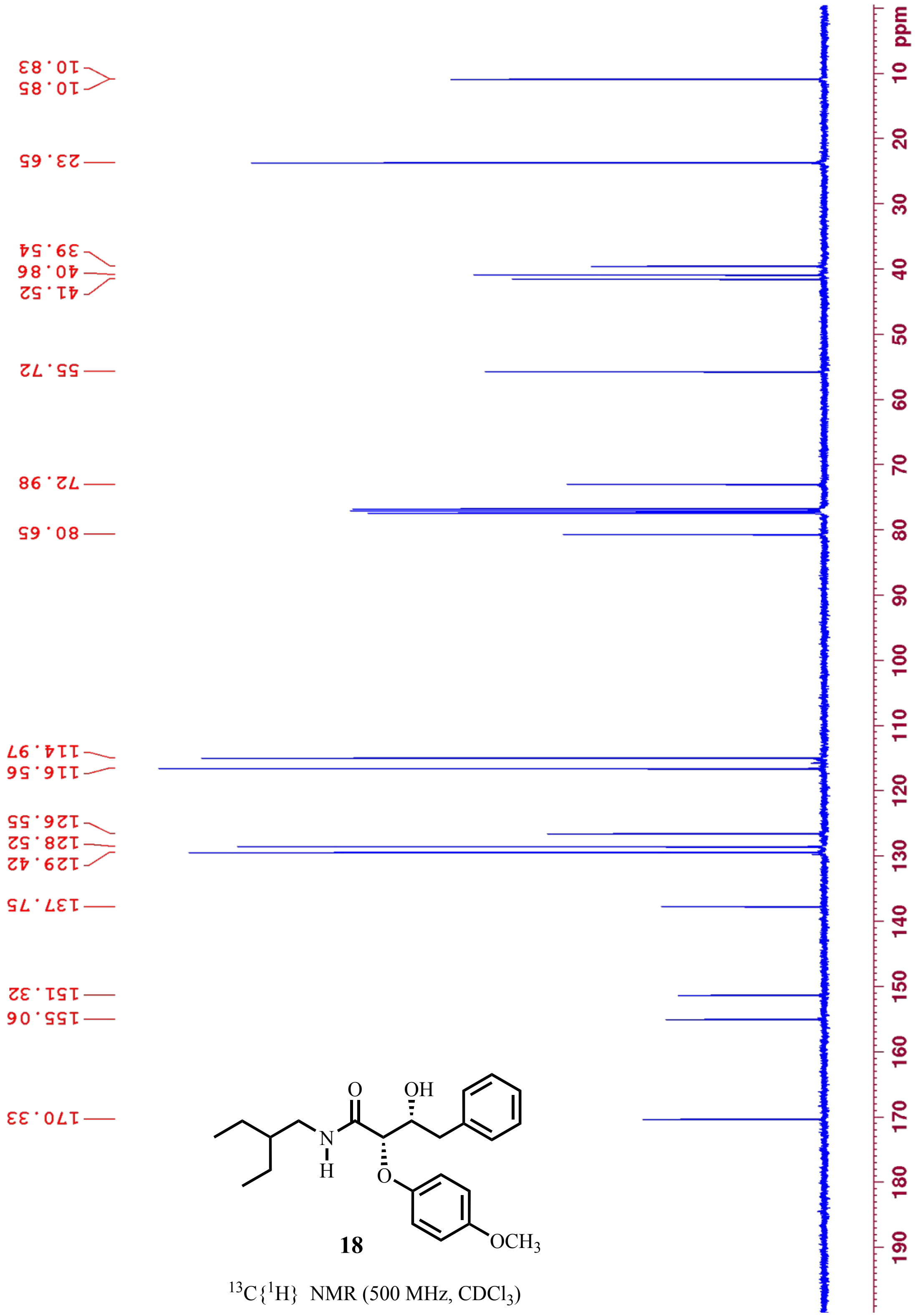

$^{13}C\{^{1}H\}$ NMR (500 MHz, $CDCl_3$)

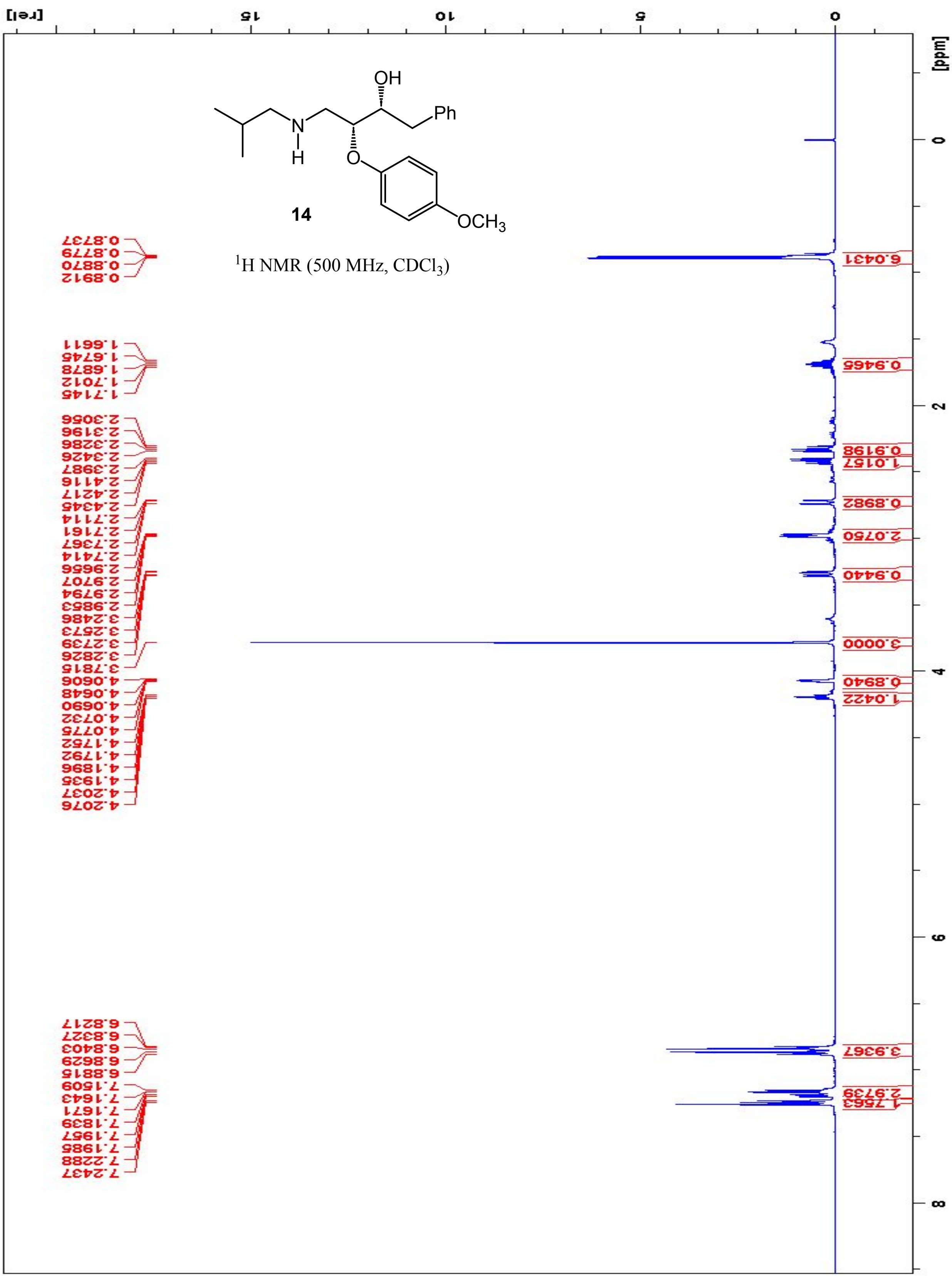

14

^{1}H NMR (500 MHz, $CDCl_3$)

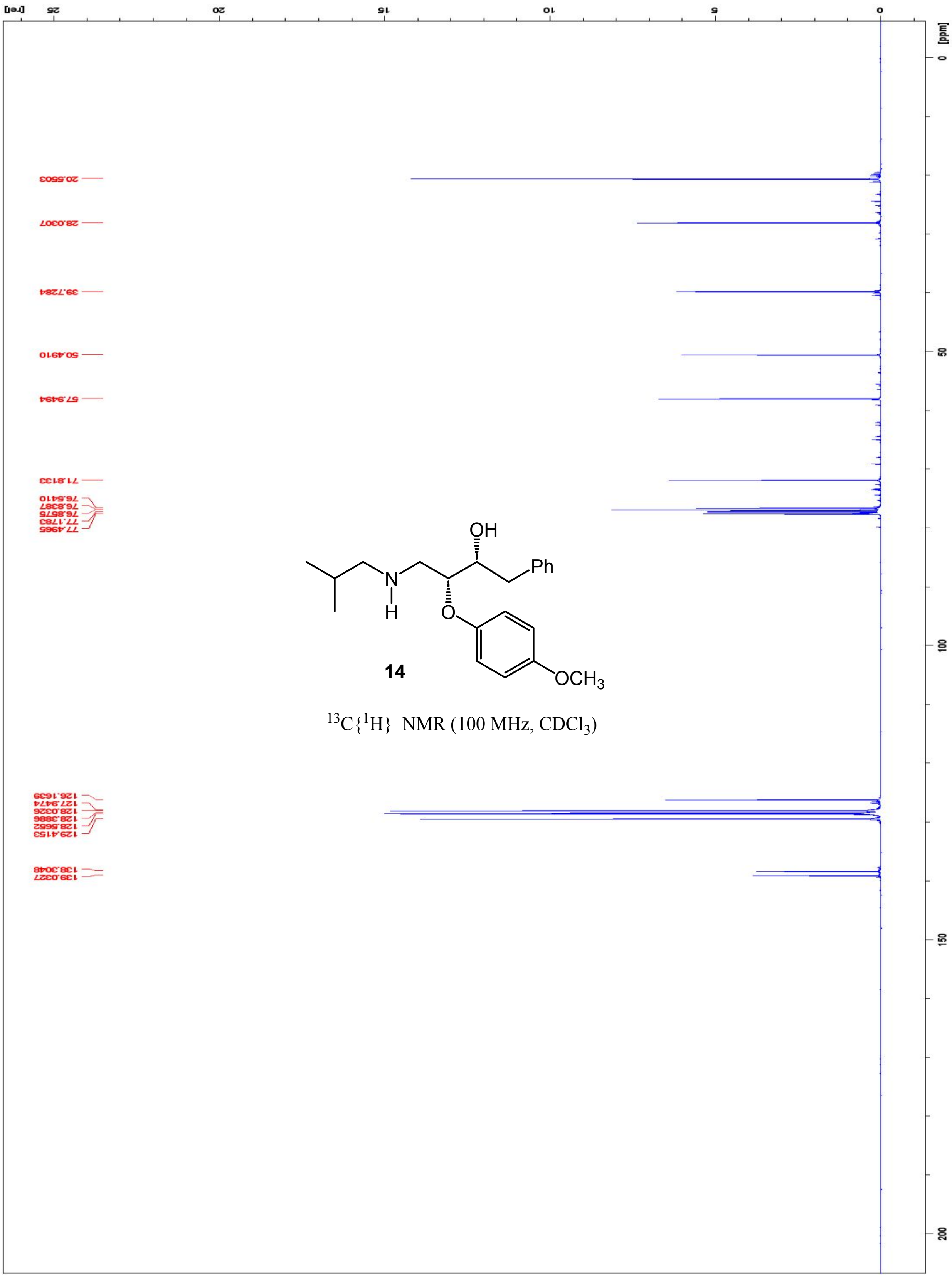

14

$^{13}C\{^{1}H\}$ NMR (100 MHz, $CDCl_3$)

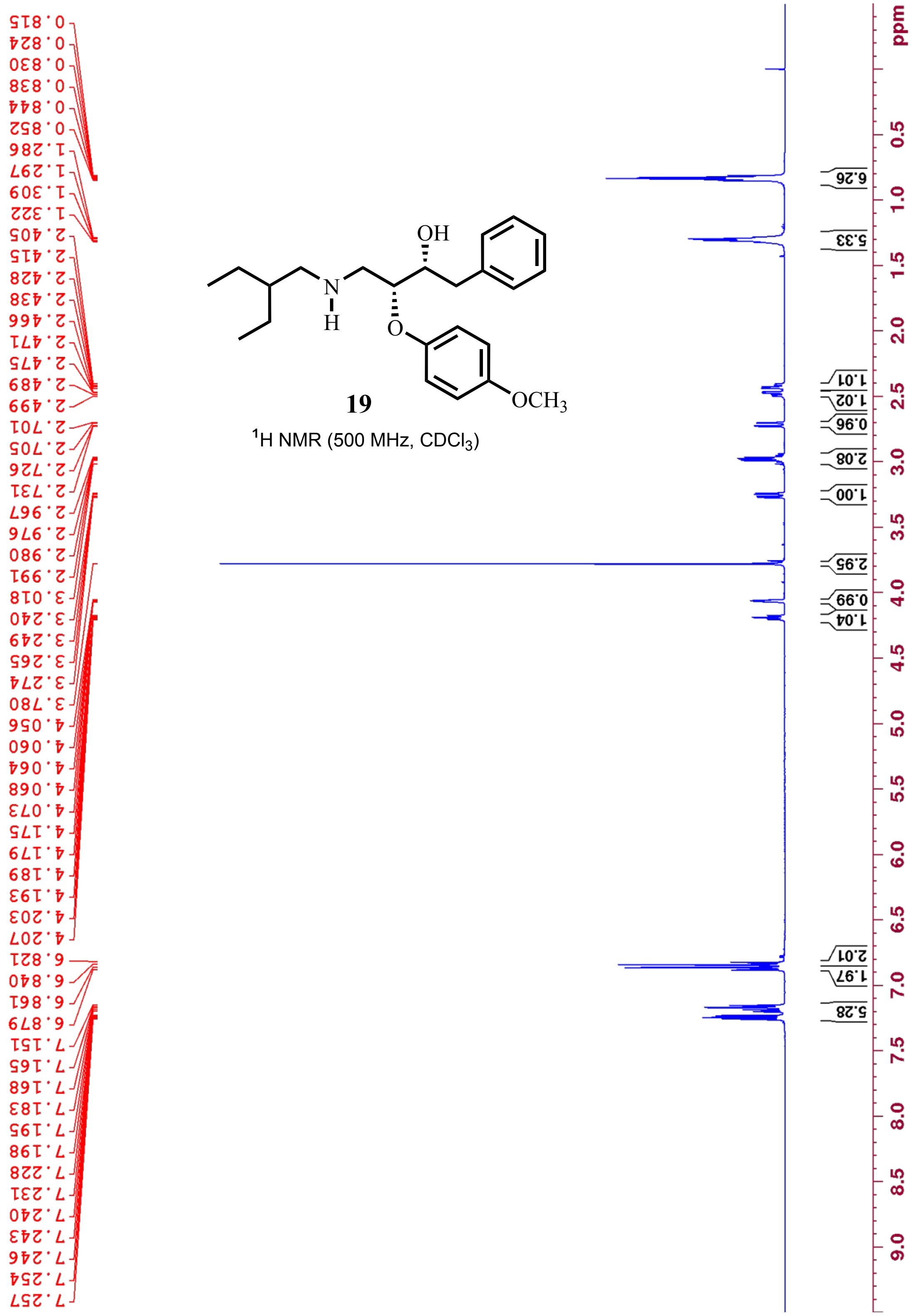

19

^{1}H NMR (500 MHz, $CDCl_3$)

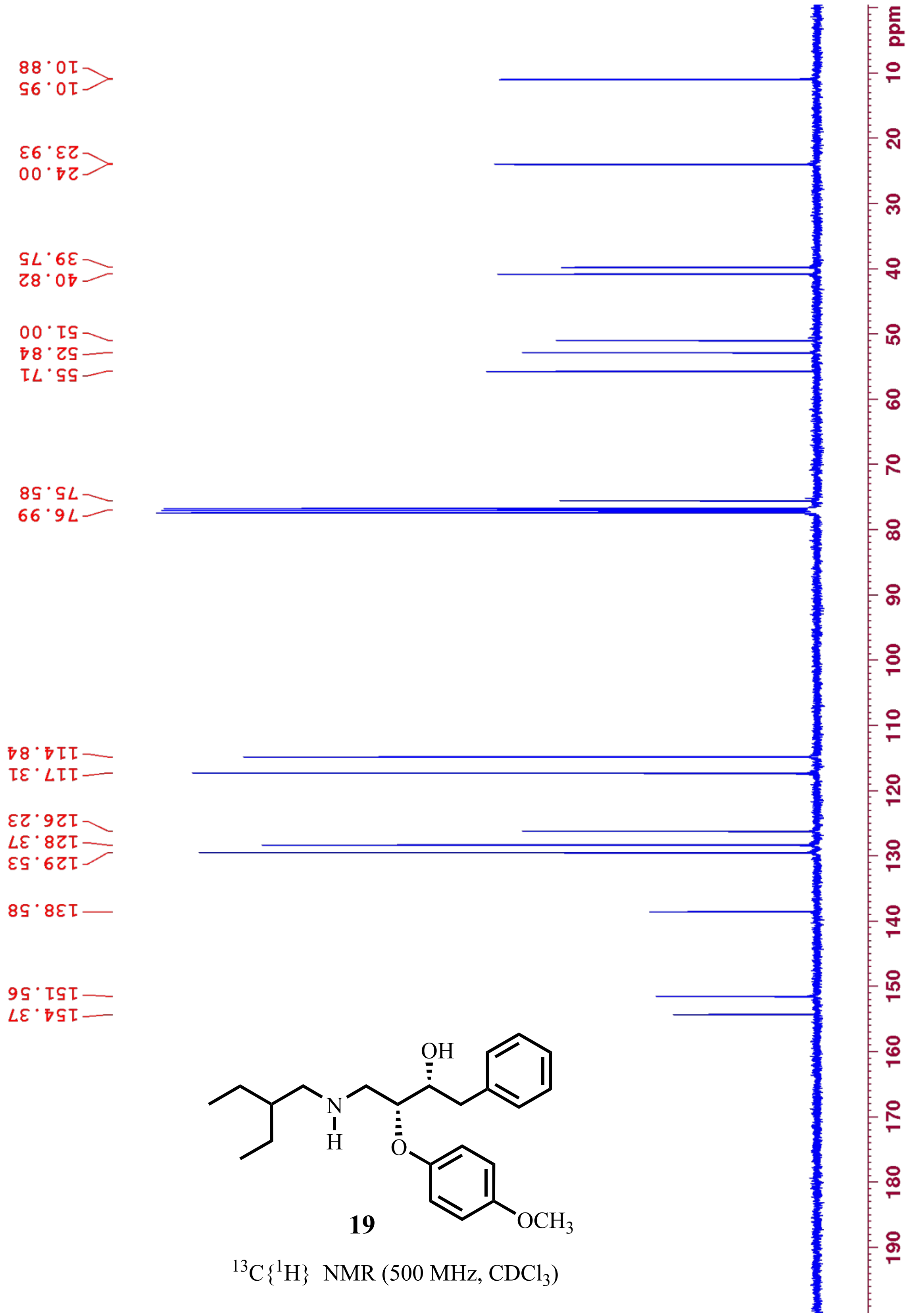

19

$^{13}C\{^{1}H\}$ NMR (500 MHz, $CDCl_3$)

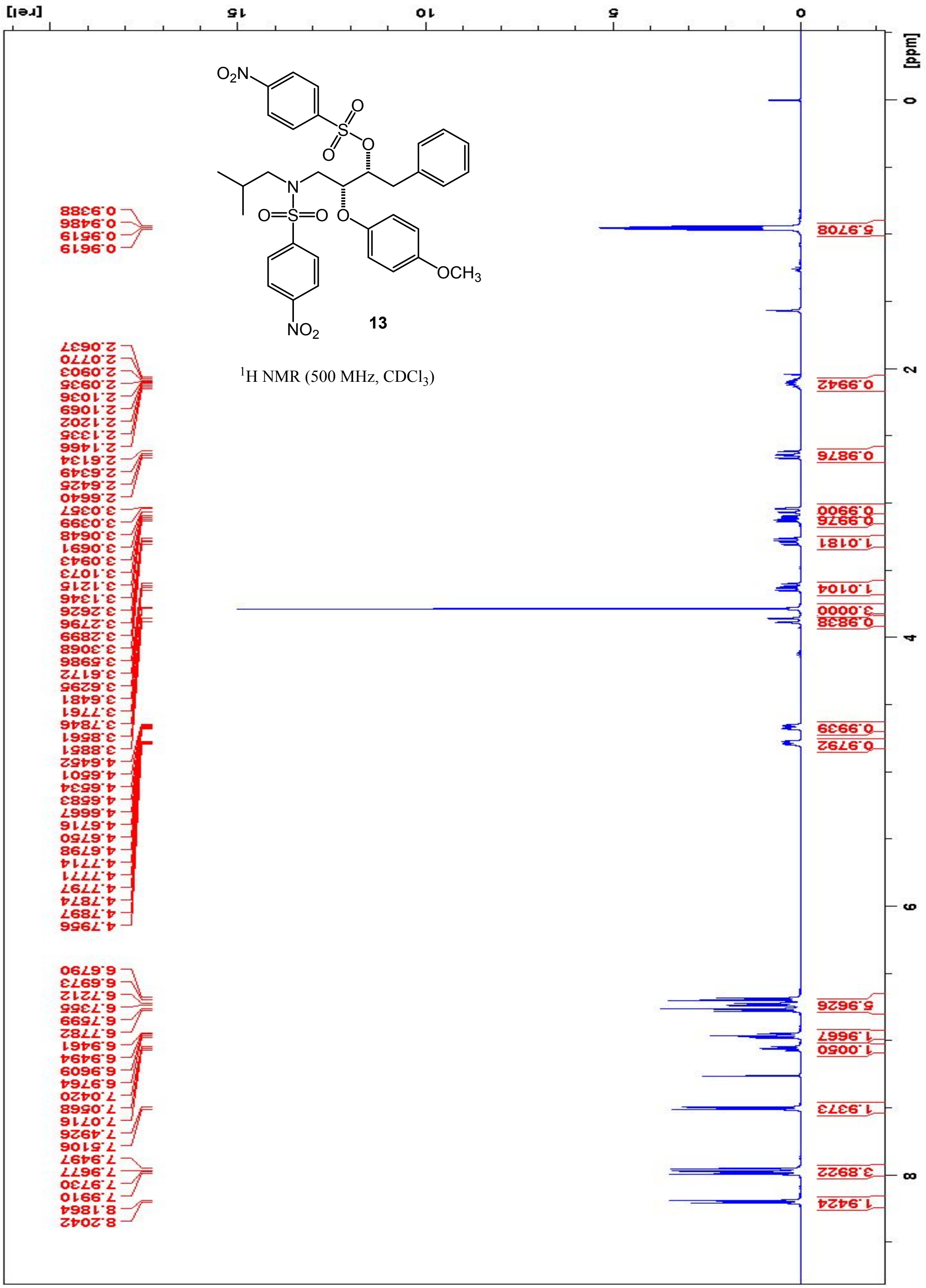

13

^{1}H NMR (500 MHz, $CDCl_3$)

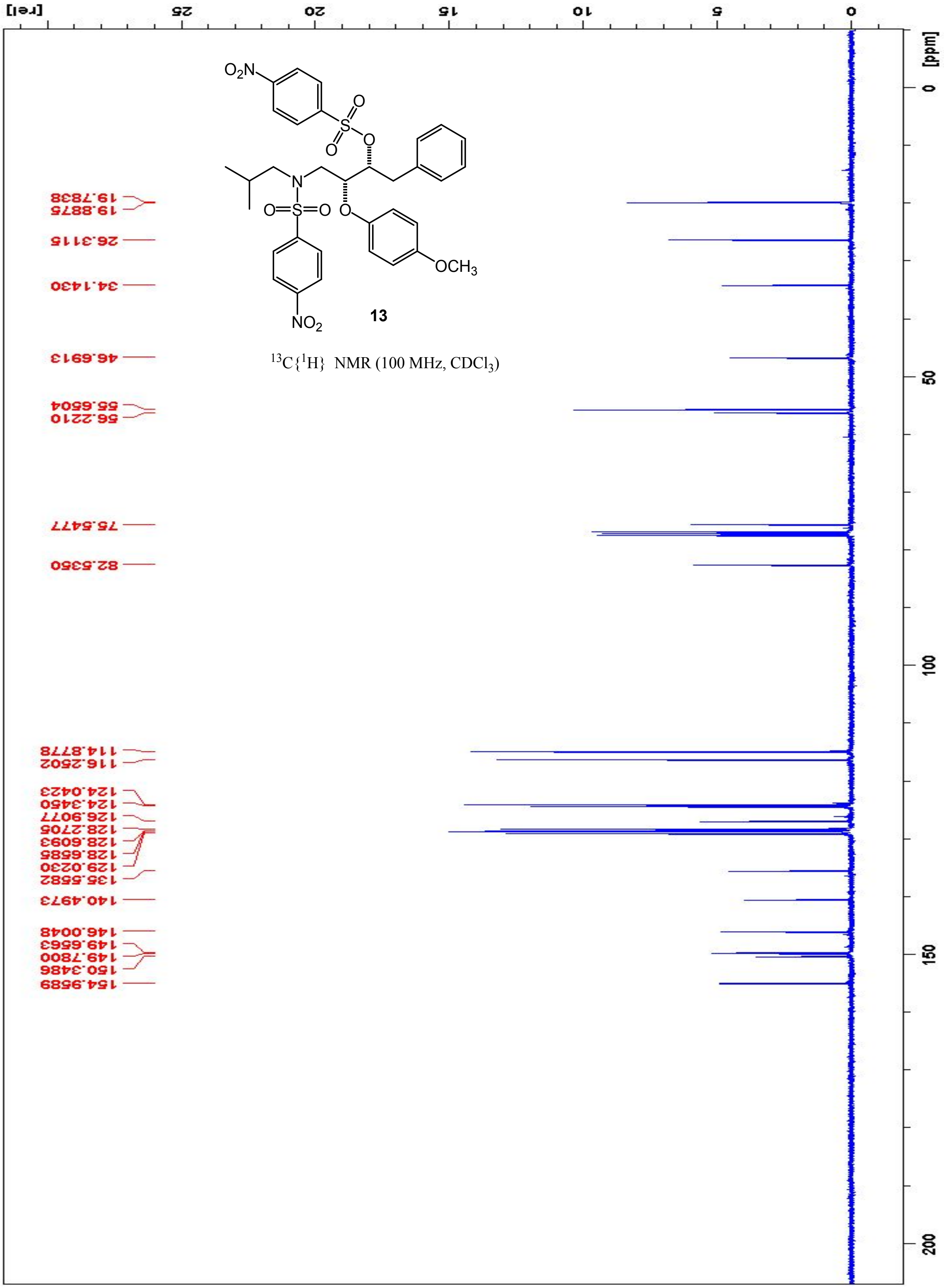

O_2N
NO_2
OCH_3
13
$^{13}C\{^1H\}$ NMR (100 MHz, $CDCl_3$)
19.7838
19.8875
26.3115
34.1430
46.6913
55.6504
56.2210
75.5477
82.5350
114.8778
116.2502
124.0423
124.3450
126.9077
128.2705
128.6093
128.6585
129.0230
135.5582
140.4973
146.0048
149.6563
149.7800
150.3486
154.9589
[ppm]
[rel]

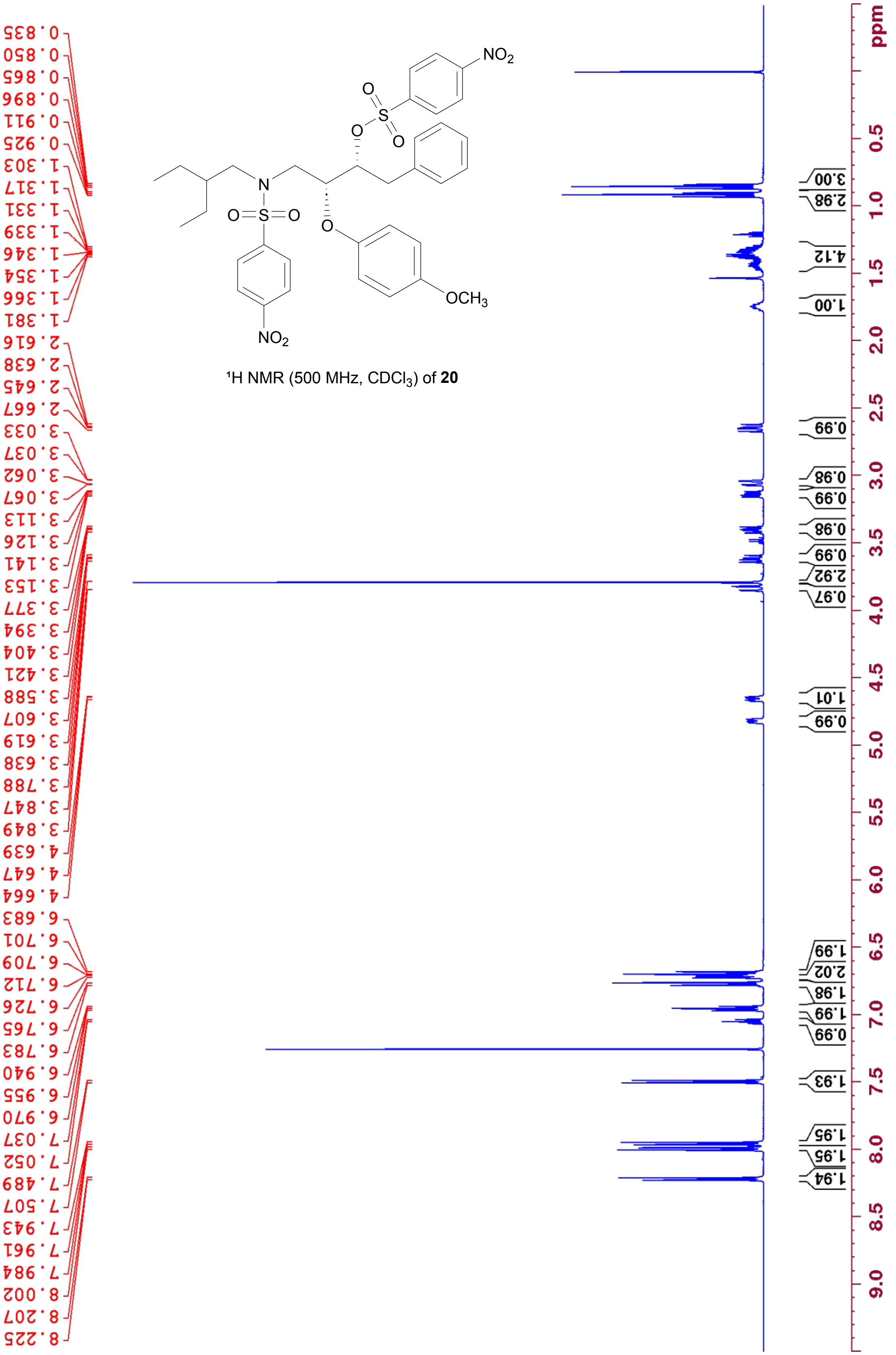

^1H NMR (500 MHz, $CDCl_3$) of **20**

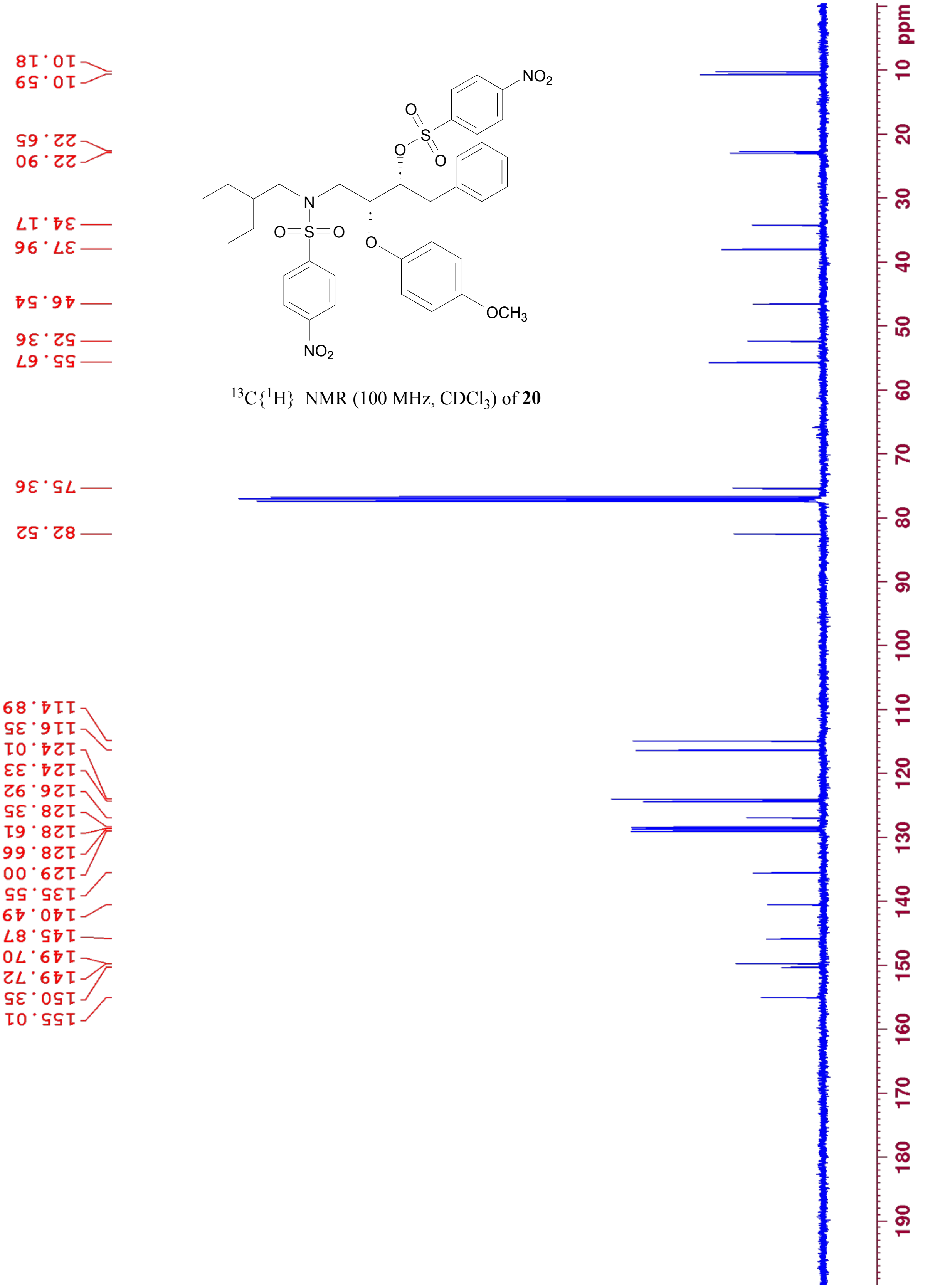

$^{13}C\{^{1}H\}$ NMR (100 MHz, $CDCl_3$) of **20**

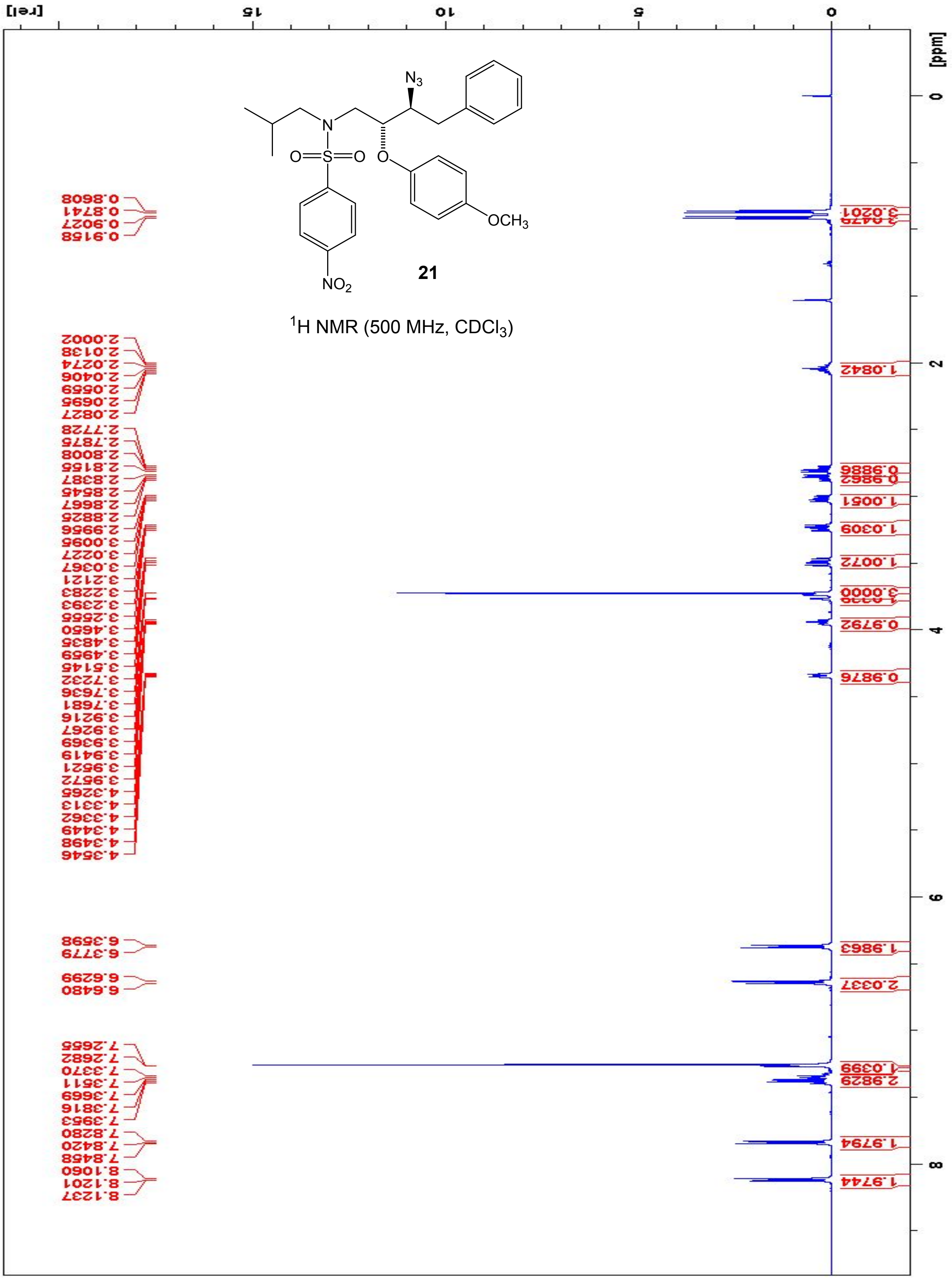

21

^{1}H NMR (500 MHz, $CDCl_3$)

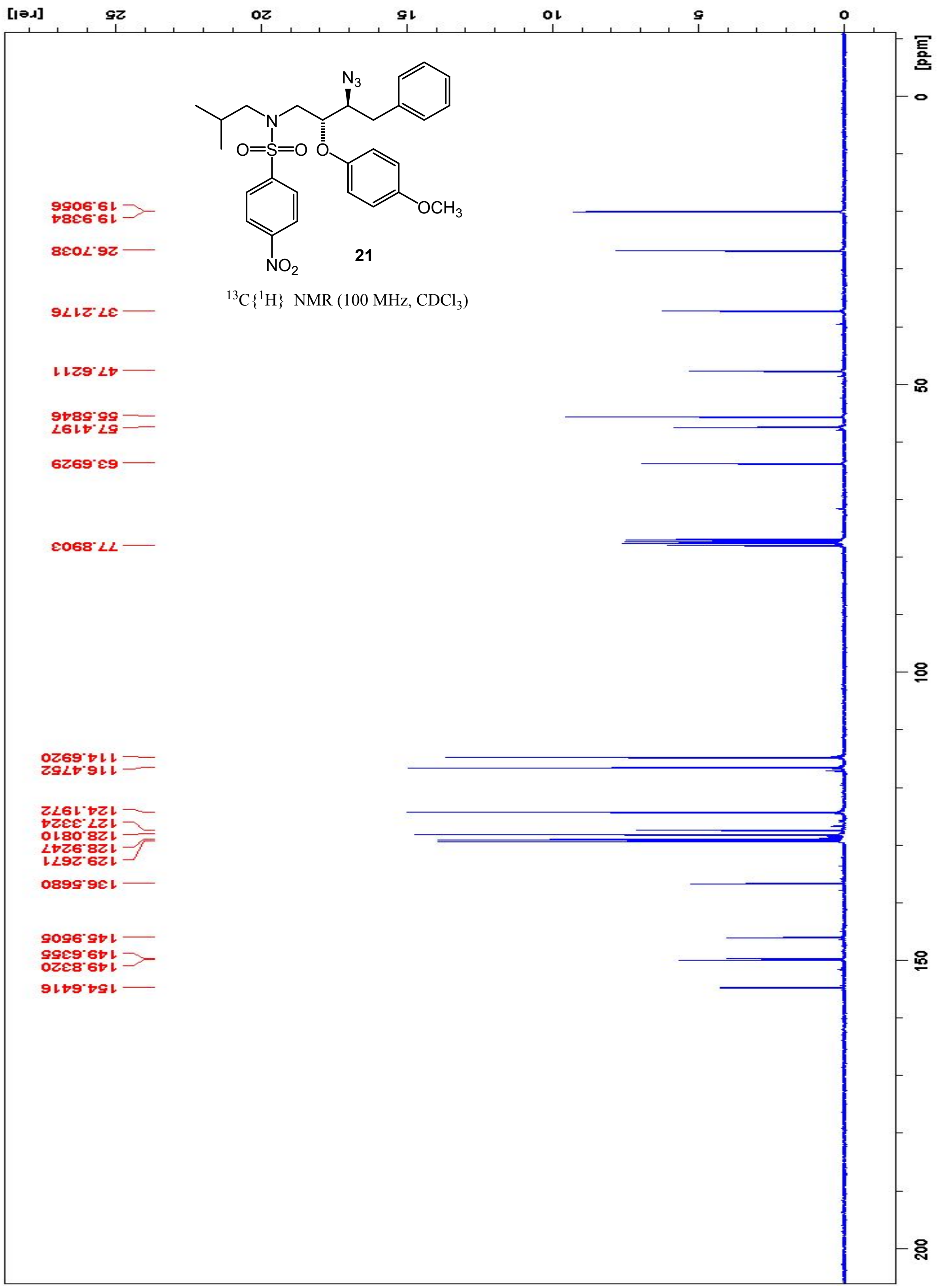

21

$^{13}C\{^{1}H\}$ NMR (100 MHz, $CDCl_3$)

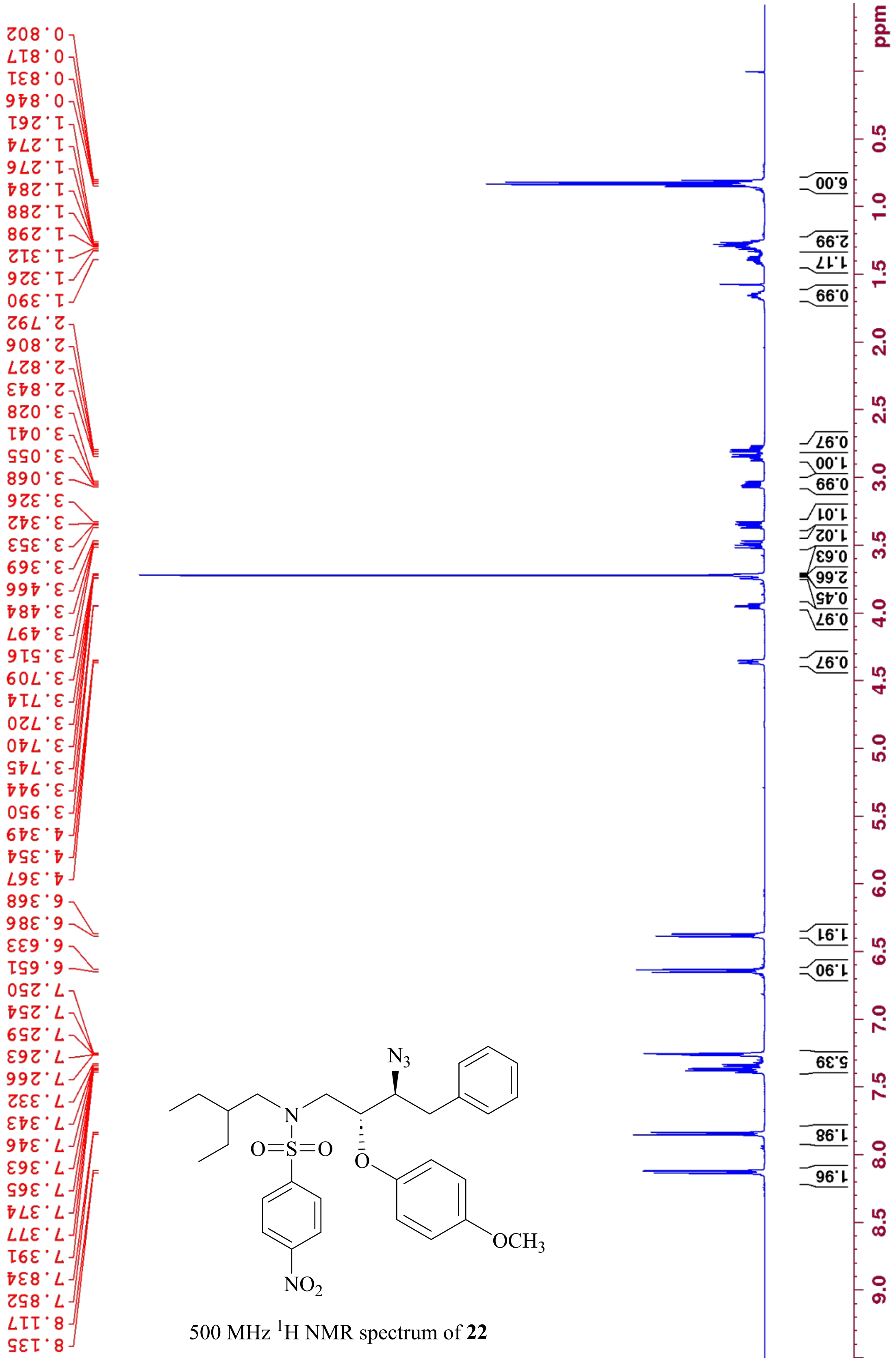

500 MHz ^{1}H NMR spectrum of **22**

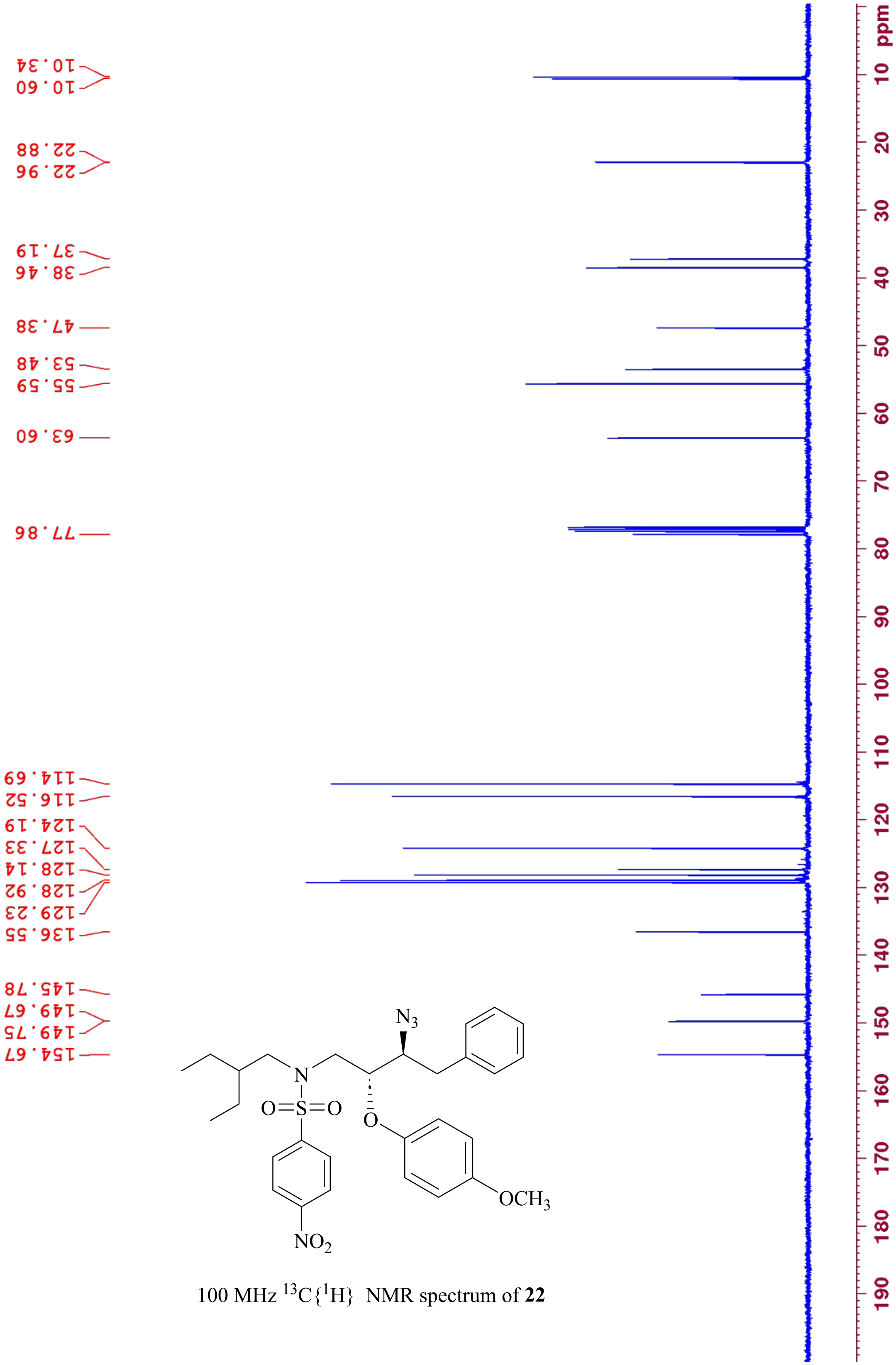

100 MHz $^{13}C\{^1H\}$ NMR spectrum of **22**

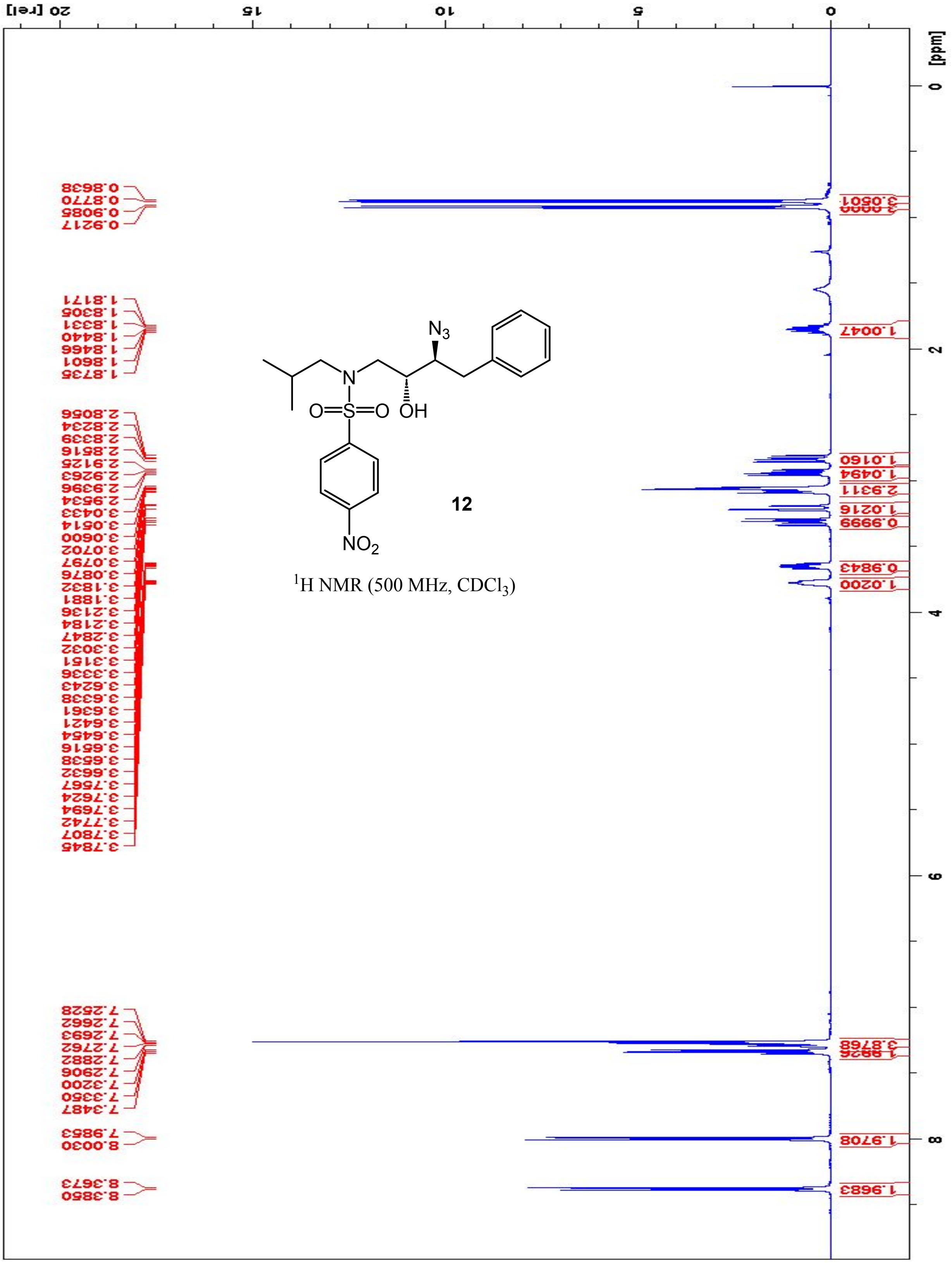

12

^{1}H NMR (500 MHz, $CDCl_3$)

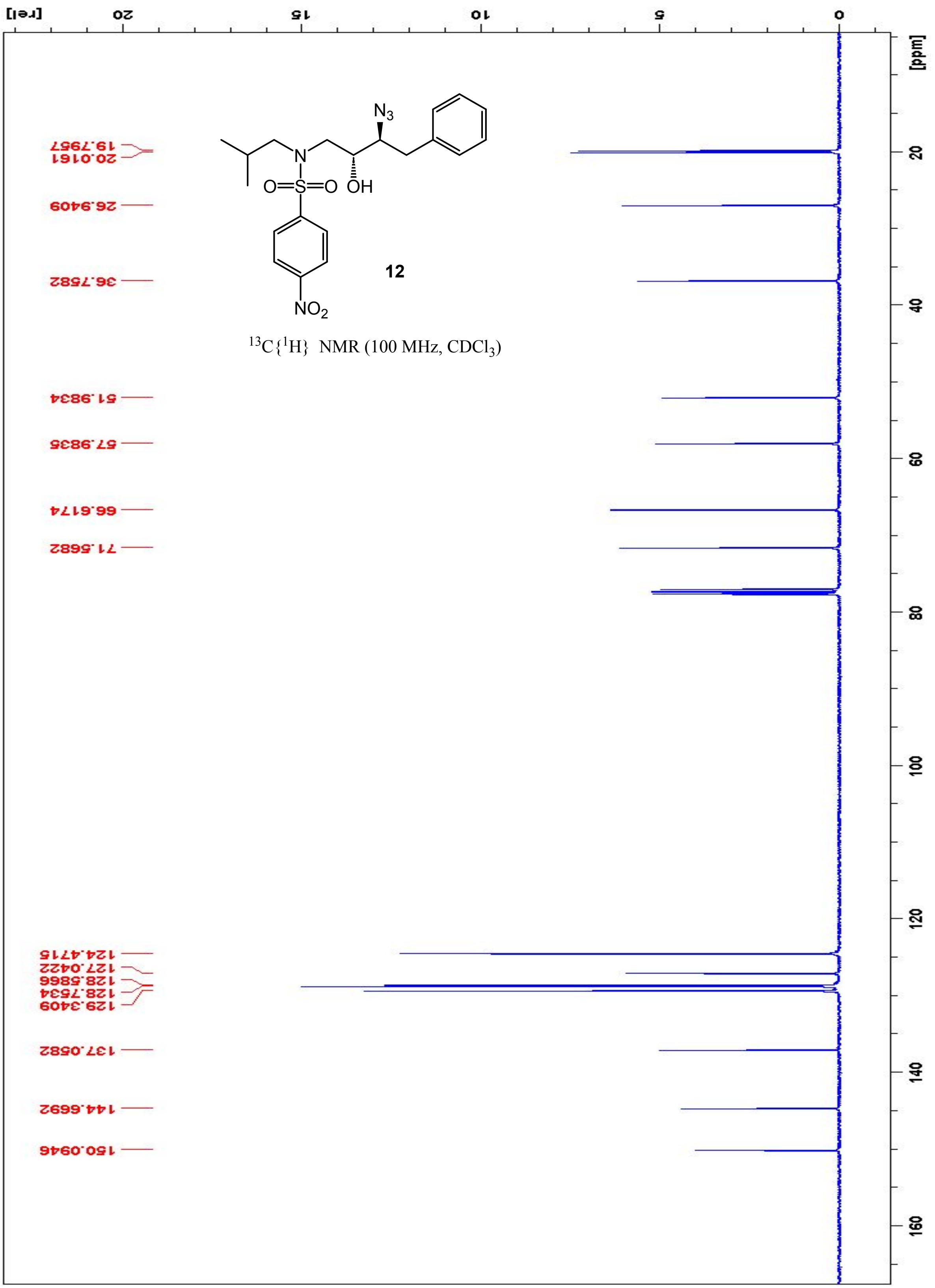

12

$^{13}C\{^{1}H\}$ NMR (100 MHz, $CDCl_3$)

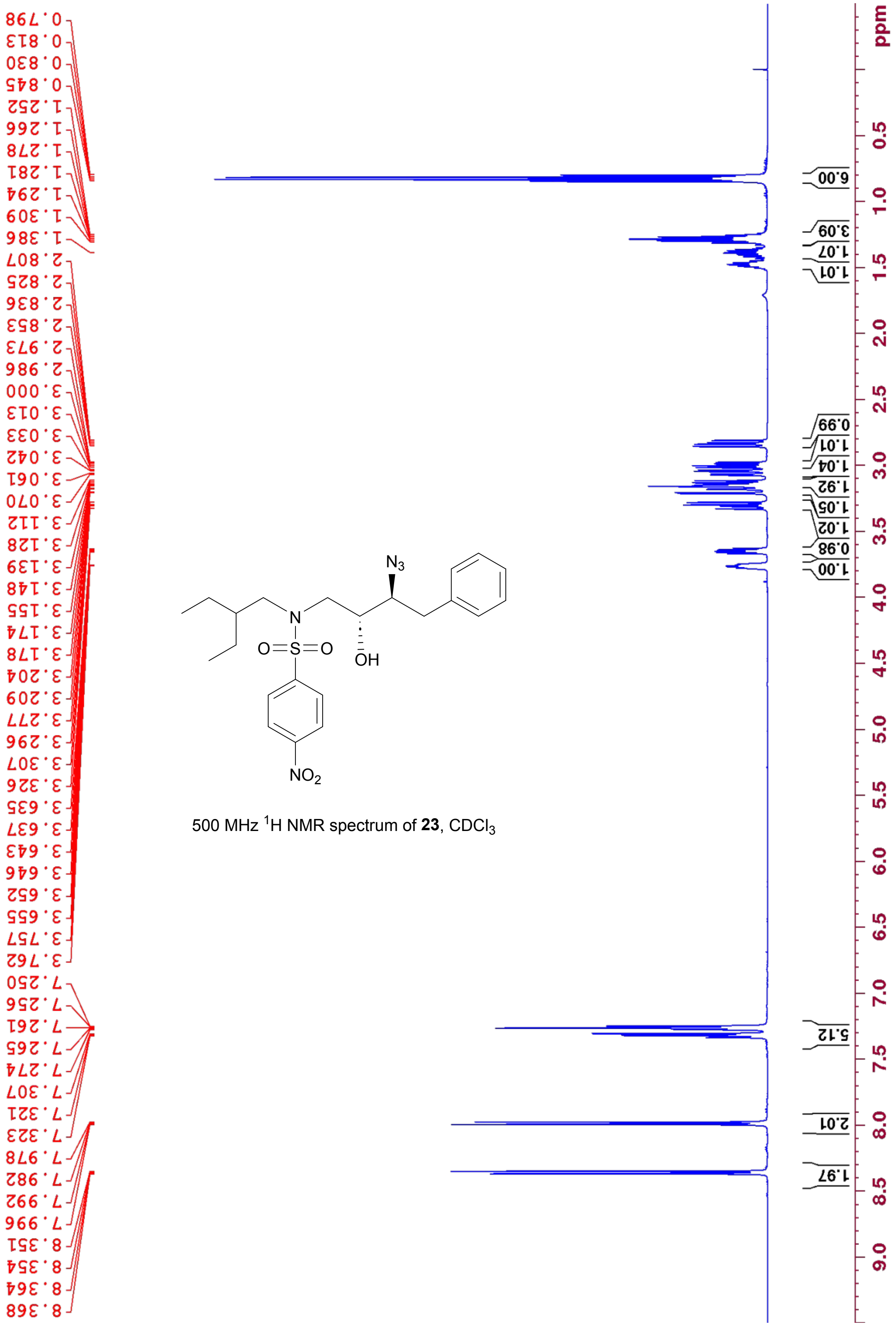

500 MHz ^{1}H NMR spectrum of **23**, $CDCl_3$

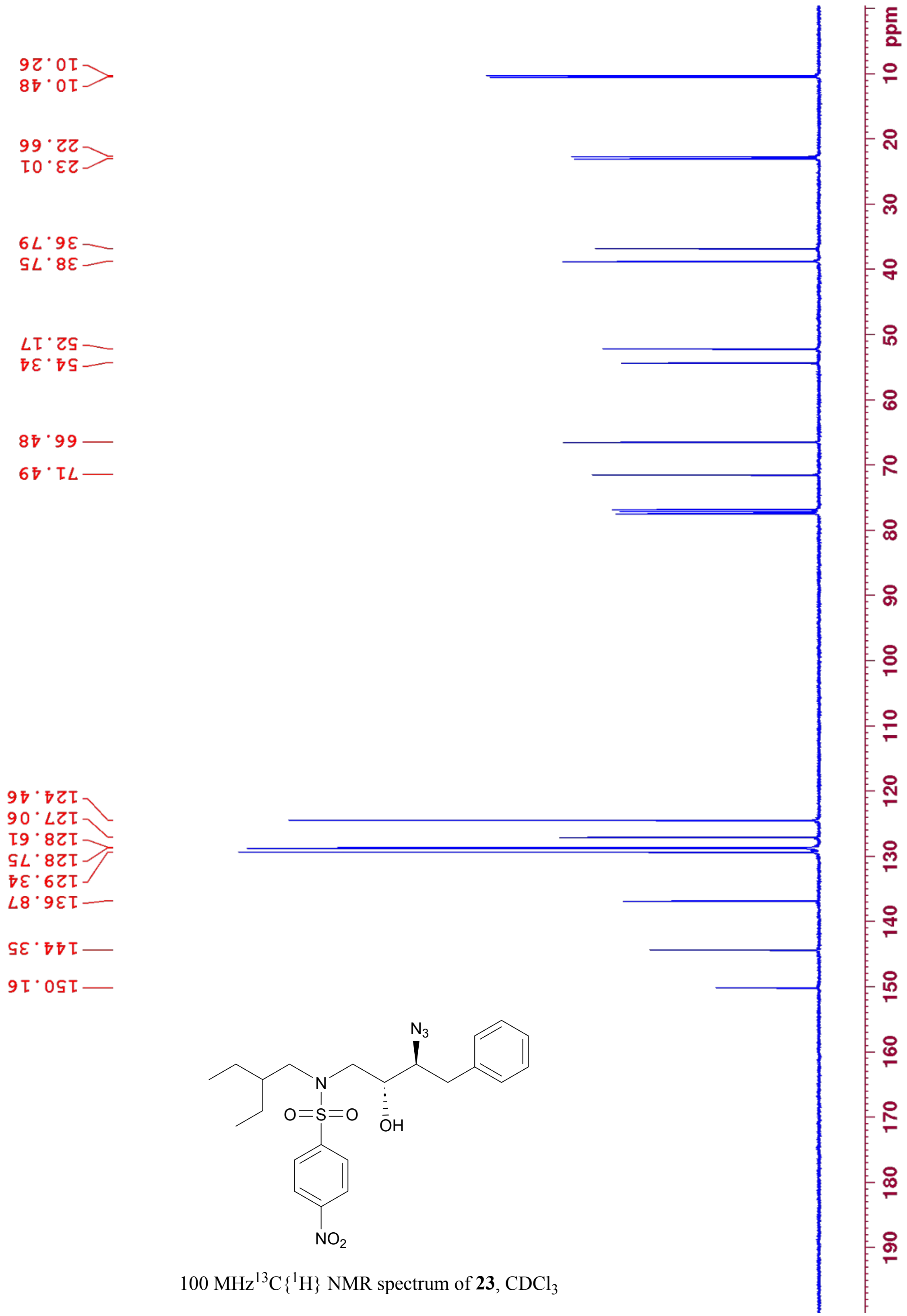

100 MHz $^{13}C\{^{1}H\}$ NMR spectrum of **23**, $CDCl_3$

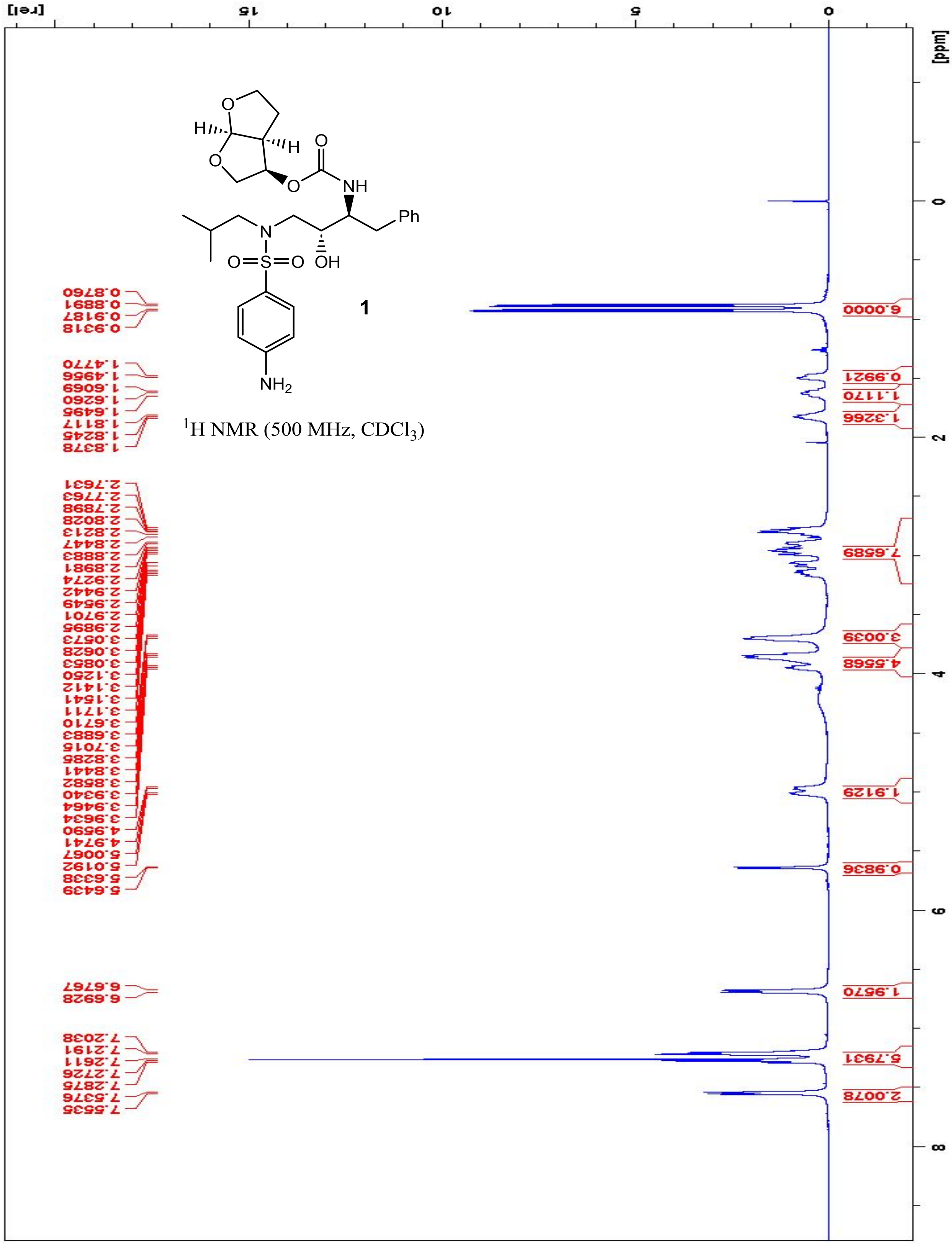

1

^{1}H NMR (500 MHz, $CDCl_3$)

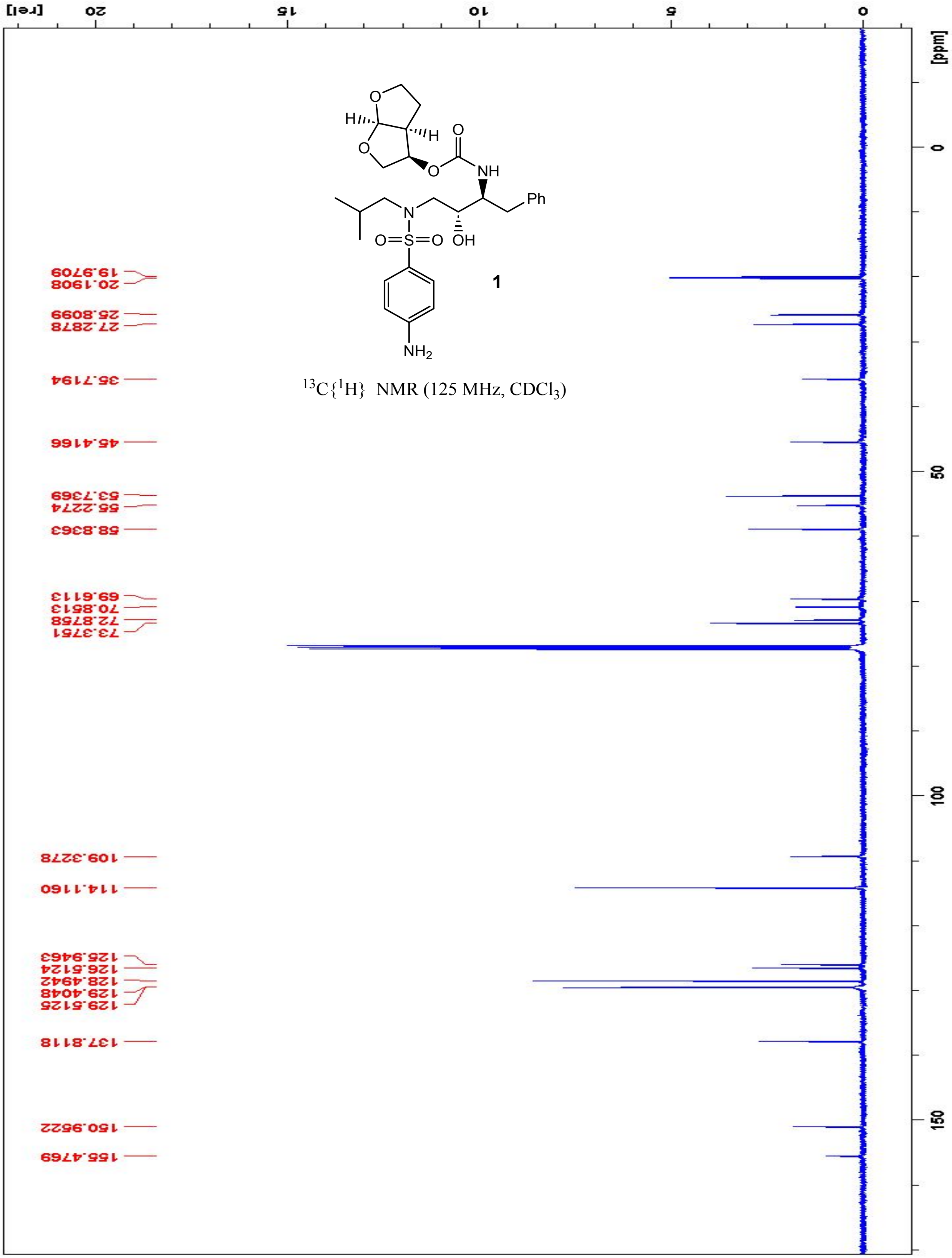

$^{13}C\{^{1}H\}$ NMR (125 MHz, $CDCl_3$)

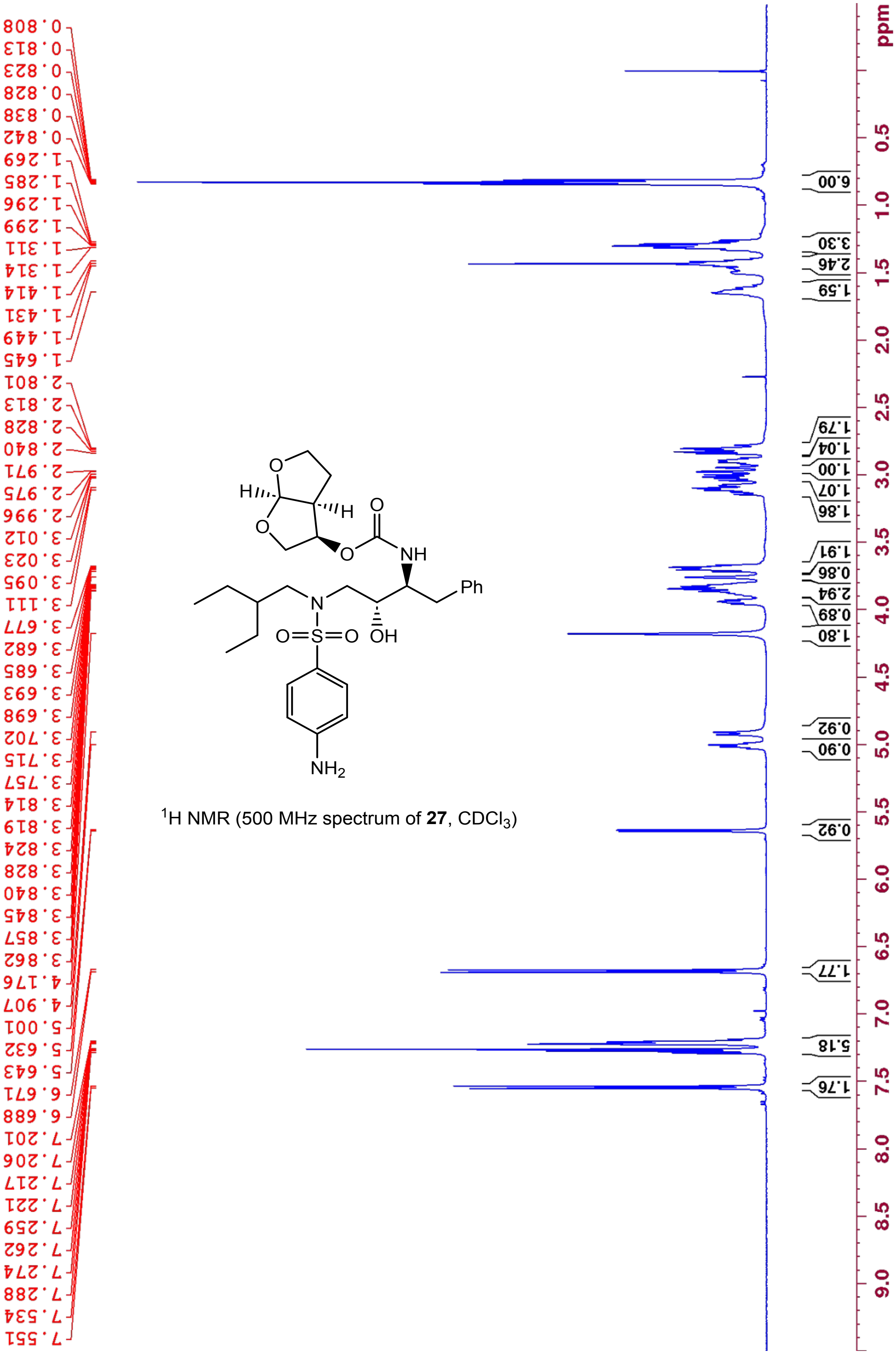

^{1}H NMR (500 MHz spectrum of **27**, $CDCl_3$)

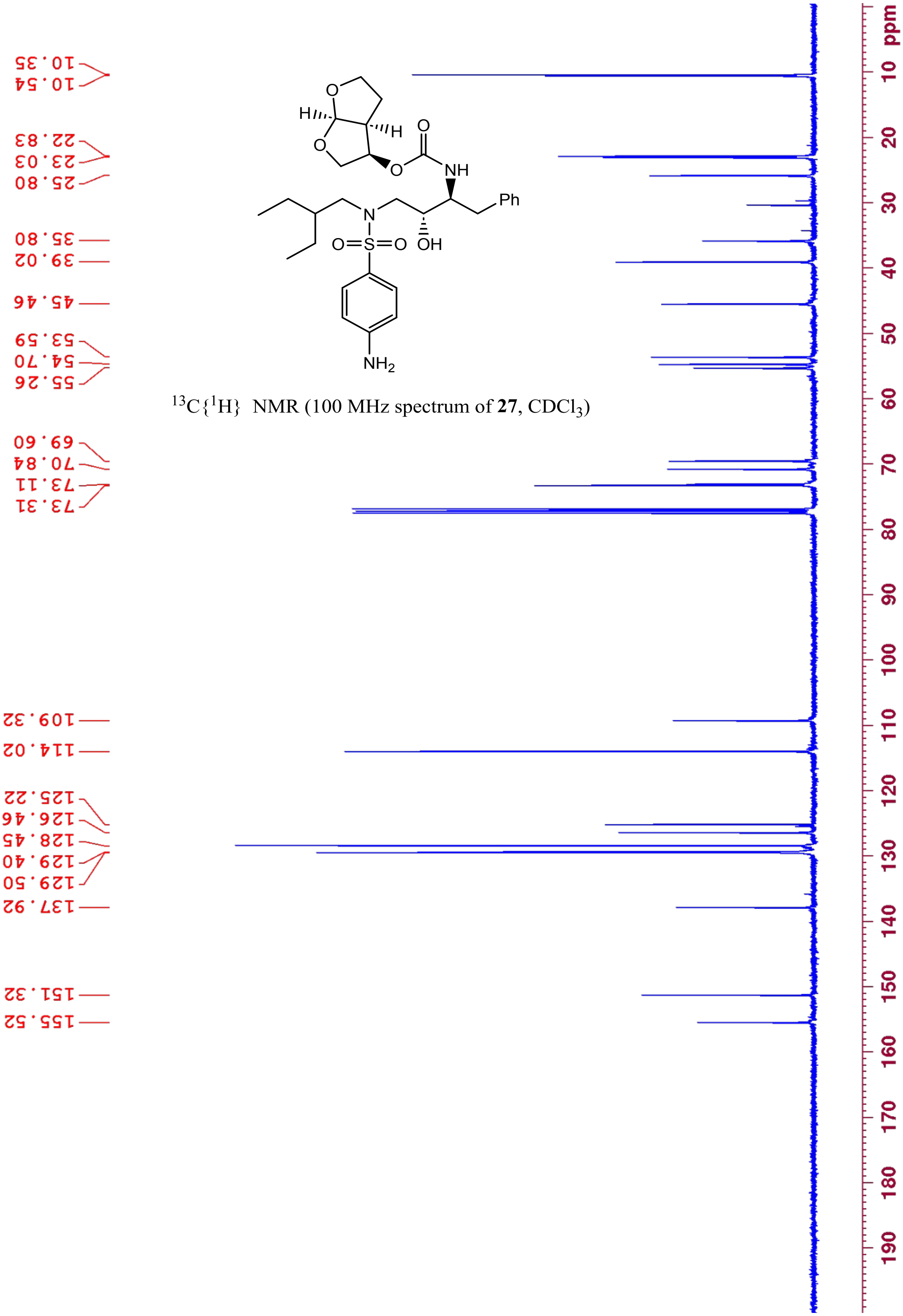

$^{13}C\{^{1}H\}$ NMR (100 MHz spectrum of **27**, $CDCl_3$)

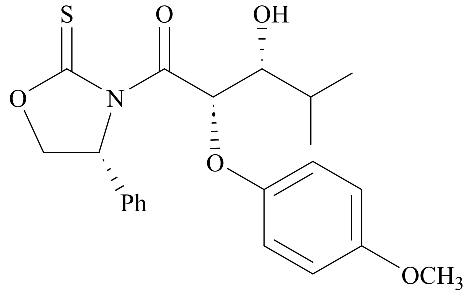

^{1}H NMR (500 MHz spectrum of **28**, $CDCl_3$)

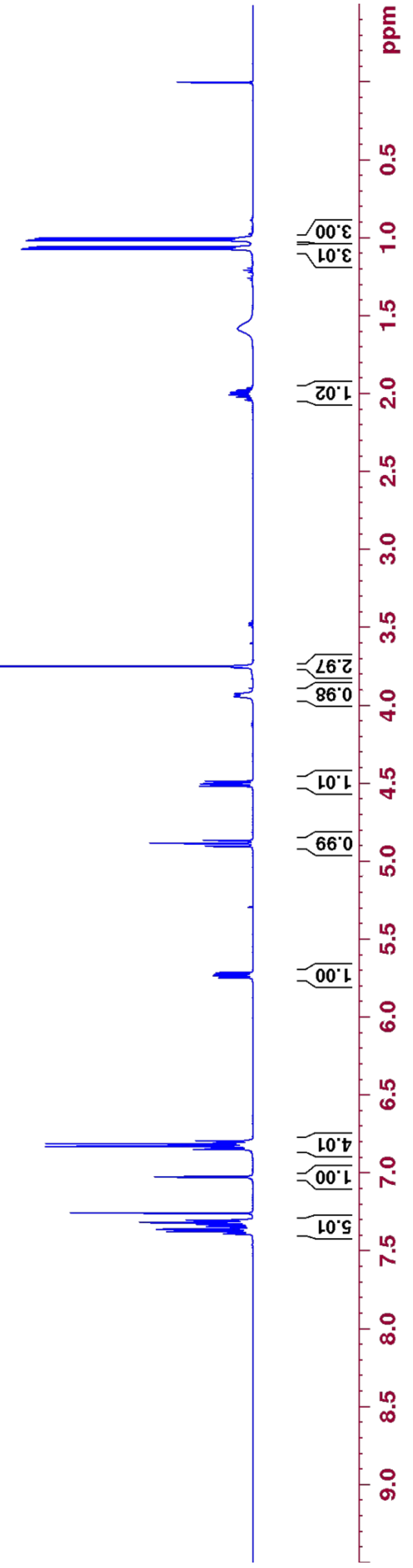

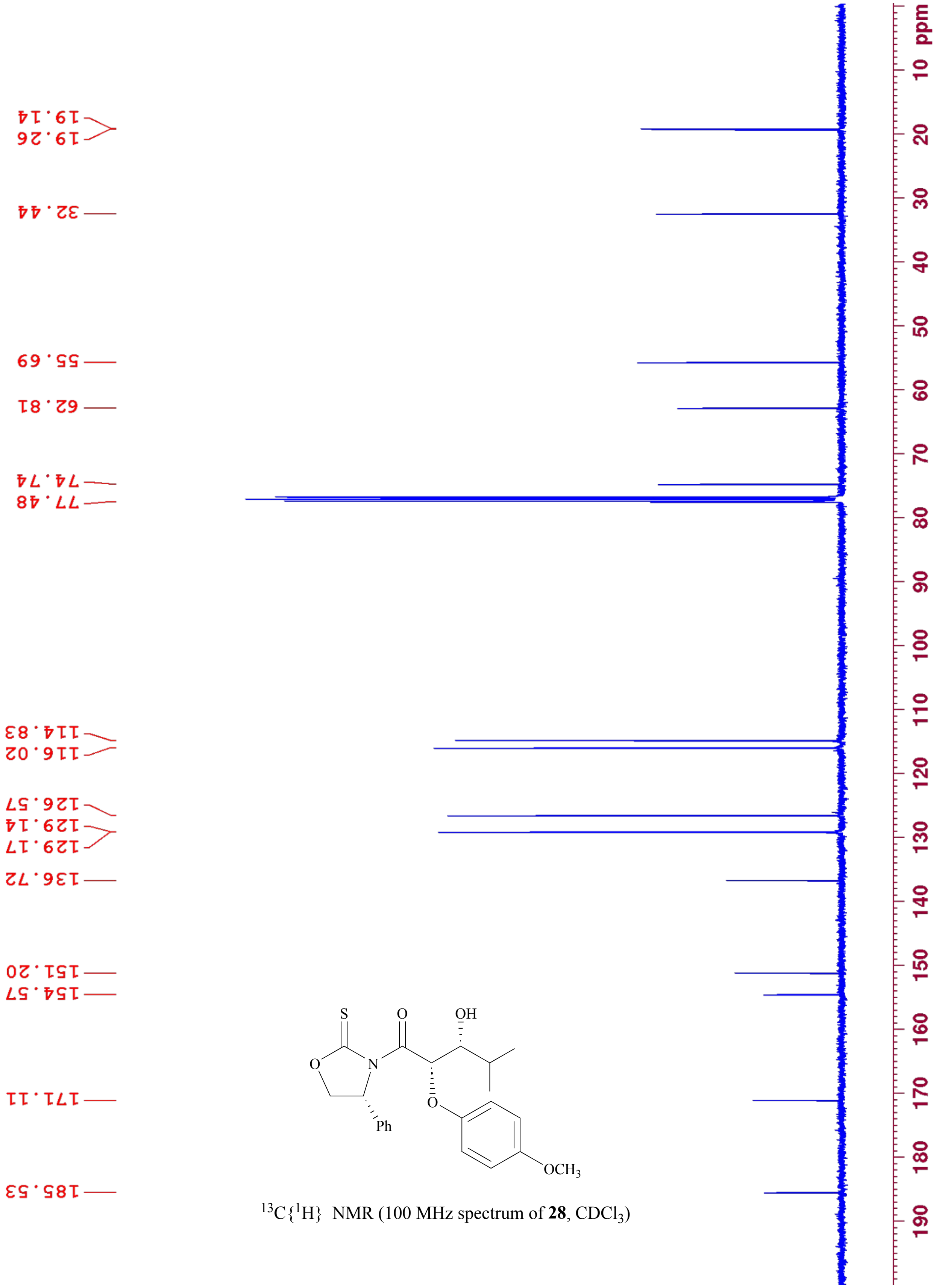

$^{13}C\{^{1}H\}$ NMR (100 MHz spectrum of **28**, $CDCl_3$)

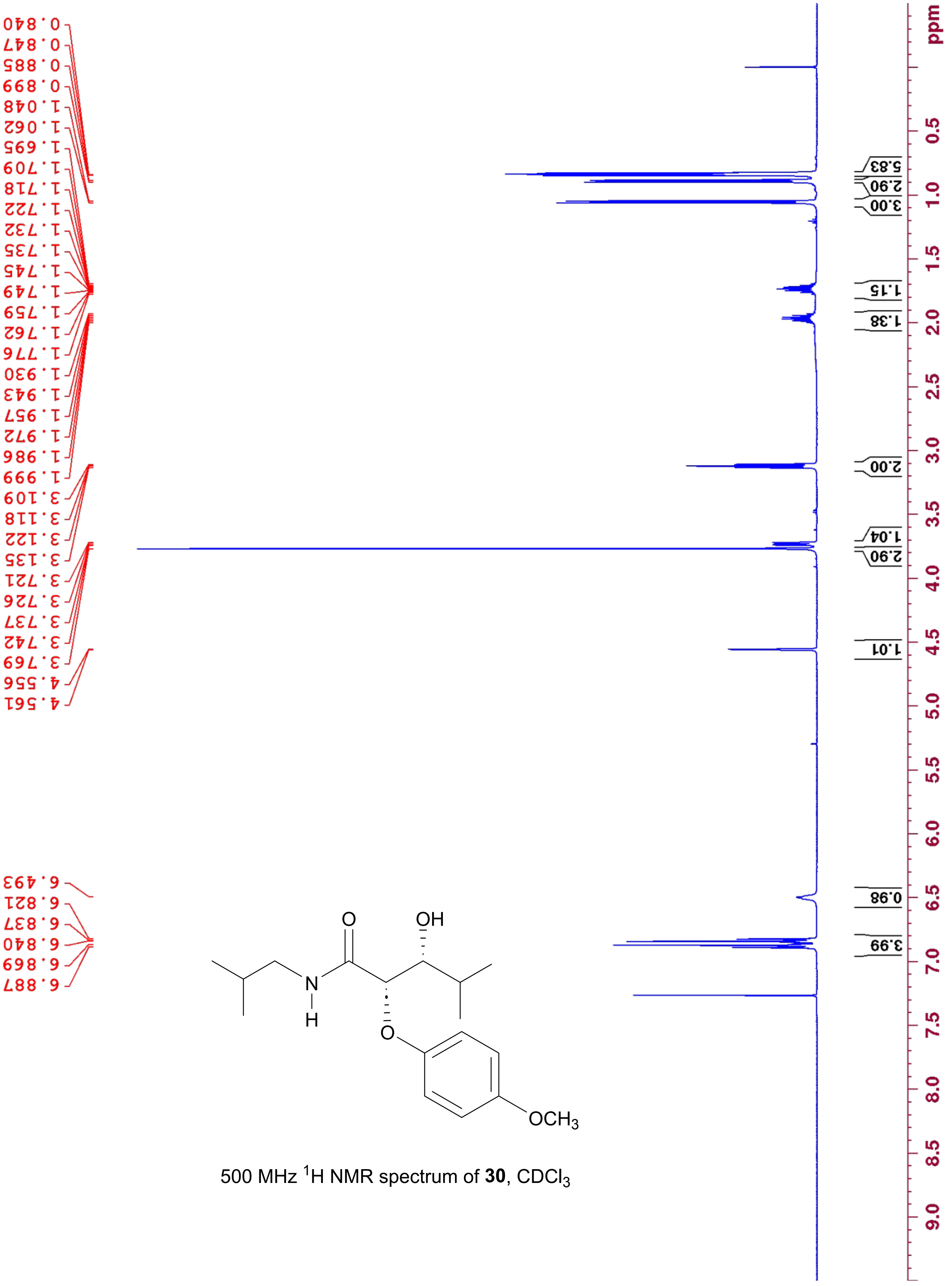

500 MHz ^{1}H NMR spectrum of **30**, $CDCl_3$

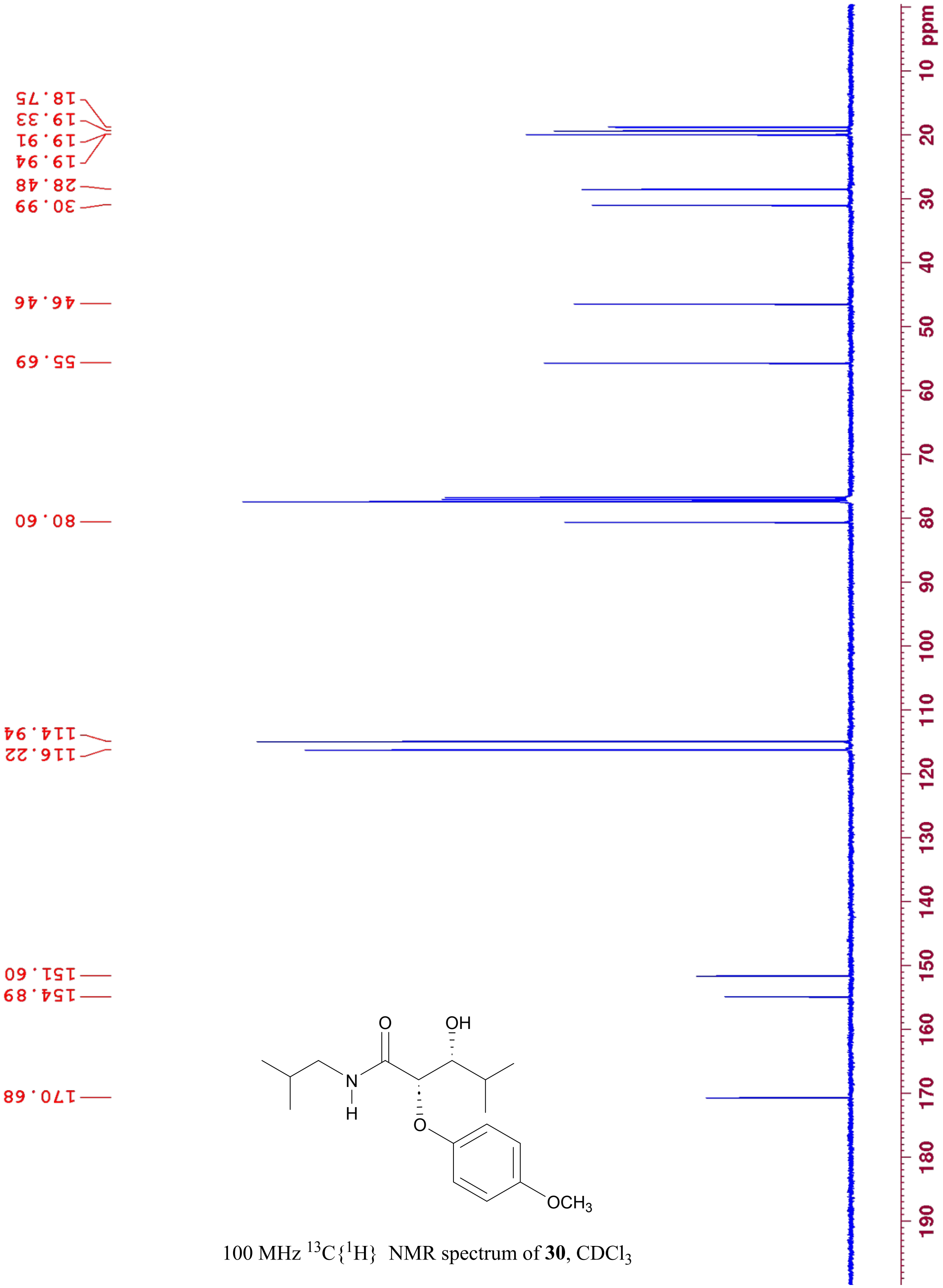

100 MHz $^{13}C\{^{1}H\}$ NMR spectrum of **30**, $CDCl_3$

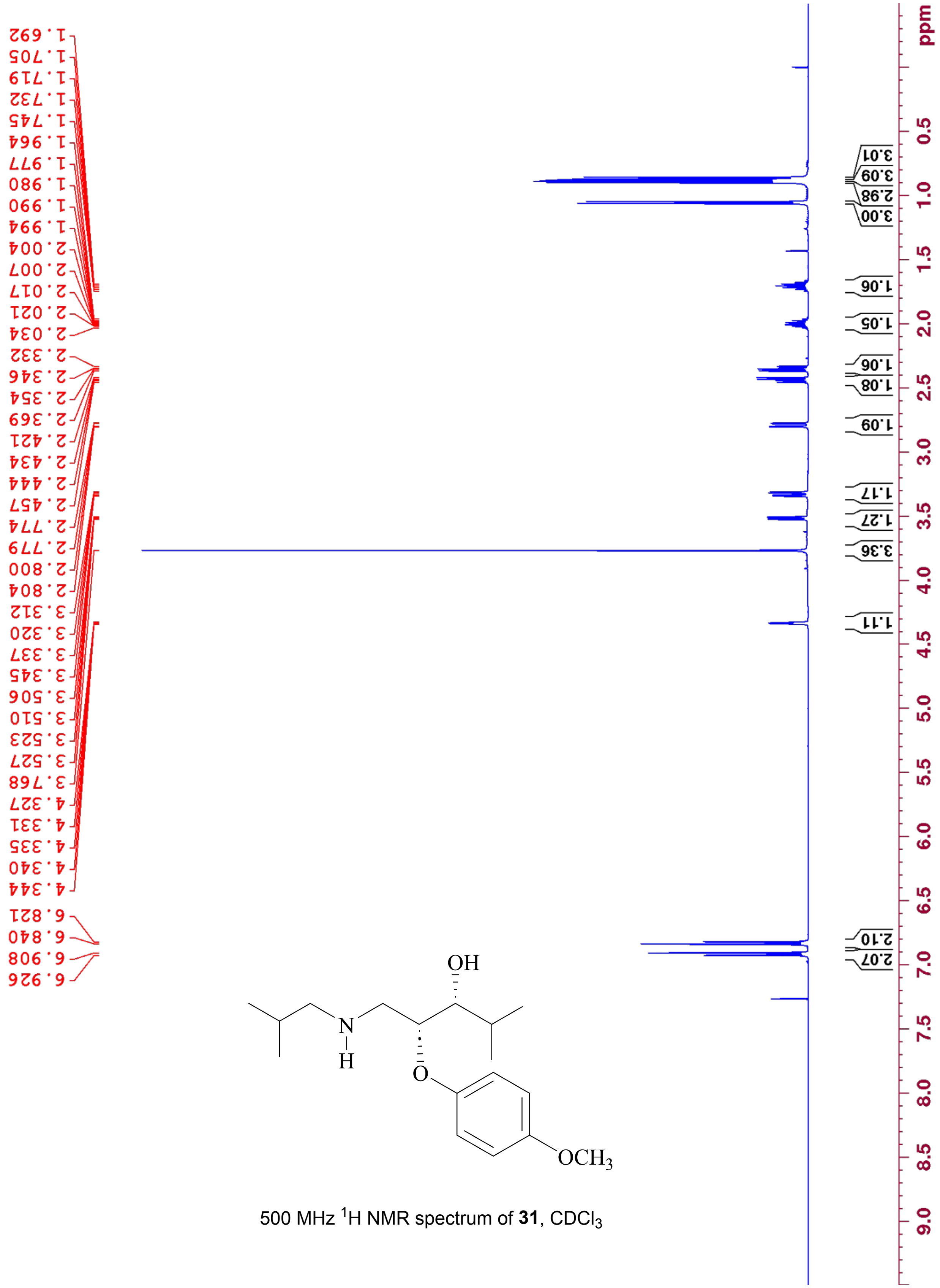

500 MHz ^{1}H NMR spectrum of **31**, $CDCl_3$

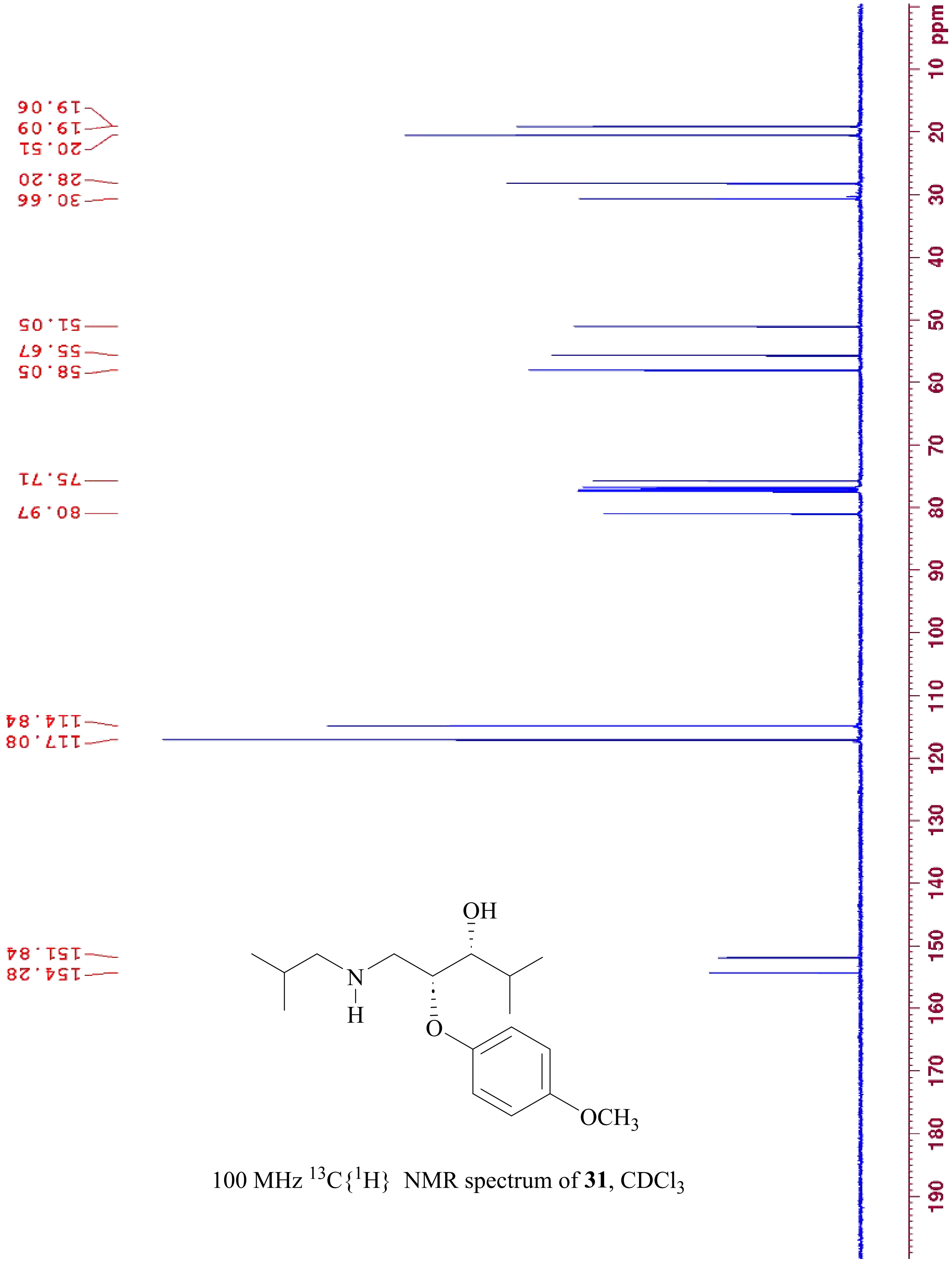

100 MHz $^{13}C\{^{1}H\}$ NMR spectrum of **31**, $CDCl_3$

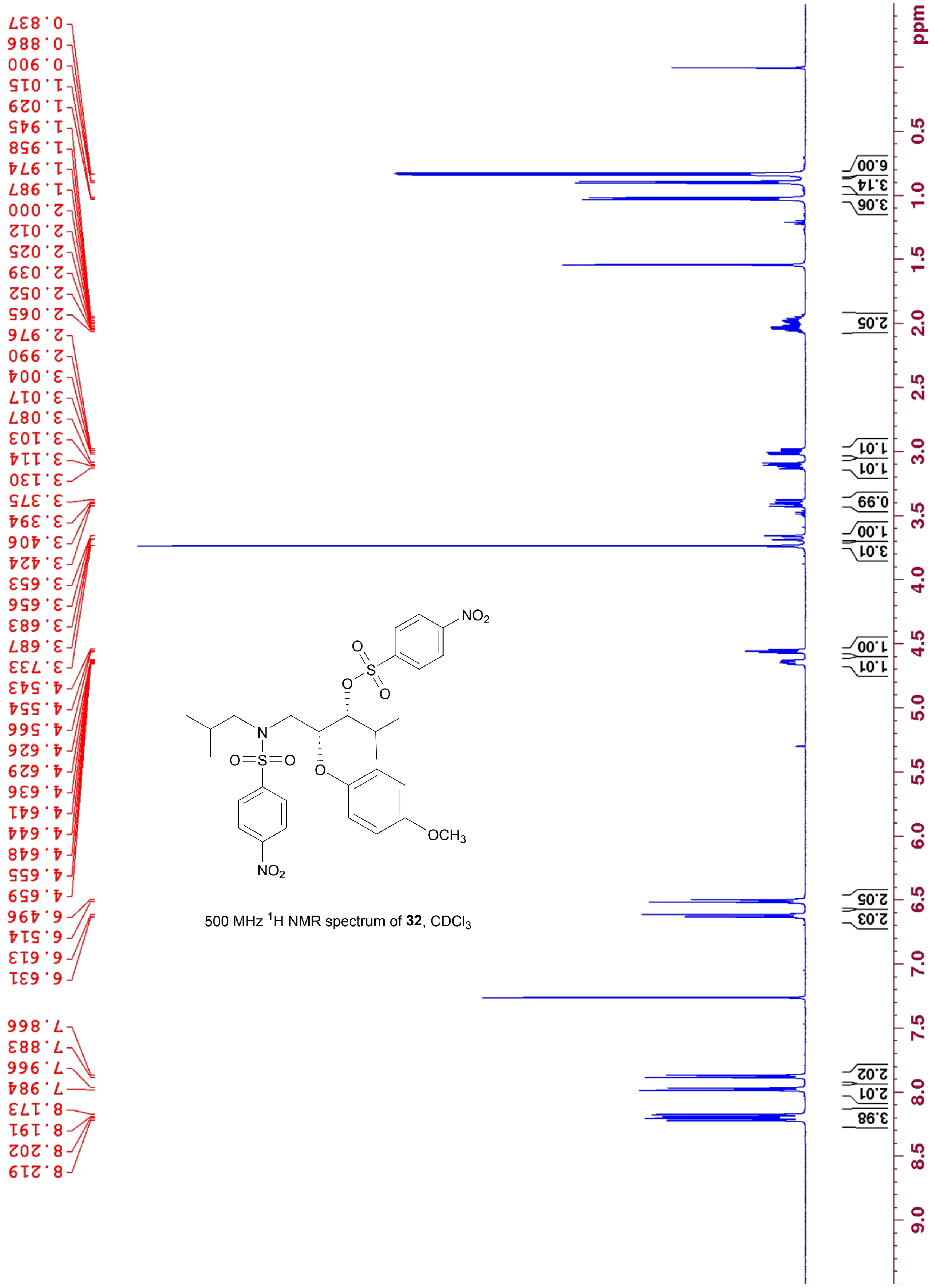

500 MHz ^{1}H NMR spectrum of **32**, $CDCl_3$

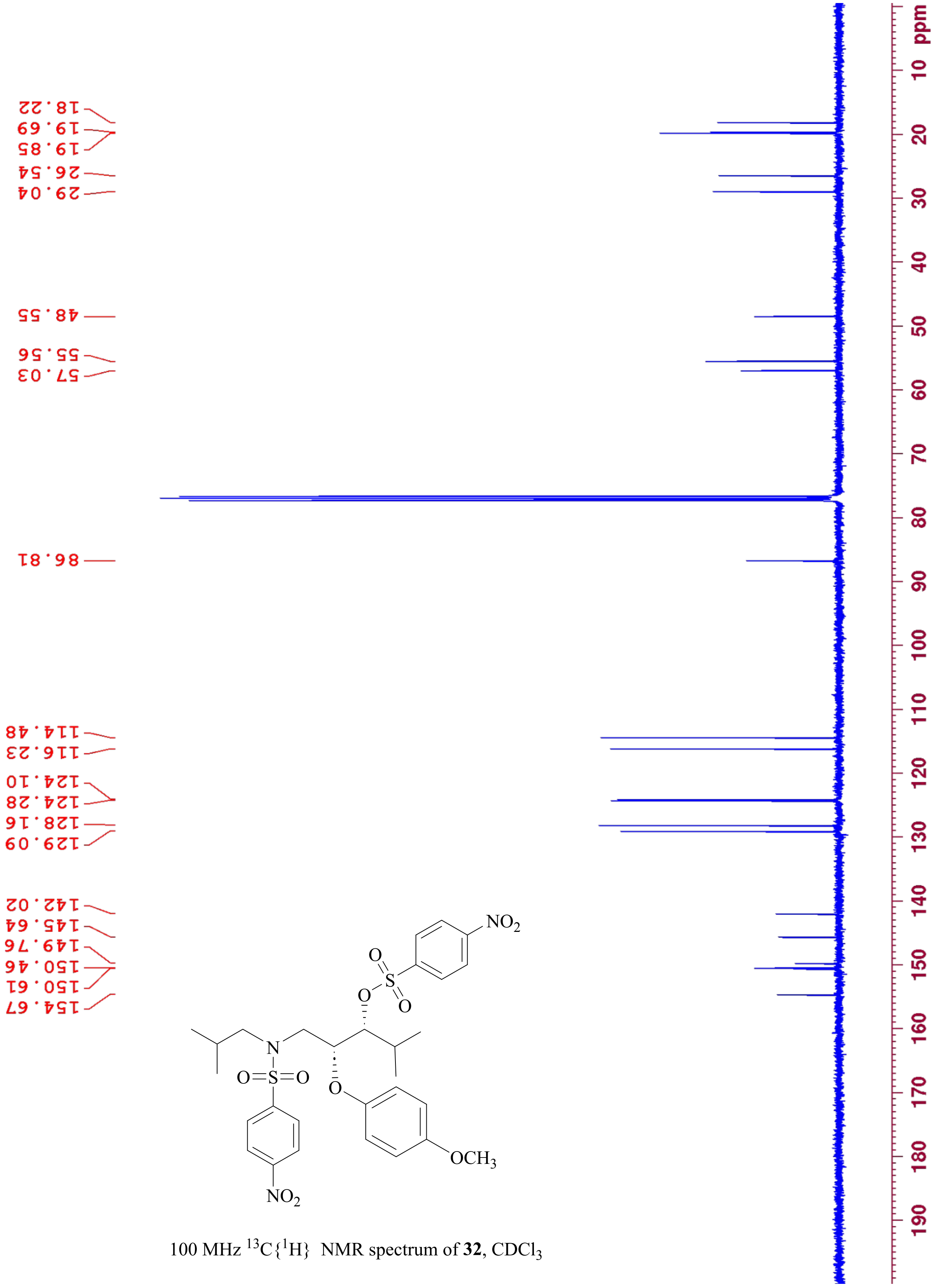

100 MHz $^{13}C\{^{1}H\}$ NMR spectrum of **32**, $CDCl_3$

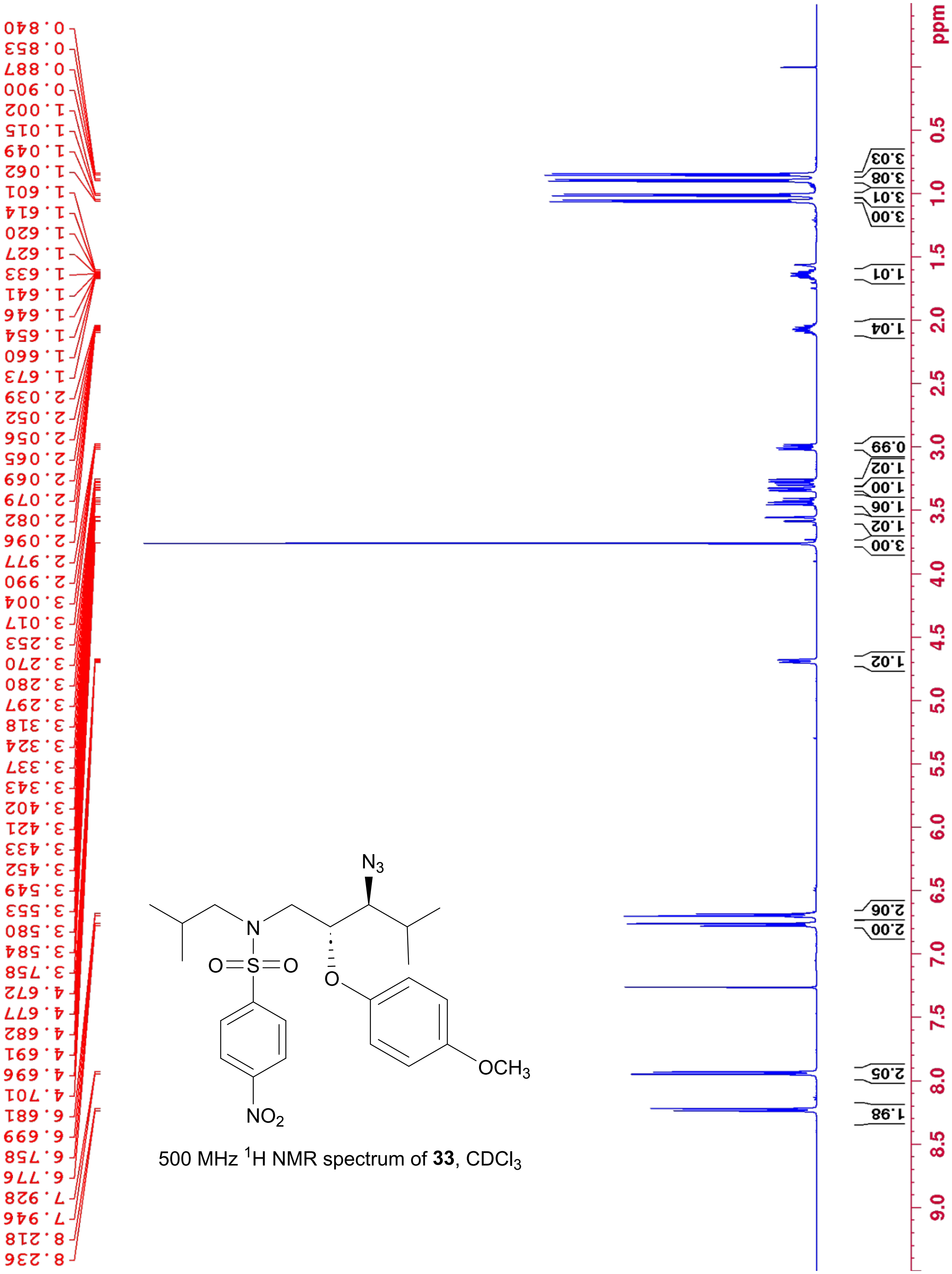

500 MHz ¹H NMR spectrum of **33**, $CDCl_3$

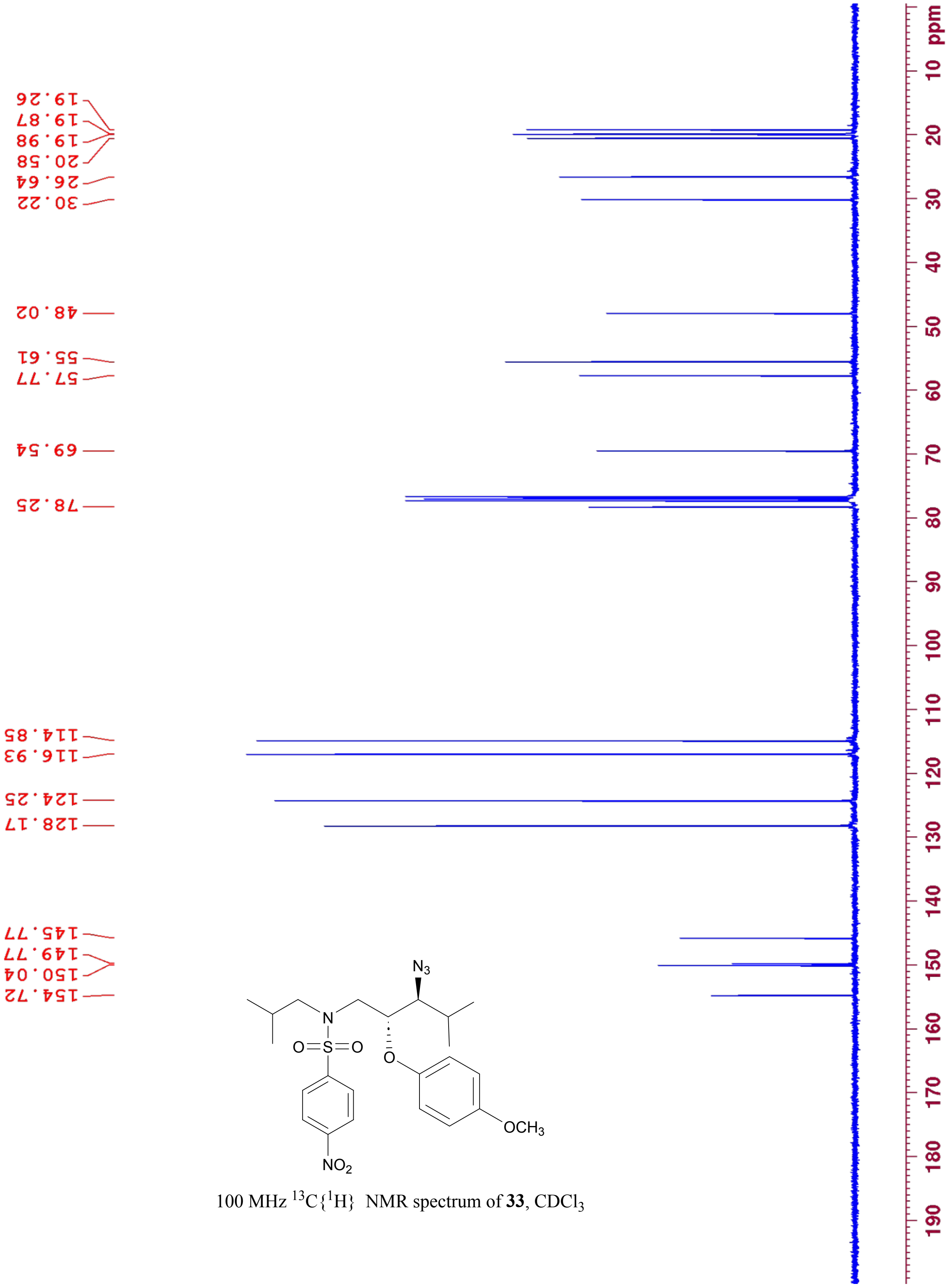

100 MHz $^{13}C\{^1H\}$ NMR spectrum of **33**, $CDCl_3$

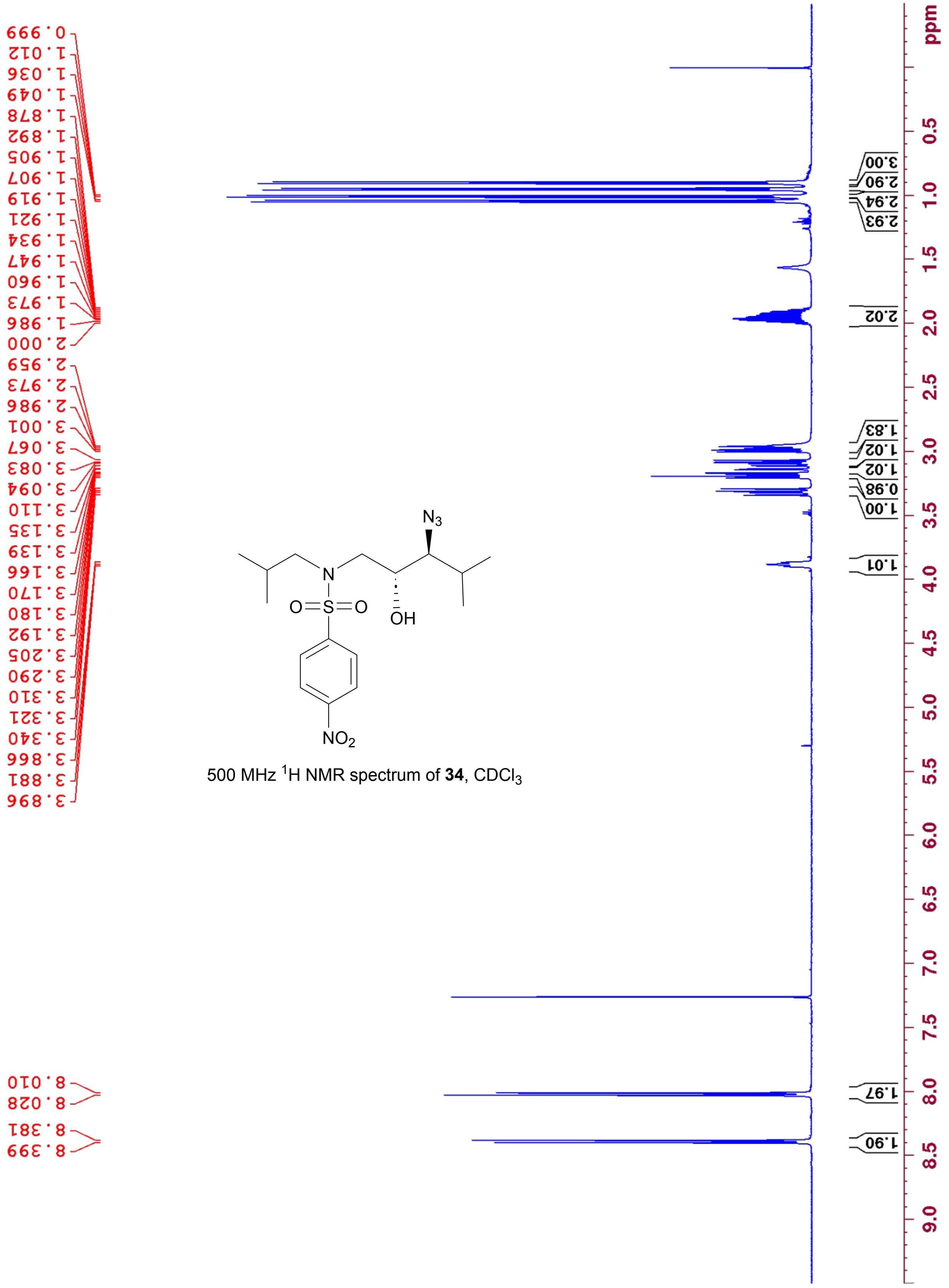

500 MHz ^{1}H NMR spectrum of **34**, $CDCl_3$

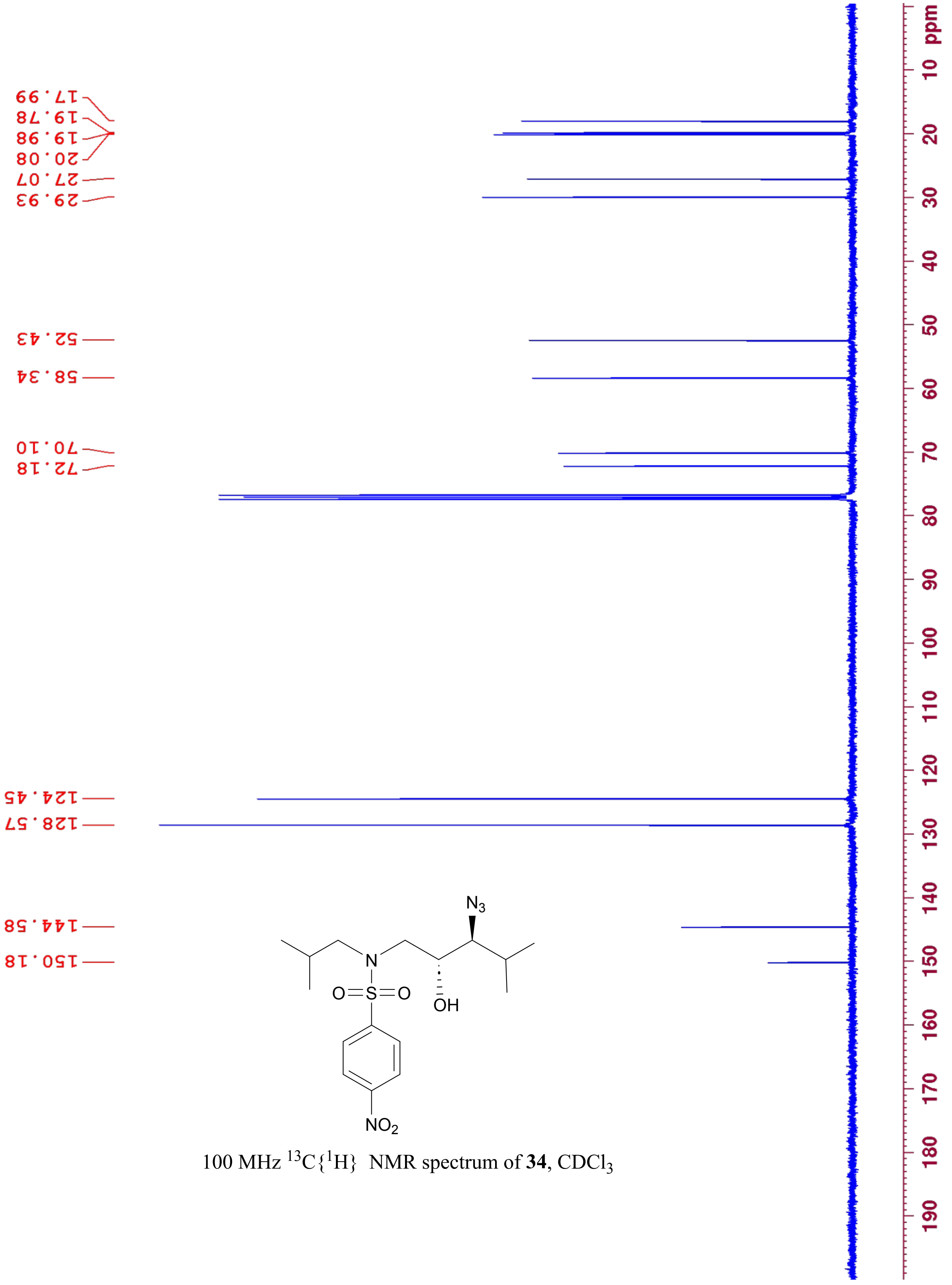

100 MHz $^{13}C\{^{1}H\}$ NMR spectrum of **34**, $CDCl_3$

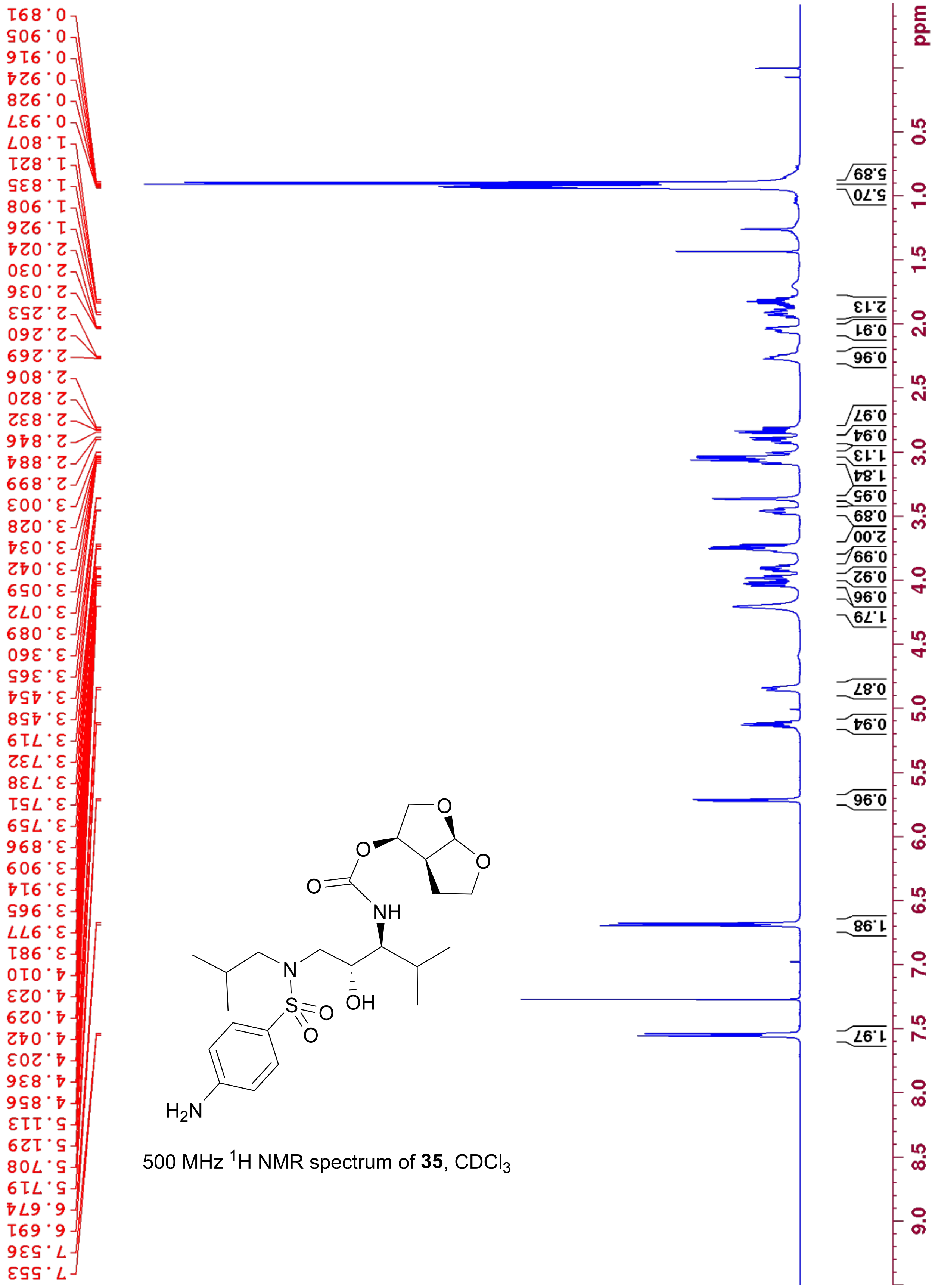

500 MHz ^{1}H NMR spectrum of **35**, $CDCl_3$

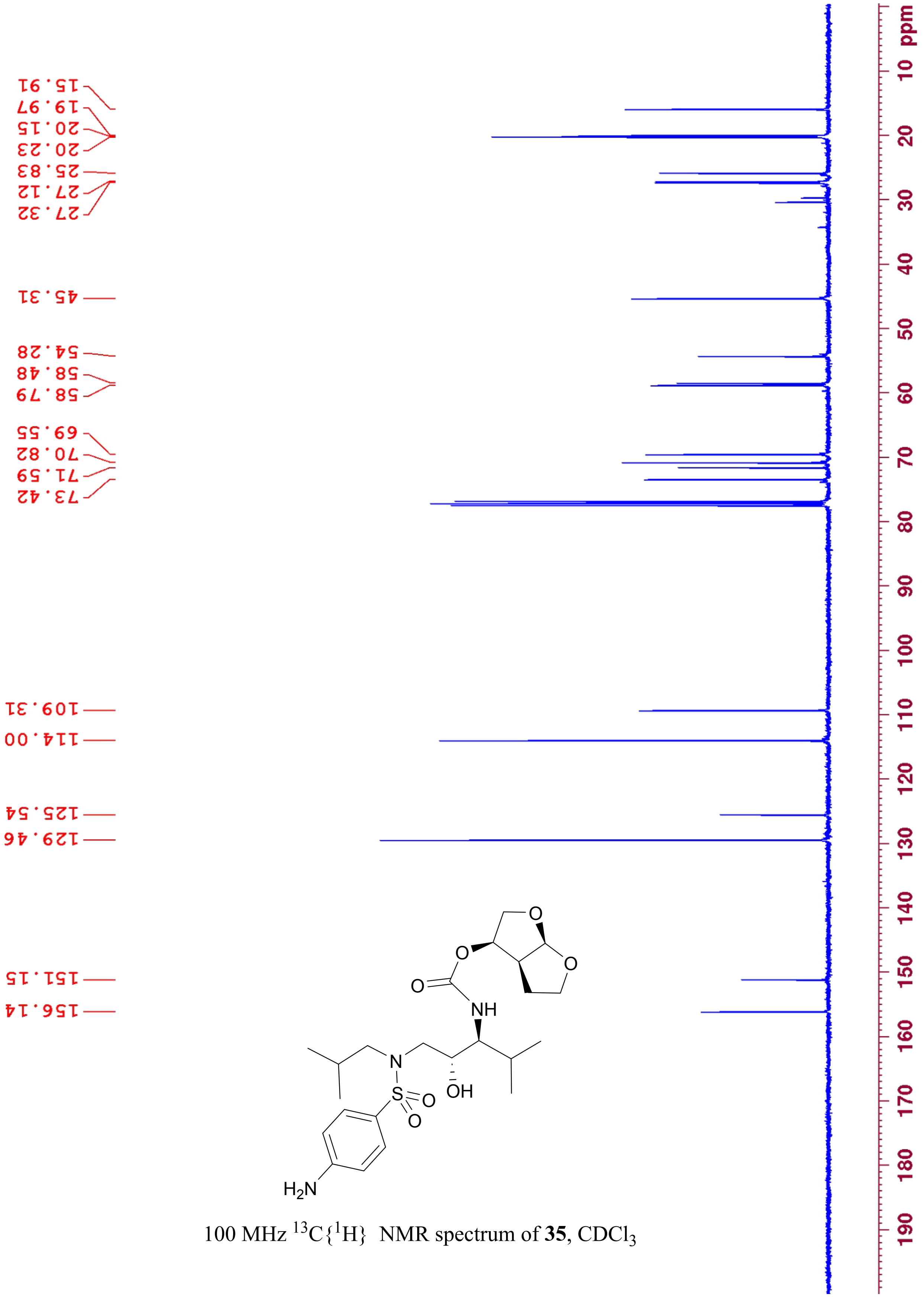

100 MHz $^{13}C\{^1H\}$ NMR spectrum of **35**, $CDCl_3$

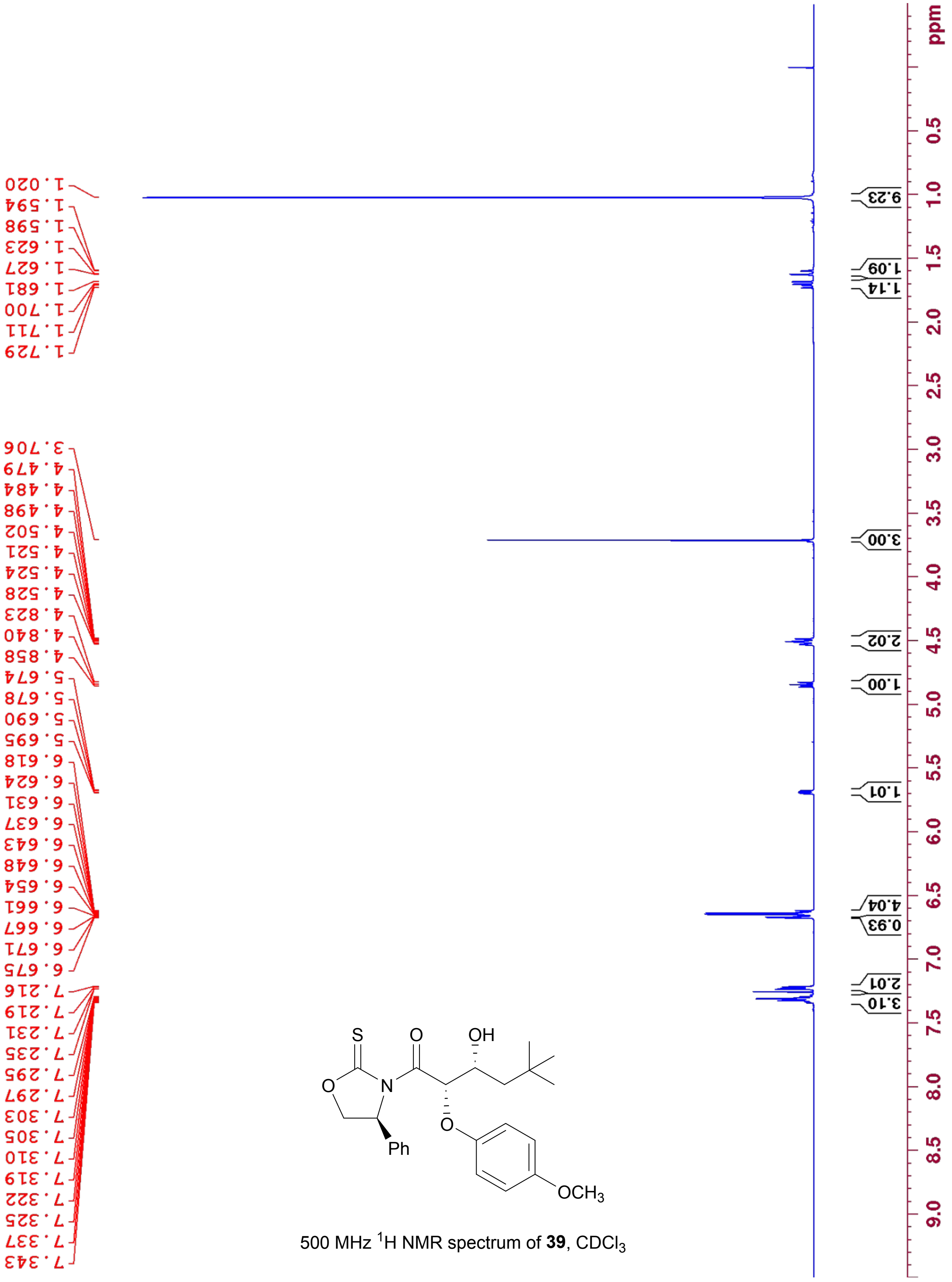

500 MHz ^{1}H NMR spectrum of **39**, $CDCl_3$

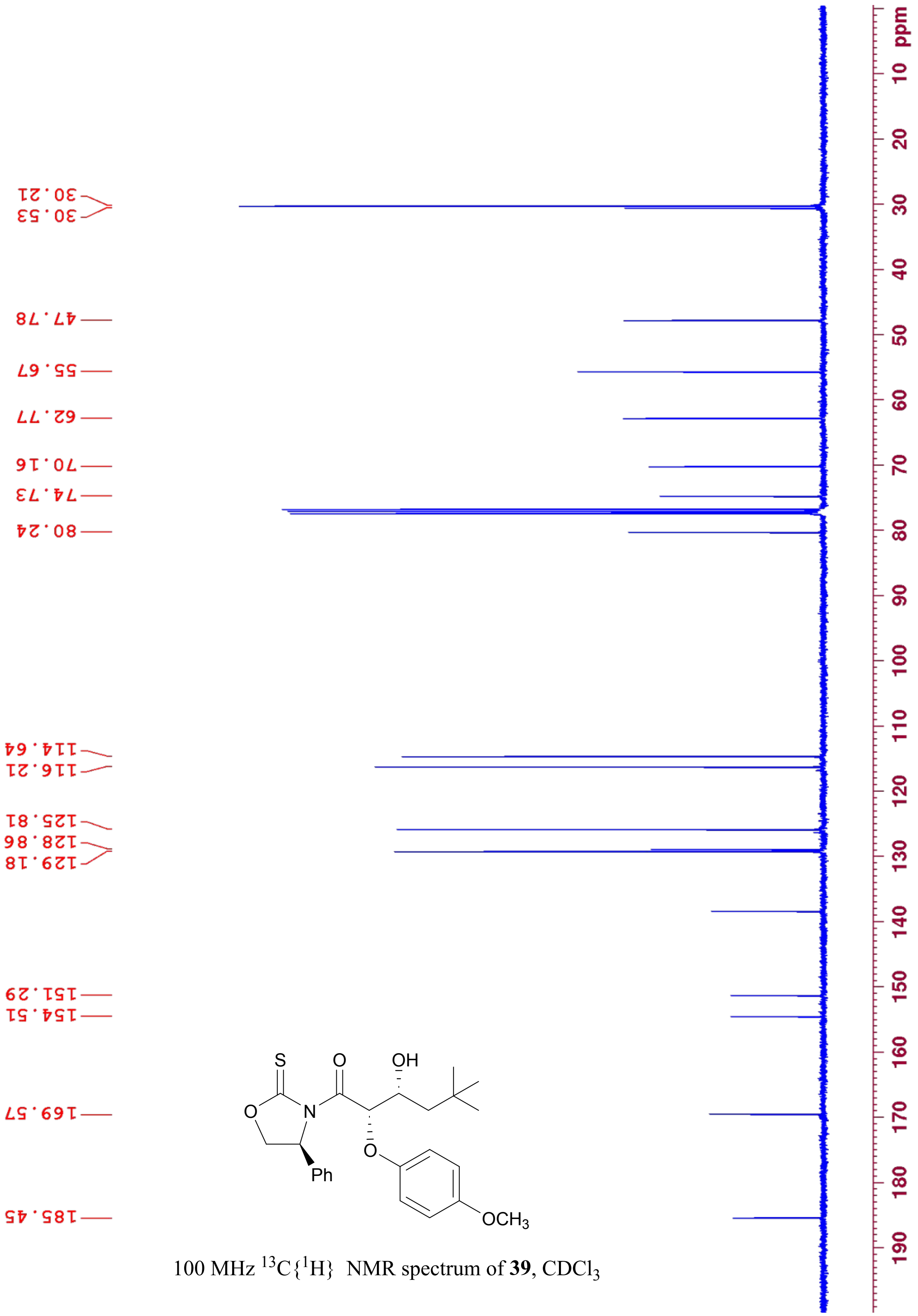

100 MHz $^{13}C\{^1H\}$ NMR spectrum of **39**, $CDCl_3$

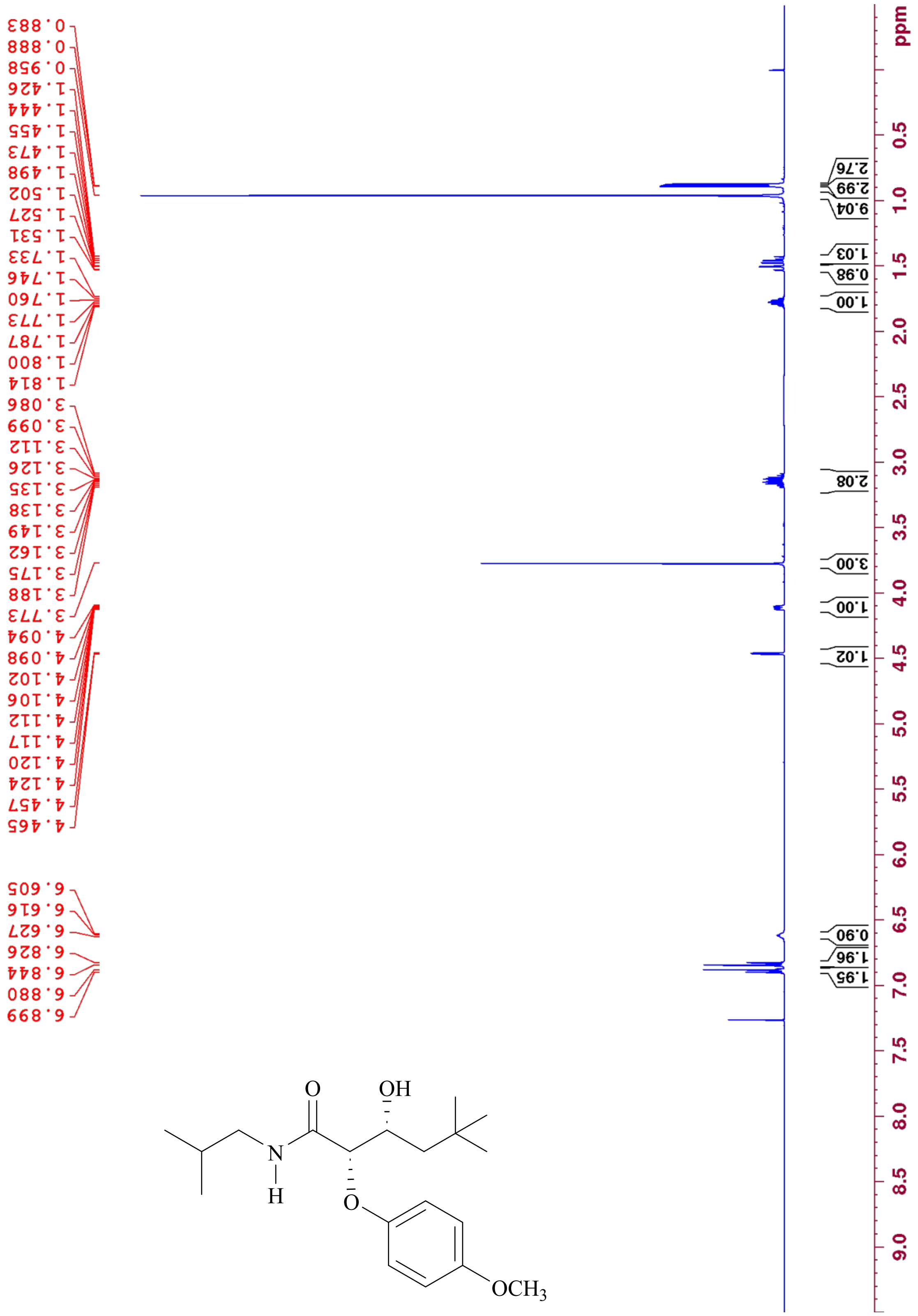

500 MHz ^{1}H NMR spectrum of **41**, $CDCl_3$

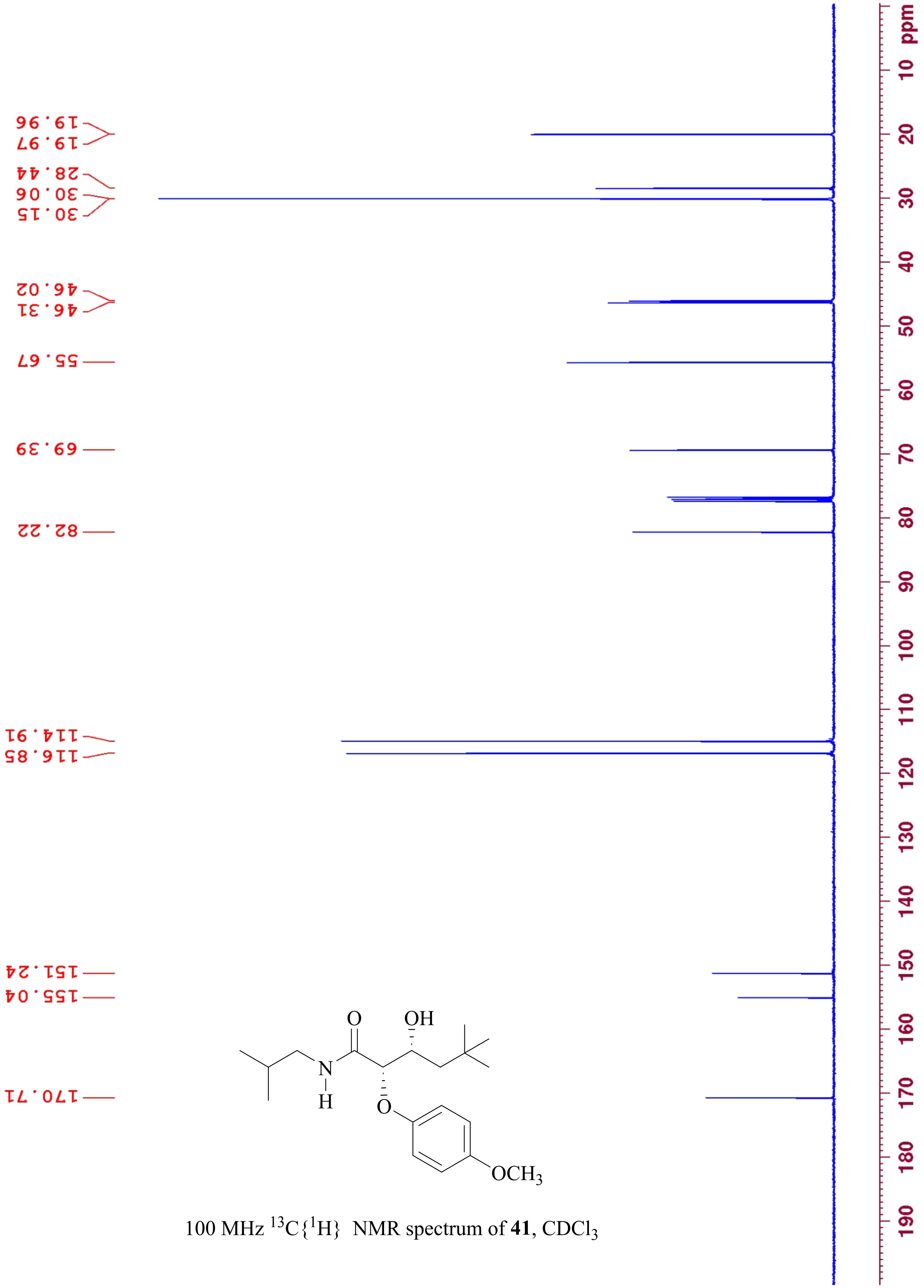

100 MHz $^{13}C\{^{1}H\}$ NMR spectrum of **41**, $CDCl_3$

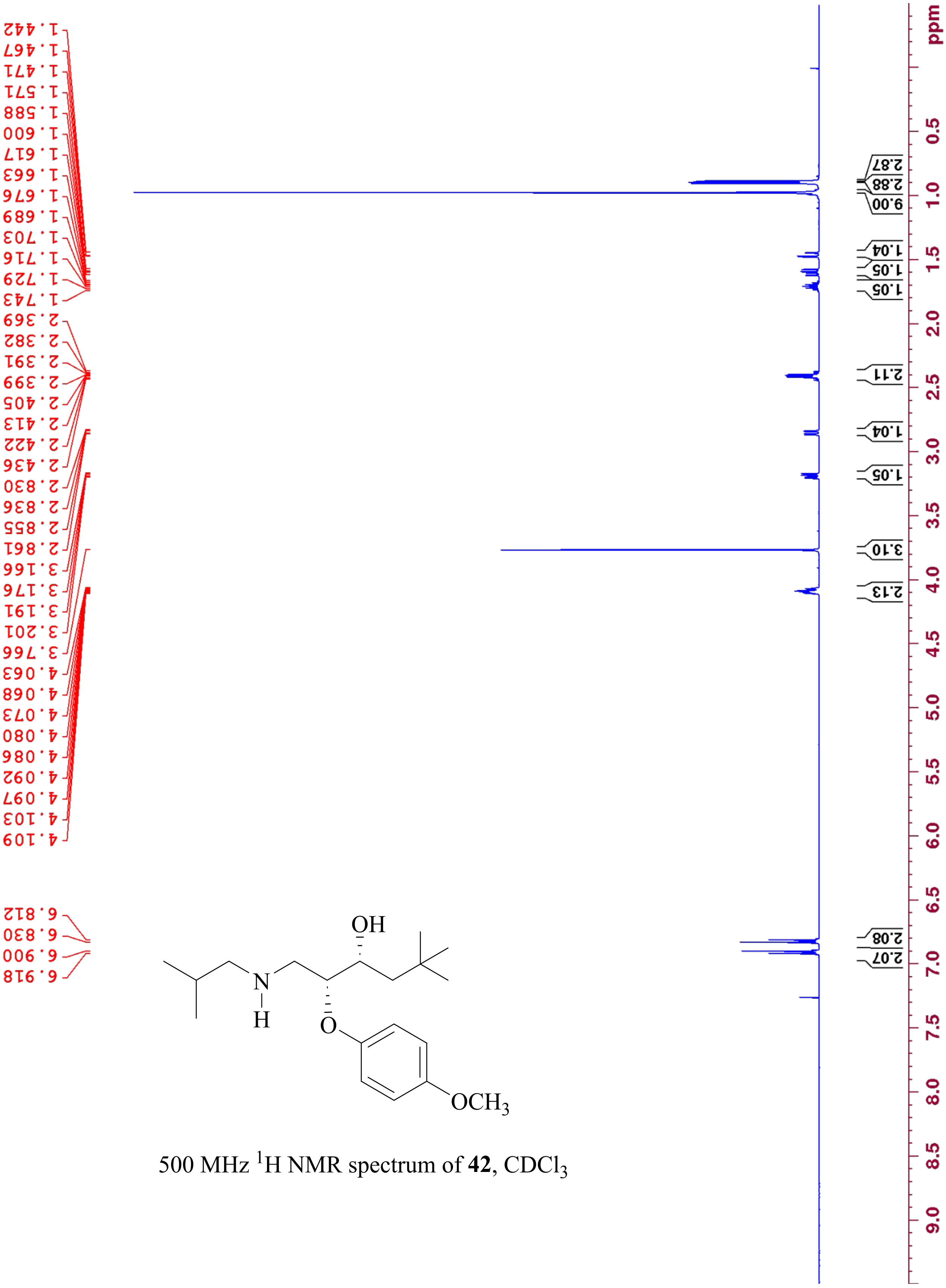

500 MHz ^{1}H NMR spectrum of **42**, $CDCl_3$

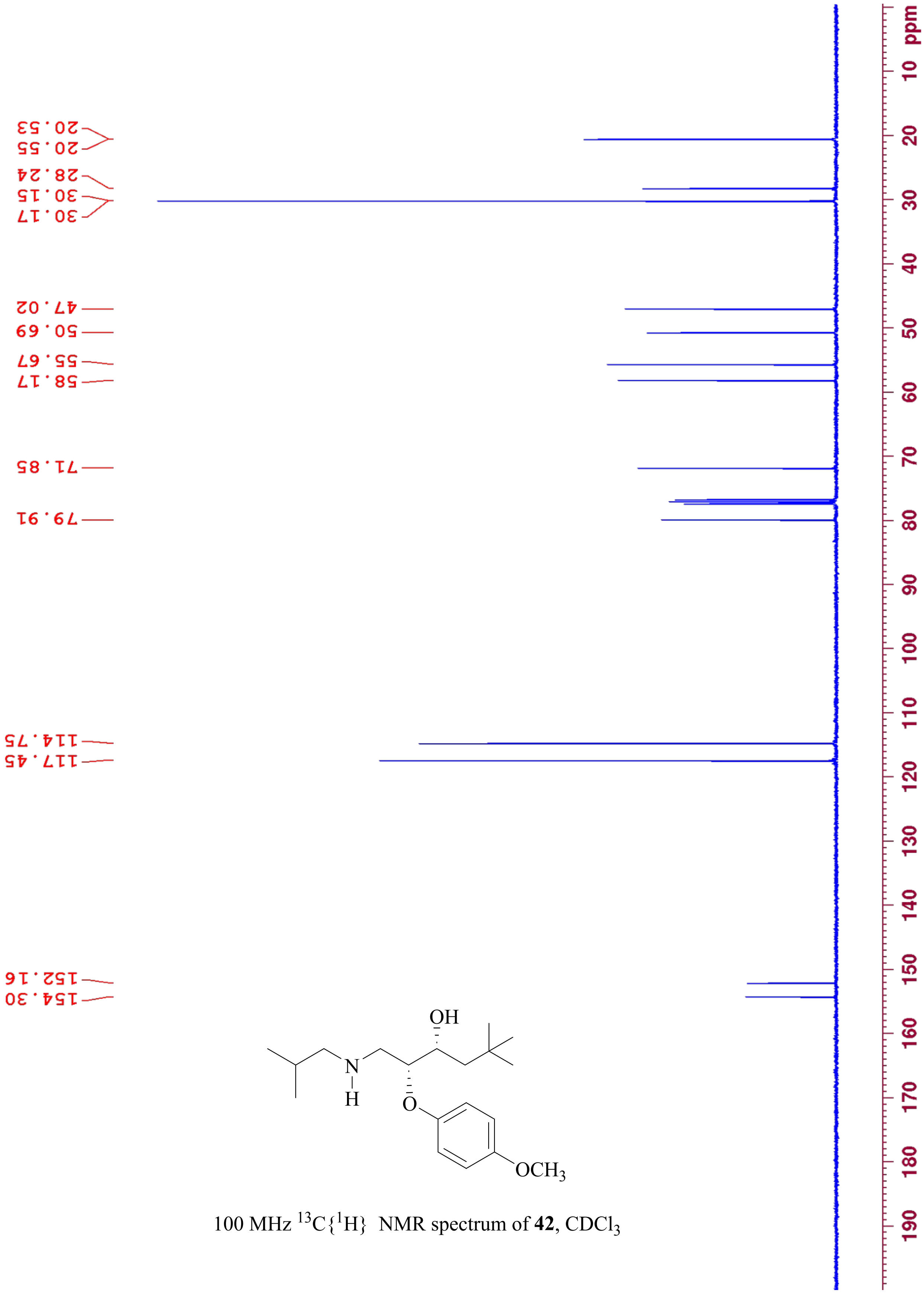

100 MHz $^{13}C\{^{1}H\}$ NMR spectrum of **42**, $CDCl_3$

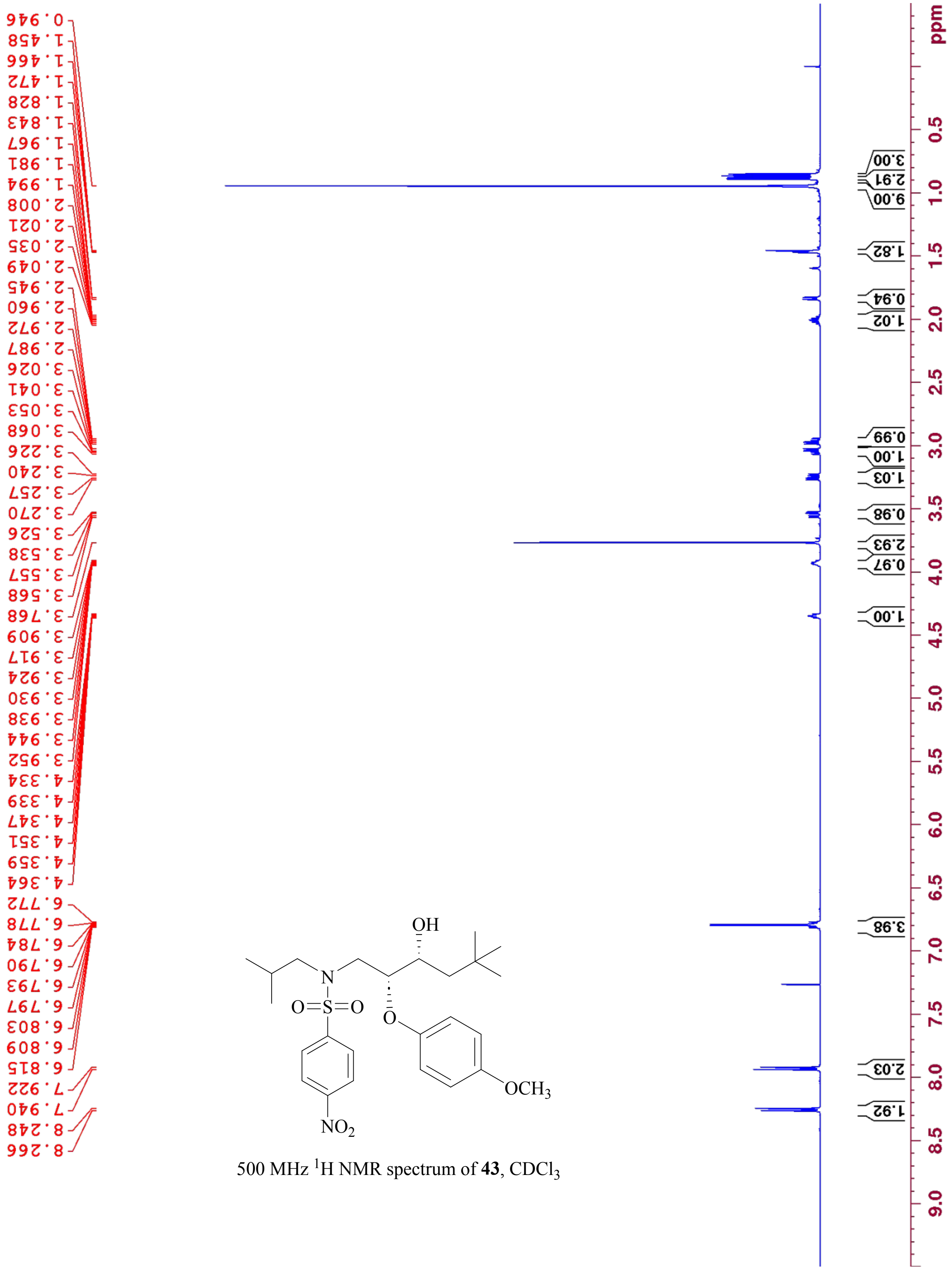

500 MHz ^{1}H NMR spectrum of **43**, $CDCl_3$

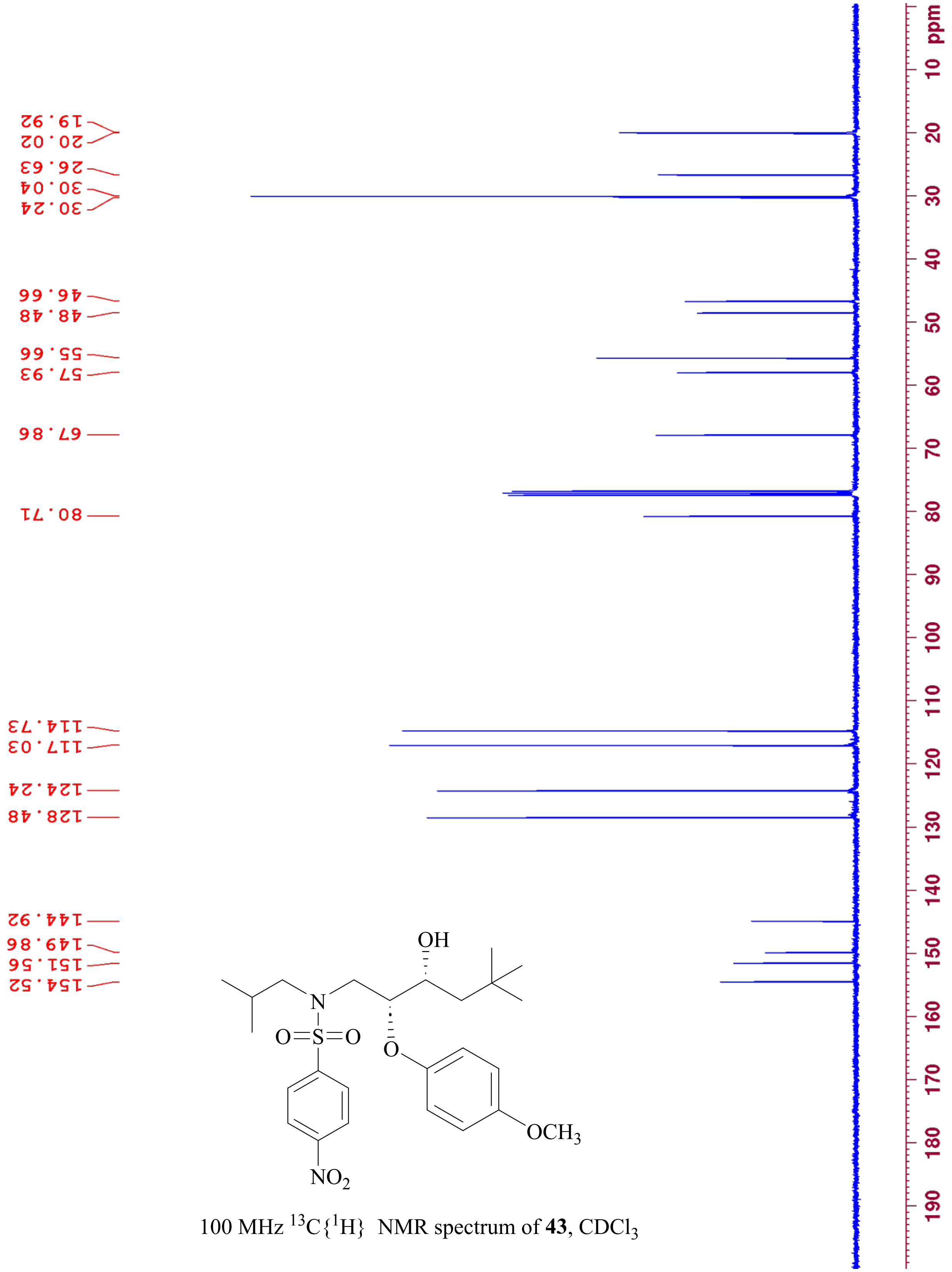

100 MHz $^{13}C\{^{1}H\}$ NMR spectrum of **43**, $CDCl_3$

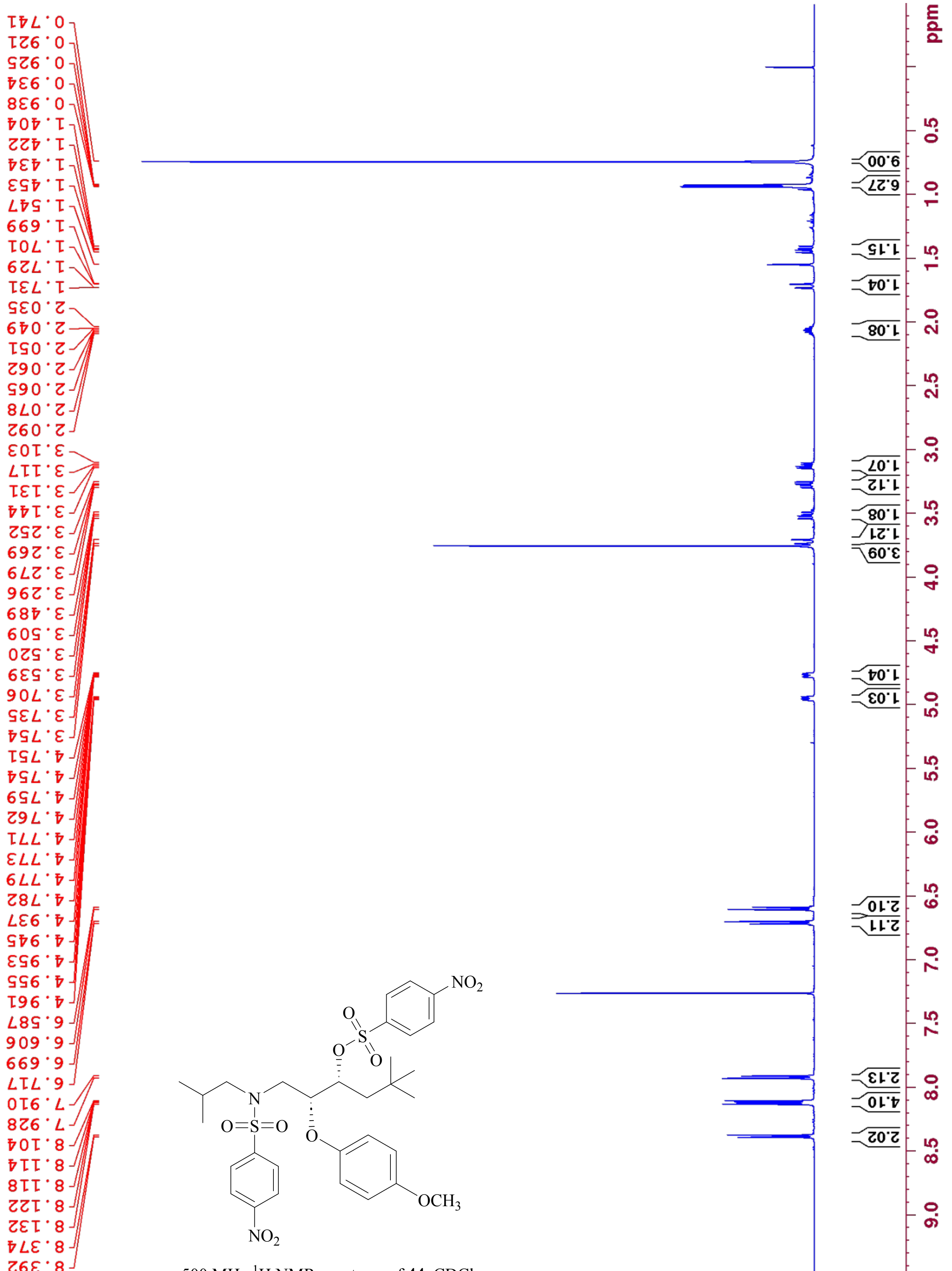

500 MHz ^{1}H NMR spectrum of **44**, $CDCl_3$

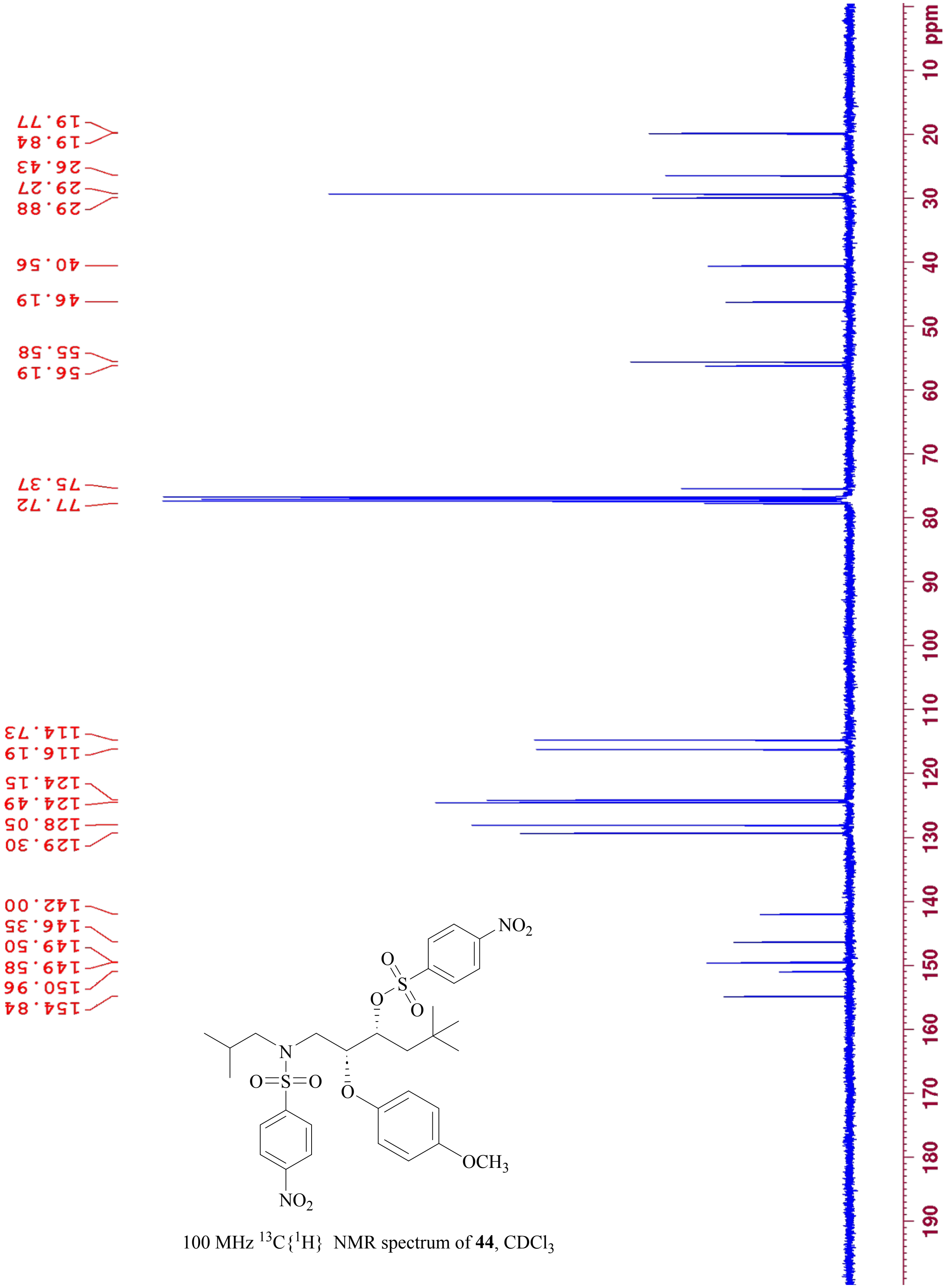

100 MHz $^{13}C\{^{1}H\}$ NMR spectrum of **44**, $CDCl_3$

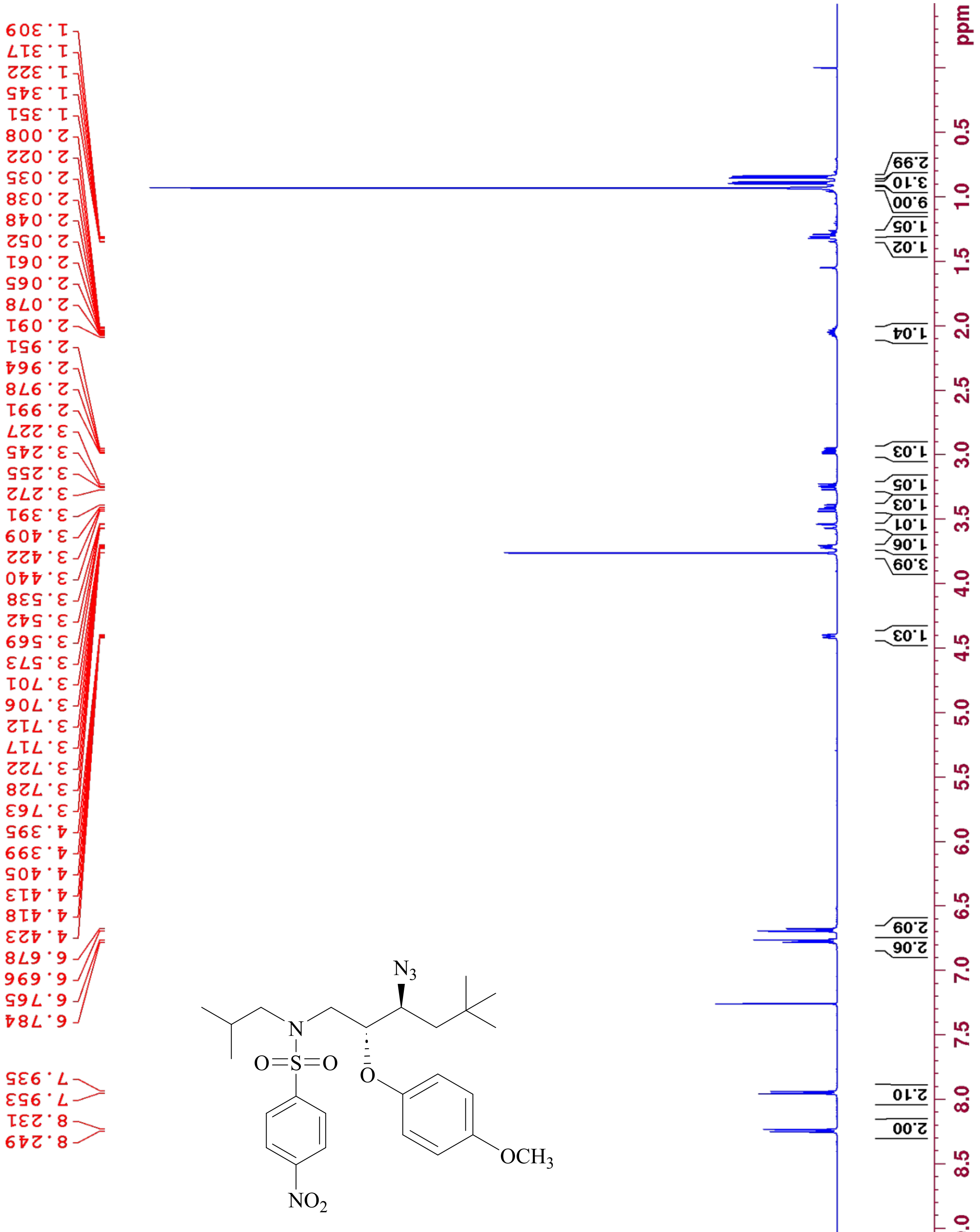

500 MHz ^{1}H NMR spectrum of **45**, $CDCl_3$

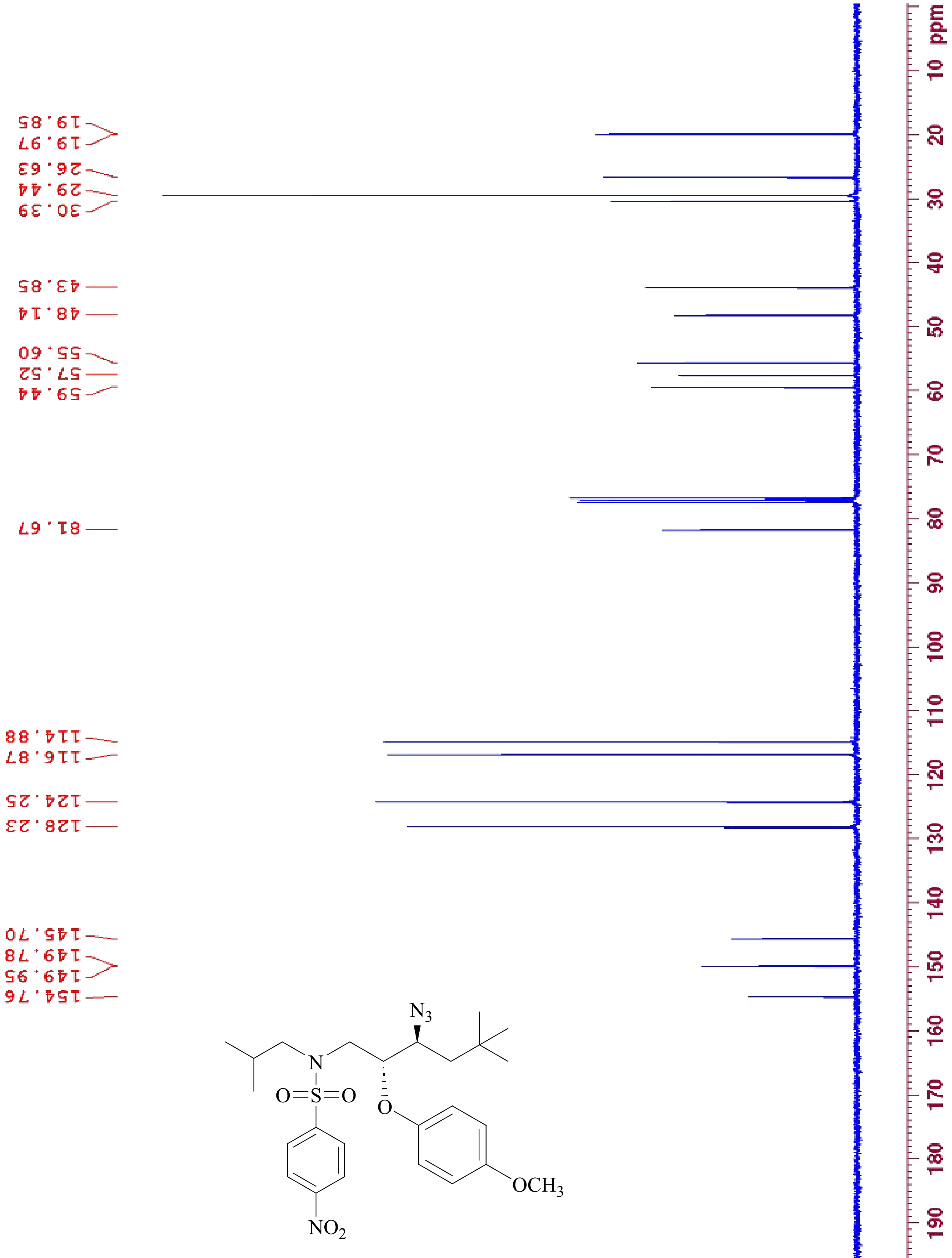

100 MHz $^{13}C\{^{1}H\}$ NMR spectrum of **45**, $CDCl_3$

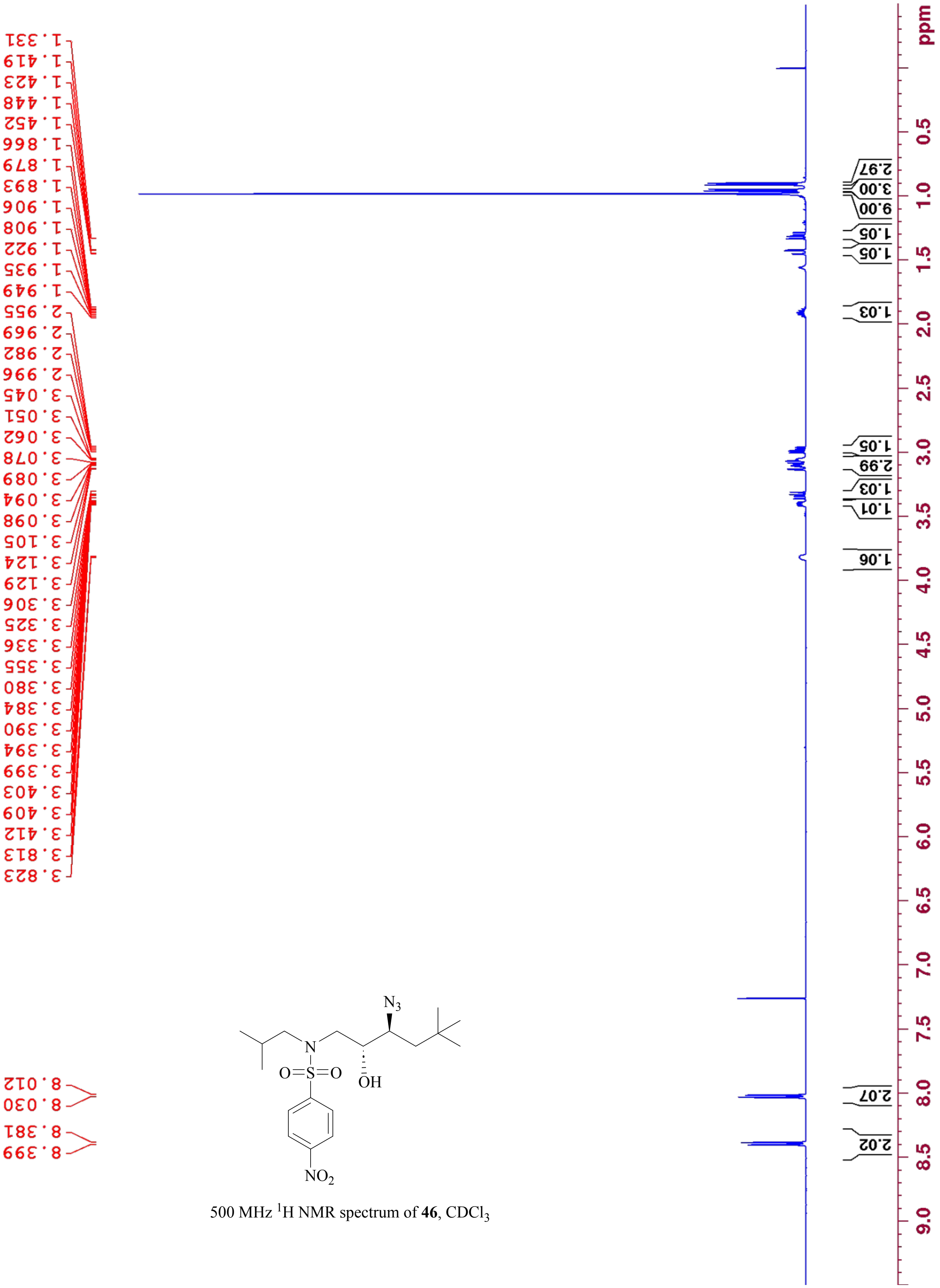

500 MHz ^{1}H NMR spectrum of **46**, $CDCl_3$

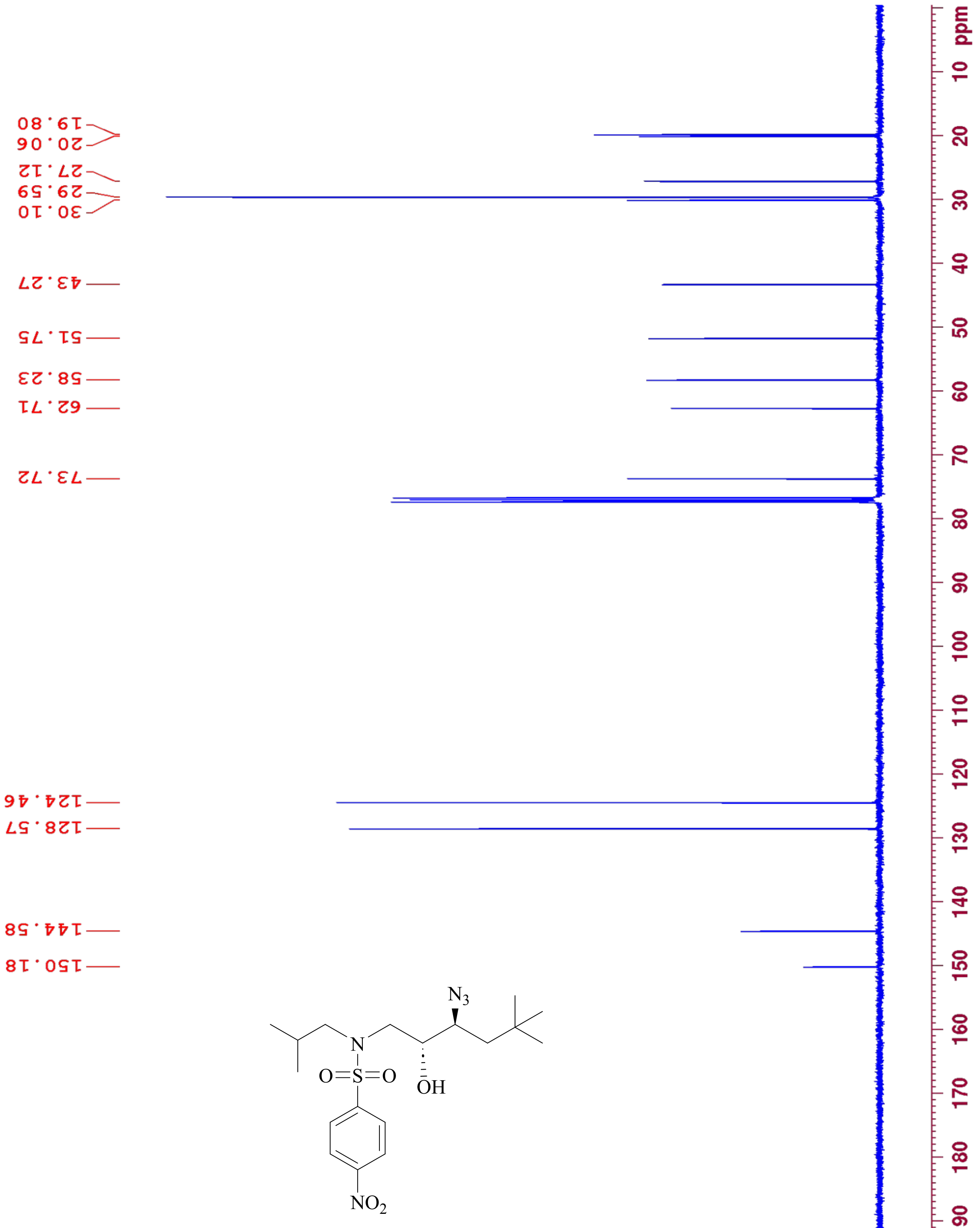

100 MHz $^{13}C\{^{1}H\}$ NMR spectrum of **46**, $CDCl_3$

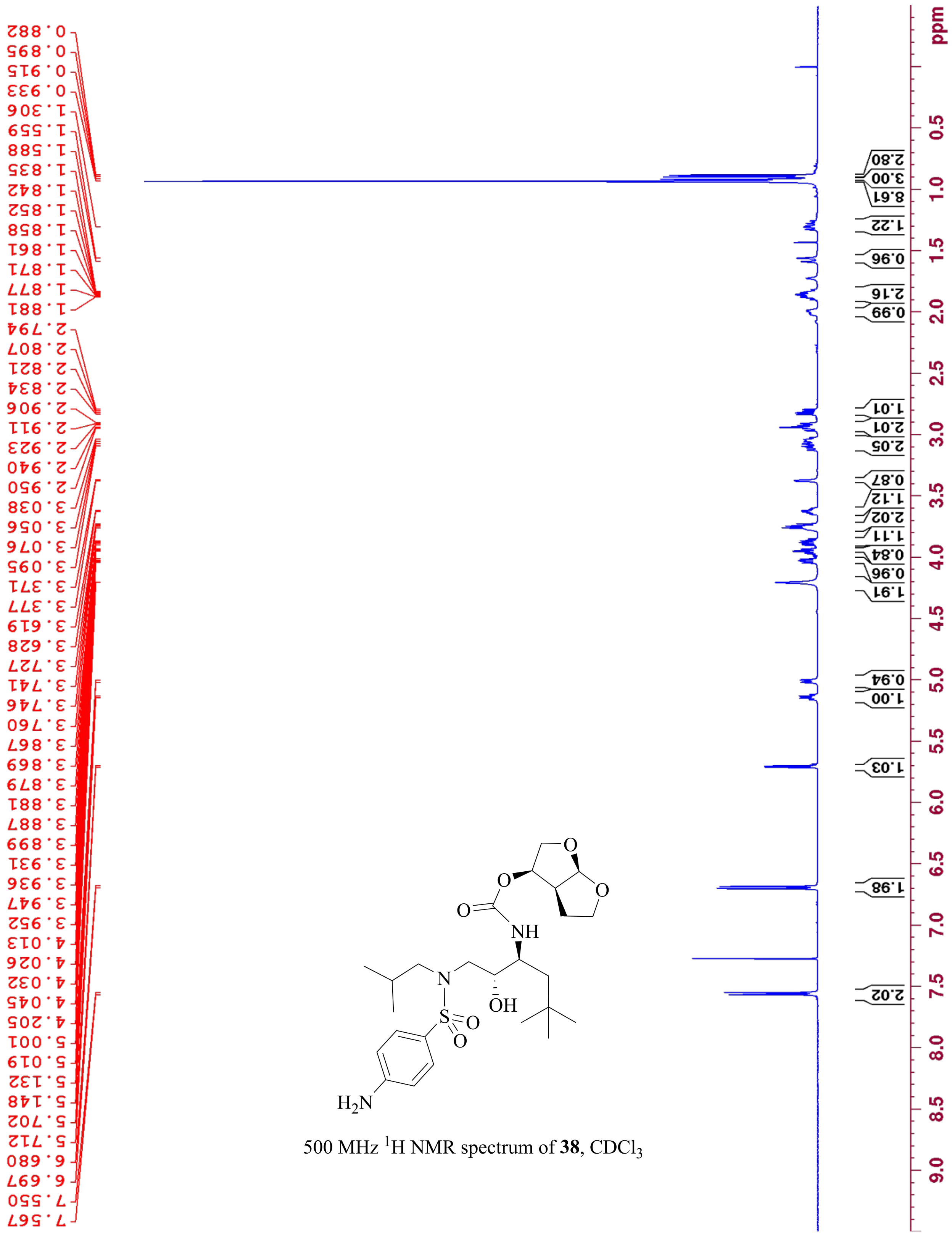

500 MHz ^{1}H NMR spectrum of **38**, $CDCl_3$

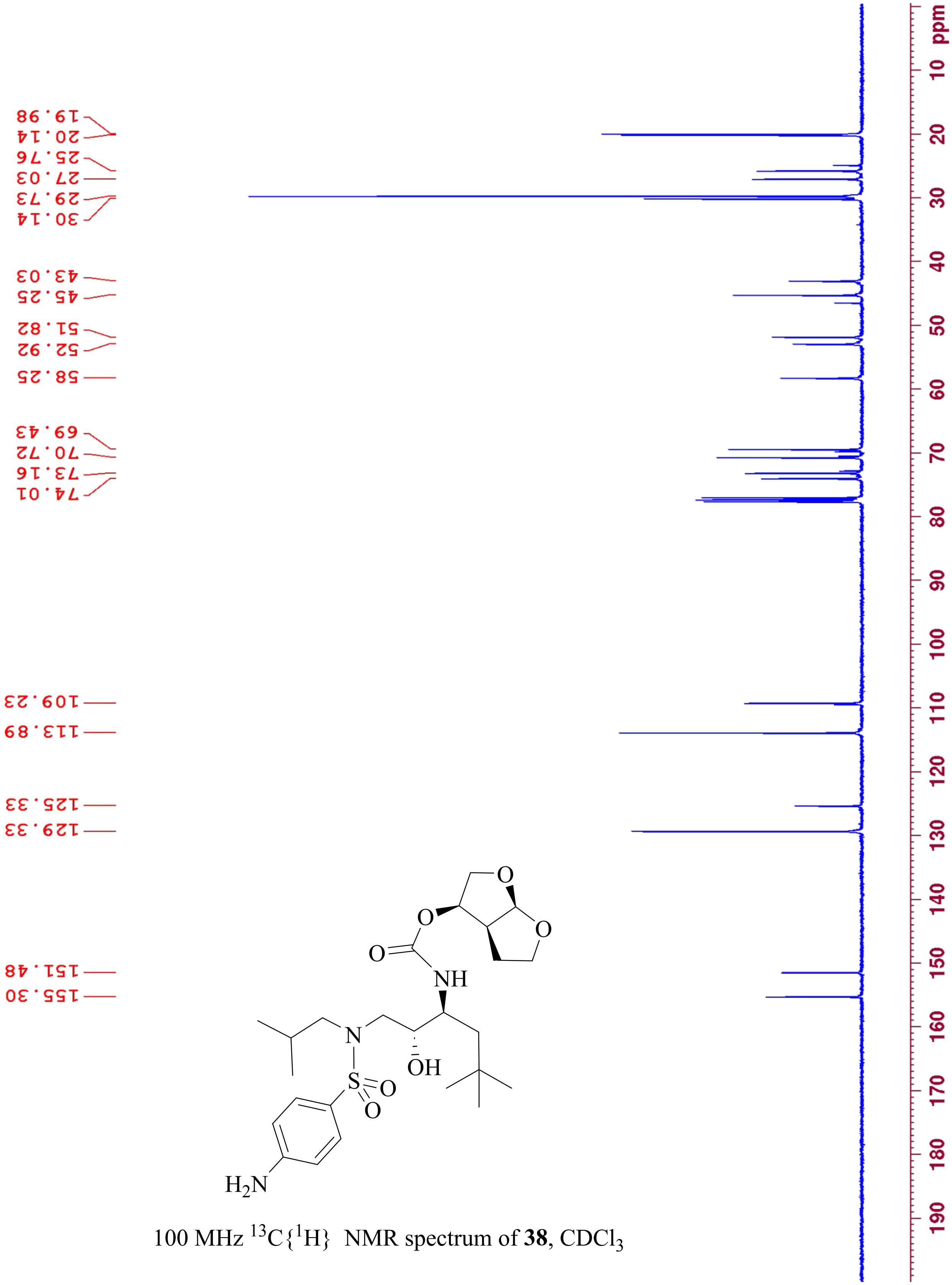

100 MHz $^{13}C\{^1H\}$ NMR spectrum of **38**, $CDCl_3$

General Crystallographic Experimental Details of (*R*,*S*,*R*)-10

Samples of X-ray quality crystals were suspended in mineral oil at ambient temperature and a suitable crystal was selected, mounted on a MiTeGen Micromount and transferred to a Bruker AXS SMART APEXII CCD X-ray diffractometer. The X-ray diffraction data were collected at 100(2) K using Mo-Kα (λ = 0.71073 Å) radiation. The frames were integrated with the Bruker SAINT software package using a narrow-frame algorithm.[1] Data were corrected for absorption effects using the Multi-Scan method (SADABS).[1] Structure were solved and refined using the Bruker SHELXTL Software Package,[2] Molecular diagrams were generated using Mercury.[3]

Specifically, slow vapor diffusion of pentane into a solution of **10** dissolved in ethyl acetate led to formation of SCXRD quality crystals within 24 hours. A mineral oil coated clear colourless plate-like specimen of **10** ($C_{26}H_{25}NO_5S$), with approximate dimensions 0.122 mm x 0.273 mm x 0.312 mm, was harvested and placed in the 100(2) K cryogenic stream on the goniometer. The X-ray intensity data were measured. A total of 3672 frames were collected. The total exposure time was 4.08 hours. The integration of the frame data using a triclinic unit cell yielded a total of 45216 reflections to a maximum θ angle of 33.10° (0.65 Å resolution), of which 7908 were independent (average redundancy 5.718, completeness = 95.8%, R_{int} = 5.12%, R_{sig} = 4.41%) and 6792 (85.89%) were greater than $2\sigma(F^2)$. The final cell constants of a = 10.5787(3) Å, b = 10.2358(3) Å, c = 10.8871(3) Å, β = 109.878(2)°, volume = 1108.63(6) Å^3, are based upon the refinement of the XYZ-centroids of 7908 reflections above 20 $\sigma(I)$ with 4.638° < 2θ < 59.13°. Data were corrected for absorption effects. The ratio of minimum to maximum apparent transmission was 0.938. The calculated minimum and maximum transmission coefficients (based

on crystal size) are 0.9440 and 0.97800. The structure was solved and refined using the space group $P2_1$, with $Z = 2$ for the formula unit, $C_{26}H_{25}NO_5S$. All non-H atoms were refined anisotropically. All H atoms were identifiable in the difference Fourier, but with the exception of the H atom attached to O, were included in the final refinement using the riding-model approximation. d(C--H) = 0.93 Å, $U_{iso} = 1.2U_{eq}(C)$ for aromatic, 0.97 Å , $U_{iso} = 1.2\ U_{eq}(C)$ for methylene, 0.98 Å, $U_{iso} = 1.2\ U_{eq}(C)$ for methine, and 0.96 Å, $U_{iso} = 1.5\ U_{eq}(C)$ for methyl H atoms. Coordinates of the H atom attached to the O atom were freely refined with $U_{iso} = 1.5U_{eq}(O)$. The final anisotropic full-matrix least-squares refinement on F^2 with 302 variables converged at $R_1 = 4.01\%$, for the observed data and $wR_2 = 9.35\%$ for all data. The goodness-of-fit was 1.026. The largest peak in the final difference electron density synthesis was 0.545 $e^-/Å^3$ and the largest hole was a – 0.393 $e^-/Å^3$ with an RMS deviation of 0.053 $e^-/Å^3$. On the basis of the final model, the calculated density was 1.389 g/cm^3 and F(000), 488 e^-. The absolute configuration was confirmed using 2811 quotients in determination of a Flack parameter of 0.10(2). All residual electron density was within accepted norms and was deemed of no chemical significance.

CCDC-2285488 contains the supplementary crystallographic data for this structure. These data can be obtained free of charge from the Cambridge Crystallographic Data Centre via https://summary.ccdc.cam.ac.uk/structure-summary?ccdc=2285488.

1. Bruker, *APEX4 v2021.10-0*, Bruker AXS Inc., Madison, Wisconsin, USA, 2021.

2. Sheldrick, G. M. Crystal Structure Refinement with SHELXL. *Acta Cryst.* **2015**, *C71*, 3-8.

3. Macrae, C. F.; Sovago, I.; Cottrell, S. J.; Galek,P. T. A.; McCabe, P.; Pidcock, E.; Platings, M.; Shields, G. P.; Stevens, J. S.; Towler, M.; Wood, P. A. Mercury 4.0: from Visualization to Analysis, Design and Prediction. *J. Appl. Cryst.* **2020**, *53*, 226-235.

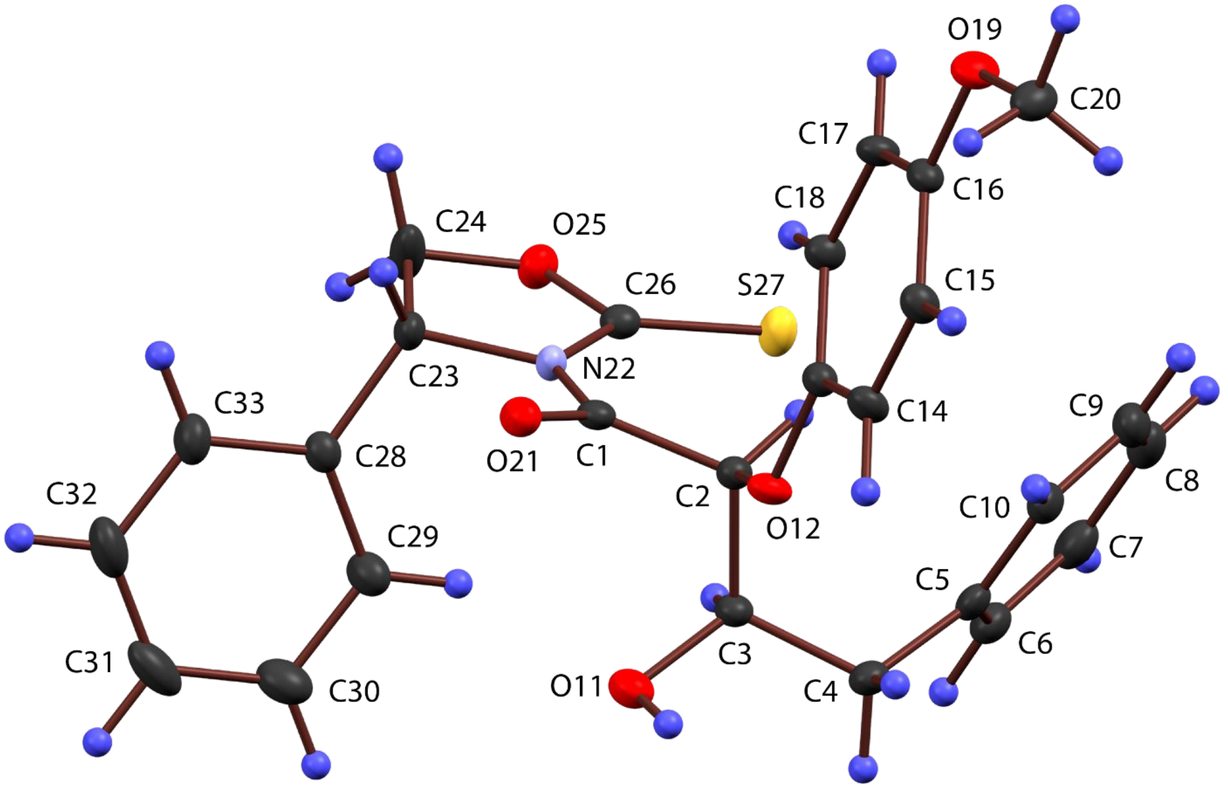

Figure S-1: X-ray crystal structure ORTEP rendering of **10**. The ellipsoids of all non-hydrogen atoms are shown at the 50% probability level; hydrogen atoms are drawn arbitrarily small.
